# Pulmonary Toxicity of Silica Linked to Its Micro- or Nanometric Particle Size and Crystal Structure: A Review

**DOI:** 10.3390/nano12142392

**Published:** 2022-07-13

**Authors:** Vanessa Marques Da Silva, Manon Benjdir, Pierrick Montagne, Jean-Claude Pairon, Sophie Lanone, Pascal Andujar

**Affiliations:** 1 University Paris Est Créteil, INSERM, Institut Mondor de Recherche Biomédicale, F-94010 Créteil, France; vanessa.marques-da-silva@inserm.fr (V.M.D.S.); manonbn@gmail.com (M.B.); montagnepierrick@gmail.com (P.M.); jc.pairon@chicreteil.fr (J.-C.P.); sophie.lanone@inserm.fr (S.L.); 2 Hôpital Intercommunal de Créteil, Service de Pathologies Professionnelles et de l’Environnement, F-94010 Créteil, France

**Keywords:** silica, silicon dioxide, amorphous silica, crystalline silica, quartz, nanoparticles, particles, lung, toxicity, experimental studies

## Abstract

Silicon dioxide (SiO_2_) is a mineral compound present in the Earth’s crust in two mineral forms: crystalline and amorphous. Based on epidemiological and/or biological evidence, the pulmonary effects of crystalline silica are considered well understood, with the development of silicosis, emphysema, chronic bronchitis, or chronic obstructive pulmonary disease. The structure and capacity to trigger oxidative stress are recognized as relevant determinants in crystalline silica’s toxicity. In contrast, natural amorphous silica was long considered nontoxic, and was often used as a negative control in experimental studies. However, as manufactured amorphous silica nanoparticles (or nanosilica or SiNP) are becoming widely used in industrial applications, these paradigms must now be reconsidered at the nanoscale (<100 nm). Indeed, recent experimental studies appear to point towards significant toxicity of manufactured amorphous silica nanoparticles similar to that of micrometric crystalline silica. In this article, we present an extensive review of the nontumoral pulmonary effects of silica based on in vitro and in vivo experimental studies. The findings of this review are presented both for micro- and nanoscale particles, but also based on the crystalline structure of the silica particles.

## 1. Introduction

Silica is the common name given to materials composed of silicon dioxide (SiO_2_), which exist in crystalline (including α- and β-quartz, cristobalite, and tridymite) or amorphous forms [1]. Upon heating, α-quartz is transformed into β-quartz, tridymite, and cristobalite. Quartz is the most common form of crystalline silica and exists in both natural and synthetic forms (notably the porous synthetic crystalline silica family, porosil). Amorphous silica can be subdivided according to its natural (e.g., diatomaceous earth) or anthropic origins (e.g., fumed or precipitated silica). The toxicity of silica has been linked to its crystallinity and capacity to generate free radicals. Occupational exposure of miners and construction workers to micrometric crystalline silica particles (0.5–10 µm) has been extensively studied. This exposure is associated with an increased risk of developing lung diseases such as silicosis (pneumoconiosis characterized by interstitial lung inflammation and fibrotic granuloma, also known as silicotic nodules), chronic bronchitis, chronic obstructive pulmonary disease, emphysema, pulmonary tuberculosis, and even lung cancer in some studies [2,3,4]. Extrapulmonary diseases, such as rheumatoid arthritis or systemic scleroderma, have also been associated with exposure to crystalline silica [5]. In contrast, until now, naturally occurring amorphous micrometric silica has generally been considered less harmful because of its structure [6,7]. Some studies reported pneumoconiosis among diatomaceous earth workers, but these cases were due to contamination of amorphous silica with crystalline silica [6,8]. This contamination is therefore a major problem when attempting to assess the toxic effects of amorphous micrometric silica [6].

**Figure 1:**
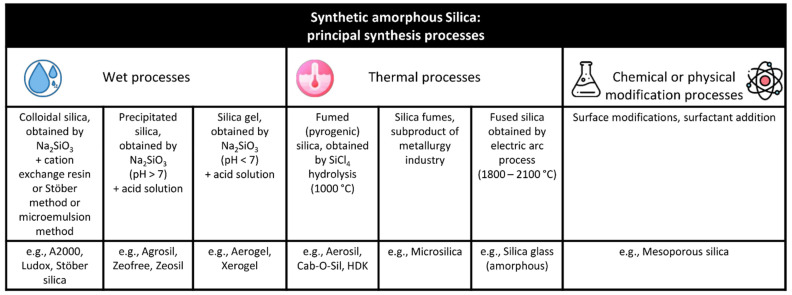
Processes by which amorphous silica is synthesized.

The advent of nanotechnology now makes it possible to produce manufactured amorphous silica nanoparticles (NPs) (<100 nm) in various forms without any crystalline silica contamination. These synthetic forms of amorphous silica may be classified based on the processes used to produce them: wet processes (colloidal silica, precipitated silica, or silica gel), thermal processes (pyrogenic silica, silica fumes, fused silica), and chemically or physically surface-modified silica (e.g., mesoporous silica) (Figure 1). In line with their distinct physicochemical properties, manufactured amorphous silica NPs are used in a variety of products. For example, these engineered nanomaterials are incorporated as fillers in paints, rubber (e.g., in tires), as anticaking agents in powdered materials, or as additives in animal and human food, agrochemical products, toothpastes, silicones (e.g., in hair conditioners), building materials (e.g., in insulation, coatings and adhesives), printing inks or cosmetics [9]. Some in vivo and in vitro experimental studies seem to suggest that amorphous silica NPs display similar toxicity to micrometric crystalline silica [10]. This similarity in effects may be linked to the extensive involvement of surface chemistry (i.e., the conformation of silanols and siloxanes). This hypothesis partly overturns the old dogma relating to silica toxicity that was solely based on its crystalline structure [9,11,12]. With this review, we aim to summarize current knowledge on the nontumoral lung effects of exposure to crystalline and amorphous silica at micro- and nanometric scales.

## 2. Materials and Methods

### 2.1. Search Strategy

**Figure 2:**
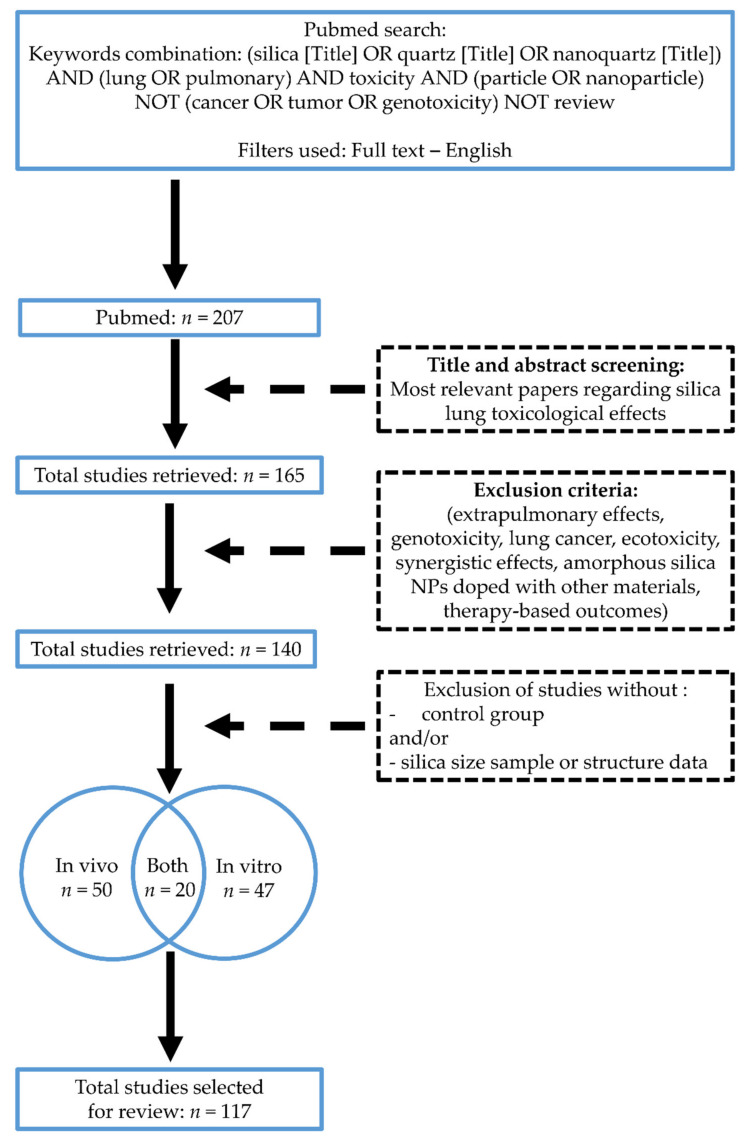
Article-selection flow chart.

English-language papers published from 1 January 1980 to 31 December 2021 were identified through the MEDLINE database https://pubmed-ncbi-nlm-nih-gov.proxy.insermbiblio.inist.fr/ (accessed on 31 January 2022). The following keyword combination was used: “(silica [Title] OR quartz [Title] OR nanoquartz [Title]) AND (lung OR pulmonary) AND toxicity AND (particle OR nanoparticle) NOT (cancer OR tumor OR genotoxicity) NOT review. We also used these following filters: “Full text—English” (Figure 2).

### 2.2. Selection Criteria

The selection criteria applied to the articles identified were as follows, the article had to present: (a) a detailed description of the physicochemical characteristics of the silica used, such as primary size, composition, and crystallinity; (b) toxicological endpoints for in vitro experimental studies, such as inflammation, cytotoxicity, apoptosis, necrosis and/or autophagy, oxidative stress, and immunotoxicity using immortalized cell lines or primary cells; and/or (c) toxic effects in laboratory animals specifically relating to in vivo experiments using rodents. From each paper selected, we extracted the information relating to the physicochemical characteristics of silica, the type of cellular or animal model, and the toxicological endpoints (Tables S1–S7). The main micrometric crystalline silica particles used are quartz particles [e.g., Min-U-Sil from U.S. Silica Corporation or Pennsylvania Glass Sand Corporation (Katy, TX, USA)), S5631 from Sigma-Aldrich (St. Louis, MO, USA), DQ-12 from DMT GmbH and Co (Essen, Germany), IUF Dusseldorf, Institute for Occupational Safety and Health of German Social Accident Insurance, Dorentruper Sand und Thonwerke GmbH of Dorentrup, Crystalline Dowson and Dobson quartz (generous gift from Dr. J.H. Bachmann, Johannesburg, South Africa), α-quartz (such as Sikron F600 from National Institute of Occupational Health or Poison Control, Beijing, China), Norquartz-45 from Glamsland, Norway, or freshly or aged fractured quartz from Generic Respirable Dust Technology Center, USA]. The other type of micrometric crystalline silica particles used is α-cristobalite (from C&E Mineral Corporation, King of Prussia, PA, USA). The only nanometric crystalline silica particle used is hydrothermally synthesized nanoquartz. For amorphous silica, the main micrometric particles used are colloidal silica (e.g., fine colloidal silica from Fuso Chemical Corporation, Osaka, Japan), precipitated silica (e.g., Zeofree80 from JM Huber Corporation, Edison, NJ, USA), fumed silica (e.g., Aerosil from Sigma), or modified surface particles. As described in Figure 1, the main nanometric amorphous silica particles used are precipitated silica (e.g., Pre20), colloidal silica (e.g., NexSil20 from Nyacol Nano Technologies, Ashland, MA, USA, SM30 or TM-40 Ludox from Sigma-Aldrich, ultrafine colloidal silica from Fuso Chemical Corporation, Col15 and Col40/80), fumed silica (pyrogenic) (e.g., Aerosil from Degussa, Francfort, Germany or Sigma-Aldrich, Pyr20 or Pyr25/70), or mesoporous SiNPs (from NanoAmor, Houston, TX, USA or Sigma).

### 2.3. Exclusion Criteria

To specifically focus on nontumoral adverse pulmonary effects linked to silica, papers reporting on other aspects such as extrapulmonary effects, genotoxicity, lung cancer, ecotoxicity, synergistic effects, amorphous silica NPs doped with other materials, and therapy-based outcomes were excluded [13]. We also excluded articles that did not include a control group.

### 2.4. Selection of Papers for Review

We thus examined in three stages the papers returned by these searches (Figure 2). The first step retrieved 207 articles after application of the previously defined keyword combinations and filters. The second step involved screening the title and abstract of each article to identify the studies that best matched our search terms; 165 relevant papers were retained. The third step was a full analysis of each article. During this step, the exclusion criteria applied to 25 articles. Any papers for which suitability was unclear during the second or third step were discussed by P. Andujar and V. Marques Da Silva until a consensus was reached. Finally, 117 articles reporting a minimal set of physicochemical characterization methods and toxic effects with control groups were retained for this review. The distribution was as follows: 50 articles reporting in vivo experimental studies, 47 articles presenting in vitro studies, and 20 articles including both in vivo and in vitro results.

## 3. Experimental Designs

### 3.1. In Vivo Experimental Studies in Rodents

In vivo experimental studies have mainly been conducted in rodent models (generally wild-type, but also transgenic, rats, mice or Syrian hamsters). Tables S1–S4 summarize the in vivo experimental studies that have been performed with each type of silica. Tables are organized by exposure route (whole-body inhalation, nose-only inhalation, intratracheal instillation, oropharyngeal aspiration, or intranasal instillation). The associated bullet lists summarize doses administered and exposure duration for each exposure route.

Referring to the OECD (Organization for Economic Cooperation and Development) guidelines [14], acute studies last less than 24 h; acute studies in rats investigating whole-body inhalation exposure generally last 4 h, or 6 h for nose-only exposure. Repeated exposure can be used to investigate adverse effects following daily or 5 times per week inhalation exposure to a chemical substance. Subacute and subchronic studies last at least 28 or 90 days, respectively (the latter covers approximately 10% of the lifespan of a rat) [14]. Thus, acute, subacute, subchronic, or chronic studies involve a range of durations: less than one day; from 2 to 28 days; from 29 to 90 days; and more than 90 days, respectively.

- Micrometric Crystalline Silica

In whole-body inhalation studies with micrometric crystalline silica, rodents were exposed to repeated high or very high doses, ranging from 6.2 to 100 mg/m^3^, over 10 to 90 days, generally for 6 h per day [15,16,17,18,19,20]. In contrast, in “nose-only” inhalation studies, rats were exposed to repeated doses of 15 mg/m^3^ for 21 to 59 days, also for 6 h per day [21,22]. Intratracheal instillations have mostly been performed as a single administration in animals, with cumulated doses ranging from 0.01 to 640 mg/kg, with observation timepoints covering the full range (acute, subacute or subchronic, or chronic) [23,24,25,26,27,28,29,30,31,32,33,34,35,36,37,38,39,40,41,42,43,44,45,46,47,48]. Oropharyngeal aspirations in mice were also mostly performed as a single administration of a very high dose (cumulated doses from 40 to 160 mg/kg), but only acute observation timepoints (1 to 56 days) were reported [49,50,51,52].

- Nanometric Crystalline Silica

In the only study we identified using nanoquartz [46], rats were exposed intratracheally with a single administration of between 1 and 5 mg/kg. Observation timepoints were acute or subchronic (from 1 to 90 days).

- Micrometric Amorphous Silica

In whole-body inhalation studies with micrometric amorphous silica, rats were exposed to repeated doses ranging from 10 to 150 mg/m^3^ over 10 to 90 days, for 6 h per day [20]. Intratracheal instillations were performed mainly as a single dose (0.03 to 150 mg/kg) in rodents [38,53,54,55,56,57,58]. Single-dose oropharyngeal aspirations (1.6 to 10 mg/kg) were administered to mice [54,59].

- Nanometric Amorphous Silica

In whole-body inhalation studies with nanometric amorphous silica, rats were exposed to repeated high or very high doses, from 10 to 150 mg/m^3^, over 10 to 90 days, for 6 h per day [60]. In “nose-only” inhalation studies, rats were exposed to ambient doses between 2.6 and 27 mg/m^3^, for 4 or 6 h per day, with observation timepoints covering the full range [26,43]. Intratracheal instillations in rodents were performed mainly as a single 0.02 to 160 mg/kg dose, and observation timepoints once again covered the full range (acute, subacute, subchronic, or chronic) [38,43,53,54,55,56,57,61,62,63,64,65,66,67,68].

### 3.2. In Vitro Experimental Studies

Tables S5–S7 summarize all the in vitro studies reviewed for each type of silica. Tables are organized by cell type (bronchial epithelial cell, macrophage, fibroblast) and species (e.g., human or rodent cells). Few reports presenting coculture results were found. As indicated above, no study relating to crystalline silica NPs was identified according to our criteria.

## 4. Biological Effects of Micro- and Nanometric Crystalline or Amorphous Silica

Table 1 (in vivo studies) and Table 2 (in vitro studies) summarize the biological effects according to the crystalline nature and size of silica particles implemented. Supplementary Tables S1–S7 present the biological effects reported in each article retained for review.

### 4.1. Acute and Chronic Inflammation

Inflammation is an immune response under the control of multiple regulatory systems; it is generally triggered in reaction to an exogenous or endogenous aggression. Inflammation is part of the natural immune response to a danger signal, and subsequently promotes the induction of a specific immune response. If the inflammatory response is inadequate or poorly controlled, it can become aggressive.

- Micrometric Crystalline Silica and Inflammation

Micrometric crystalline silica particles induce lung inflammation—triggering expression of numerous inflammatory markers and increased total cell counts in bronchoalveolar lavage fluid (BALF) [17,25,29,30,37,38,40,41,43,44,47,51], and activating inflammatory cell recruitment. In terms of cellular profile, significant increases in total phagocytic cells, such as neutrophils and alveolar macrophages, are reported [17,19,24,27,29,33,37,38,40,41,43,44,47,51,69]. These observations are confirmed in histological analyses, with significant recruitment of neutrophils and alveolar macrophages to the alveolar area [23]. Some studies also report a significant increase in dendritic cells [44] and lymphocytes in BALF [29,40]. Activation of cellular cytolysis has been reported based on multiple markers, such as increased lactate dehydrogenase (LDH) activity and elevated serum albumin concentrations [70]. In addition, analyses of protein expression revealed a significant increase in total protein levels, confirming an acute and subchronic inflammatory status [24,25,27,29,32,37,41,47,51]. Cytokine expression profiling, as analyzed by ELISA, Western blot, or flow cytometry, revealed increased levels of proinflammatory cytokines, such as TNF-α (tumor necrosis factor) [27,37,44,70], IL-1α (interleukin) [38], IL-1β [38], IL-6 [44], IL-18 [51], IL-33 [38], KC (keratinocyte chemoattractant) [37], MIP-2 (macrophage inflammatory protein 2) [70], and HMGB1 (high-mobility group box 1) [51]. Alveolar macrophages were shown to release proinflammatory cytokines, increasing vascular permeability and leading to the recruitment of inflammatory cells [37,38,44,51]. In rats exposed to micrometric crystalline silica, the recruitment of neutrophils to acutely inflamed alveolar regions involves several cytokines, including IL-1β, TNF-α, and KC (CXCL1 cytokine of the chemokine CXC motif ligand family) [58]. Cell types producing cytokines include macrophages, neutrophils, or epithelial cells [58]. Moreover, expression of the genes coding for IFN-γ (Interferon), IFN-α/β, and CXCL10 (or interferon-γ induced protein 10, IP-10) is reported to increase after exposure to micrometric crystalline silica [28,31]. According to the literature, CXCL10 is secreted by several cell types, such as monocytes, endothelial cells and fibroblasts, in response to IFN-γ. Several roles have been attributed to CXCL10, including acting as a chemoattractant for monocytes/macrophages.

Chronic inflammation was also investigated at timepoints ranging from 90 to 180 days in several in vivo studies involving single- or repeated-exposures. Exposure for more than 3 months to micrometric crystalline silica induces a significant increase in total cell count in BALF [29,47]. The specific cell types recruited were essentially neutrophils and macrophages [20,21,26,29,39,47], whereas CD4^+^ and CD8^+^ lymphocytes were reported elsewhere [26,29]. Increased infiltration of inflammatory cells following exposure for more than 3 months is also observed histologically [44], along with larger type II epithelial cells [16]. Specific observations linked dose-dependent lung inflammation to accumulation of cells in the terminal respiratory tissue [16], in particular neutrophils [39,45] and foamy multinucleated alveolar macrophages [45]. In addition to the cellular profile, a significant increase in total protein levels is also reported for exposure regimes exceeding 3 months [29,37,47,51]. The increase appeared to be dose dependent in one study [26]. The types of proteins increased are mainly proinflammatory markers such as TNF-α [27,28,71], IFN-γ [26,28], and IL-1β [28,71].

In vitro experiments with human or rodent primary cells or cell lines confirm the presence of high levels of proinflammatory proteins or mRNA after exposure to micrometric crystalline silica (cristobalite or quartz). In human and rodent epithelial cell lines, exposure to micrometric crystalline silica particles also significantly increased proinflammatory markers such as IL-1β and HMGB1 (in a surface-reactivity-dependent manner) [35], and IL-6 [72,73]. Other studies reported increased IL-8 levels [73,74,75], which were potentially directly correlated with increases in IL-1β [76], MCP-1 [77], and MIP-2 [70,74,77]. Only one article reported no significant increase in mRNA expression levels of IL-1α, IL-1β, and IL-6 in the human bronchial epithelial cell line 16HBE following incubation with 50 µg/mL silica [72]. Human and rodent macrophages exposed to cristobalite or quartz also significantly increased their production of proinflammatory markers including IL-1α [38], IL-1β [51,78], HMGB1 (in a surface-reactivity-dependent manner) [35], IL-6 [78], IL-8 (potentially directly correlated with the increase in IL-1β) [76], IL-13 [31], BAFF (B cell activator factor) [69], and IL-18 [51]. TNF-α production also increased [70,78], to a more significant extent with smaller (300 nm) particles [79].

The NF-κB (nuclear factor-kappa B) family of transcription factors is activated in response to a variety of stimuli and is known to be one of the multiple regulators of several genes encoding inflammatory proteins. TNF-family cytokines activate NF-κB signaling pathways. Magnone and colleagues [80] showed that abscisic acid (ABA) and lanC-like protein 2 (LANCL2) are key mediators in quartz-induced inflammation. These two mediators are induced in murine RAW264.7 macrophages and in rat alveolar macrophages. Autocrine ABA released after quartz exposure sequentially activates the plasma membrane receptor LANCL2 and NADPH oxidase, triggering Ca2+ influx resulting in NF-κB nuclear translocation and significant release of both TNF-α and PGE2. Macrophages silenced for LANCL2 or preincubated with a monoclonal antibody binding ABA show almost no NF-κB nuclear translocation, or TNF-α and PGE2 release, in response to quartz exposure [80]. Moreover, after micrometric crystalline silica exposure, NF-κB regulates inducible PGE2 synthase expression.

The COX-2/PGE-2 arachidonic acid metabolism pathway has also been investigated in primary human lung fibroblast cultures. Micrometric crystalline silica particles (100 µg/mL) induce activation of the COX-2 and PGE-2 pathways. Indeed, a significant increase in COX-2 gene and protein expression is observed alongside (dose-dependent) increased expression of the proteins mPGES and PGE2 [81]. Quartz particles also trigger activation of the cyclooxygenase–prostaglandin pathway in human and rat alveolar macrophages. Choi and colleagues [82] suggest that silica activates transcription of the human COX-2 gene through the induction of NF-kB activity. Moreover, eicosanoid levels measured after silica exposure reveal a significant increase in expression of PGE2 [83,84] and leukotriene B4 [84], but also C4, D4, and E4 expression [83], alongside a significant decrease in thromboxane B2 (TxB2) expression [83,84]. It should be noted that opposite results tend to be reported when surfactant is added with silica [84]. Following incubation with ascorbic acid-treated and untreated quartz, COX-2 and PGE-2 mRNA levels were increased in the murine macrophage cell line RAW264.7, and in rat alveolar macrophages derived from BALF compared to controls [85].

Furthermore, micrometric crystalline silica induces both acute and chronic inflammation in the specific immunodeficient animal model, NMRI mice. Indeed, intratracheal instillation of micrometric crystalline silica in these animals triggers a type 1 immune response, with significant expression of the IL-12 p40 subunit [29], along with a type 2 immune response, with significant increases in IL-4 and IL-13 expression [49] and an augmented IgG1/IgG2a ratio [29,49]. Interestingly, we found only one study reporting a link between inflammation and the animal’s sex. Thus, following oropharyngeal exposure of mice to 40 mg/kg silica, inflammatory cytokines (IFN-γ, TNF-α, IL-1β, IL-6, and IL-10) were increased to a greater extent in females compared to males at 7 days post exposure [52]. Following chronic exposure to low concentrations, 8 weeks after the last dose, males were found to be more susceptible to chronic silica-induced lung disorders, such as alveolitis, and showed greater dendritic cell presence compared to females [52].

- Nanometric Crystalline Silica and Inflammation

Following a single intratracheal installation of 5 mg/kg nanoquartz in rats, neutrophil infiltration was detected in BALF until 30 days. High total protein levels were also observed in BALF 24 h after a single 1 mg/kg dose of nanoquartz, and until 30 days after a single 5 mg/kg dose [46].

- Micrometric Amorphous Silica and Inflammation

As for crystalline microparticles, micrometric amorphous silica induces a significant increase in total cells in BALF from mice instilled with 100 mg/kg of silica [38]. No similar infiltration is observed in rats after instillation of 0.03 mg/kg [55]. In terms of differential cellularity, a significant increase in neutrophil numbers is reported [20,38,54]. In lung tissue sections, this increase is also observed with neutrophilic inflammation in alveoli in mice exposed intratracheally to 120 mg/kg of silica particles [63]. Other observations indicate that 3 days after the last instillation, silica could lead to bronchiolar degeneration or necrosis, with swelling of type II alveolar cells and accumulation of particle-laden alveolar macrophages [63]. In addition to this cellular profile, a significant increase in total protein levels is reported [56]. ELISA-based quantification reveals that the proteins increased mainly correspond to the proinflammatory markers IL-1α [38], IL-1β [38], and IL-6 [57].

In vitro studies confirm high levels of proinflammatory proteins or increased mRNA expression following exposure to micrometric amorphous silica, leading to IL-1α [38] and IL-1β [86] production by mouse alveolar macrophages, and the release of IL-6 and IL-8 by human pulmonary epithelial cell lines [73,87]. Moreover, amorphous particles more potently induce IL-6 than crystalline silica [73], and the enhanced potency is confirmed for both cytokines when comparing 500 nm particles to 50 nm particles in protocols applying the same total surface area [87].

Very little mention is made in the literature of the effects of micrometric amorphous silica on COX-2/PGE-2, and we found no data on effects on the NF-κB pathway. However, as for micrometric crystalline silica, micrometric amorphous silica can activate the COX-2/PGE-2 pathway. Increased COX-2 gene and protein expression were observed following exposure to a concentration of 10 µg/mL. These results lead to the conclusion that micrometric amorphous silica is more potent than crystalline silica [81].

- Nanometric Amorphous Silica and Inflammation

As described for micrometric crystalline and amorphous silica, in vivo experimental studies with nanometric amorphous silica confirm that it also induces a significant increase in total BALF cells [38,43,56,61,62]. Examination of the different cell types in BALF reveals a significant increase in neutrophil [38,43,56,57,62], eosinophil [54], and macrophage and lymphocyte [56] numbers. The neutrophil influx is size dependent (higher upon exposure to smaller particles) [43,54,55]. Histological analyses confirmed severe neutrophilic inflammation in lung tissue [61], localized specifically in alveoli [63] and BALT (bronchus-associated lymphoid tissue) [56]. The increased macrophage count was also histologically observable—with accumulation of particle-laden alveolar macrophages [63]. Other observations indicated that at 3 days after instillation, silica could lead to a slight thickening of the alveolar septum [56]. This thickening is associated with bronchiolar degeneration, necrosis, and alveolar type II cell swelling. According to Lee and colleagues [60], inhaled particles could also accumulate in peribronchiolar or perivascular BALT. Although only one study reported a significant increase in total protein levels [56], mRNA levels for the proinflammatory markers TNF-α, IL-1β, IL-6, IL-8, MCP-1, and MIP-2 were found to be significantly increased in BALF [61]. Increased protein expression was also detected for IL-1α [38], IL-1β [38,61], IL-6 [57,61], TNF-α, IL-8, MCP-1, and MIP-2 [61]. These results were all confirmed by in vitro experimental studies. Indeed, after exposure to nanometric amorphous silica, high expression of proinflammatory proteins or mRNA molecules is detected in human and rodent pulmonary epithelial cell lines. Thus, reports indicate significantly increased protein expression for a number of proinflammatory markers such as TNF-α [62,88], CXCL1 [62], IL-6 [62,88,89], IL-8 [88,89], MIP-1α [88], MIP-1β [88] and MIP-2 [90], and increased mRNA expression for IL-6 and IL-8 [87,91].

Interestingly, increased IL-6 and IL-8 expression appeared to be size dependent: a higher increase was detected with 10 nm particles compared to 50 nm particles in the human bronchial epithelial cell line BEAS-2B [92].

As for micrometric crystalline or amorphous silica, amorphous nanosilica induces the NF-κB pathway in human BEAS-2B cells and THP-1 cells (human leukemia monocytic cell), and murine RAW264.7 macrophages [66,87,92,93,94]. Only one in vivo study examined this pathway [65]; the authors reported no COX-2 effect at 24 h, 7 days, or 30 days after intratracheal instillation in rats (600 μg nanosilica).

Conclusion on acute and chronic inflammation:

Overall, acute or subacute inflammation is the most studied response to silica exposure. Whatever the size and crystallinity, all types of silica particles induce neutrophilic inflammation in BALF. Exposure to silica (except nanocrystalline silica, for which no data are available) consistently involved increased expression of inflammatory proteins, whatever the cell type (macrophage/monocyte, epithelial cell, or fibroblast) or animal model. The main proteins implicated are TNF-α, IL-1β, IL-6, and IL-8. In addition, the COX-2/PGE-2 pathway is activated upon exposure to micrometric silica whatever its crystallinity (no data for nanometric silica), and the NF-κB pathway is activated by exposure to microcrystalline silica and nanometric silica (no data for micrometric amorphous silica or nanoquartz).

### 4.2. Inflammasome

Inflammasomes are innate immune system complexes that constitute molecular receptors and sensors regulating the activation of caspase-1 protein and inducing inflammation (e.g., IL-1β secretion) in response to various stimuli (e.g., infectious agents, particles, chemical agents, or host-derived molecules).

- Micrometric Crystalline Silica and Inflammasome

Among the pathways regulating cytokine release induced by micrometric crystalline silica, some authors have highlighted the significant role played by NOD-like receptor pyrin domain-containing 3 (NLRP3) inflammasome formation in response to cellular damage [35]. Inflammasomes then trigger a significant increase in IL-1β expression alongside augmented caspase-1 activity. Early release of alarmins (IL-1α and IL-33, but not HMGB1) into the alveolar space is implicated in this process. Indeed, release of these molecules was shown to precede the expression of pro-IL-1β in cultured murine macrophages and neutrophil infiltration in the lungs of exposed mice [38]. Silica exposure induces a significant increase in expression of NLRP3, ASC (apoptosis-associated speck-like protein containing CARD) and caspase-1 mRNA [51,95], which is associated with higher caspase-1 activity in human THP-1 cells [35]. Activated caspase-1 can then cleave pro-IL-1β to produce IL-1β [51,96], triggering neutrophil recruitment [97]. In addition, in mouse alveolar macrophages, IL-1α induces significant pro-IL-1β production [38]. Finally, siRNA-mediated knockdown of NLRP3 reduced IL-1β production in mouse macrophage cultures (RAW264.7) and primary rat macrophages [86]. NF-κB activation and increased TNF-α and IL-1 production were also observed in rat BALF from 5 days after exposure to silica by inhalation; these effects persisted up to 116 days after exposure [98].

- Nanometric Crystalline Silica and Inflammasome: No data.
- Micrometric Amorphous Silica and Inflammasome

The literature is very limited when it comes to how micrometric amorphous silica affects the inflammasome. However, as for micrometric crystalline silica, exposure to micrometric amorphous silica leads to activation of the NLRP3 inflammasome. Indeed, some authors reported that particle-induced IL-1β release was reduced in RAW264.7 cultures (mouse macrophages) and in primary rat macrophages following siRNA-mediated NLRP3 knockdown [86].

- Nanometric Amorphous Silica and Inflammasome

As for both micrometric and amorphous crystalline silica forms, exposure to amorphous nanosilica activates the inflammasome. Indeed, murine alveolar macrophages, murine bone-marrow-derived macrophages and human THP-1 cells exposed to amorphous nanosilica all present increased levels of IL-1β as a result of NLRP3 inflammasome activation and increased caspase-1 activity [66,67,86,93]. Functional expression cloning identified the class B scavenger receptor SR-B1 as a receptor for amorphous and crystalline silica [99]. Silica was shown to bind to the extracellular α-helix of this protein in both mouse macrophages and human peripheral blood monocytes. By deleting the *SR-B1* gene and using monoclonal antibodies, this receptor was shown to be associated with canonical inflammasome activation [94,99]. Thus, SR-B1-mediated recognition of silica is associated with caspase-1-mediated inflammatory responses. As shown by Rabolli and colleagues [38] using micrometric crystalline silica, early release of IL-1α and IL-33 triggers pro-IL-1β and neutrophilic inflammation in nanosilica-treated murine macrophage cultures and in the lungs of exposed mice. Interestingly, reducing the surface silanol density of nanosilica by doping with titanium and aluminum could reduce NLRP3 inflammasome activation in human THP-1 cells and in bone-marrow-derived macrophages [59]. The reduction follows a dose-dependent pattern. Furthermore, high expression of IL-1β, thioredoxin-interacting protein (TXNIP) and NLRP3 inflammasome proteins are also associated with nanosilica exposure in an asthmatic mouse model (ovalbumin (OVA)-induced model) [100]. Similar results were reported by Marzaioli and colleagues [67]. In addition, in vitro and in vivo analyses revealed attenuation of inflammasome expression when amorphous nanosilica was coated with phosphonate or amino (-NH2) groups, but not with PEGylated nanosilica [67].

Conclusion on inflammasome:

Overall, all types of silica particles—whatever their size and crystallinity (except nanocrystalline silica, for which no data are available)—induce inflammasome activation, in particular through caspase-1 mediated by the NLRP3 complex. This activation results in increased IL-1β protein levels.

### 4.3. Acute and Chronic Fibrosis

Fibrosis is a dysfunctional wound repair process, characterized by a failure of tissue regeneration and excessive deposition of extracellular matrix (ECM) by fibroblasts. TGF-β1 is one of the most potent inducers of ECM production, including collagen fibers and other matrix proteins.

- Micrometric Crystalline Silica and Fibrosis

Acute fibrosis was observed in lung parenchyma and BALF after exposure of various animal models to microcrystalline silica. This type of silica significantly alters profibrotic markers, for example by increasing expression of cytokine CXCL10 [28] and TGF-β [37,39,50,101], or by enhancing PA (plasminogen activator) activity [30]. Immunohistochemistry analysis revealed a significant increase in 10E4 antigen levels accompanied by a decrease in SULF1 (sulfatase 1) protein levels [36]. This enzyme is implicated in heparan sulfate proteoglycan desulfatation and acts as a coreceptor for many heparin-binding growth factors and cytokines; it is also involved in cell signaling [36]. More recently, rats exposed to microcrystalline silica by intratracheal instillation were found to display pulmonary inflammation and fibrosis from 1 to 28 days after exposure. Numbers of fibroblasts and inflammatory cells decreased gradually from day 14, which is considered a crucial timepoint in silicosis progression [102]. In human epithelial cells, Perkins and colleagues [36] also suggest a potential role for SULF1 in silica-induced proliferative and fibrogenic signaling that could lead to epithelial hyperplasia, fibroblast recruitment, and deposition of ECM. Indeed, SULF1 overexpression leads to a significant increase in the expression of proliferative (CCND1, JUN, VEGFA, and BIRC3) and fibrogenic (COLIA1, PAI1, AVTA2, and collagen) genes after incubation with 150 × 10^6^ µm^2^/cm^2^ cristobalite. In a surface-reactivity-dependent manner [35], other fibrosis markers are also significantly increased, including FGF-2 [76] and bFGF [72,95]. In human fibroblast cells, exposure to 1 µg/mL Min-U-Sil significantly increases indicators of fibrosis, such as Masson’s trichrome staining, α-SMA expression, and matrix contraction [103]. Moreover, cathepsin K, controlled by TGF-β1, is overexpressed in silica-exposed fibroblast cultures [50]. In human THP-1 cells, exposure to silica induces a significant surface-reactivity-dependent increase in bFGF [35] and TGF-β expression [104]. As collagen deposition is one of the main mechanisms underlying fibrosis, some studies assessed collagen markers. Several groups reported a significant increase in hydroxyproline content [24,49,50] and expression of the MARCO (macrophage receptor with collagenous structure) receptor [44]. In vitro, Bodo and colleagues [72] detected significant collagen and fibronectin production in human 16HBE cells. Using histological methods, other authors observed increased fibroblast proliferation and collagen deposition in mice 4 weeks after intratracheal instillation of 40 mg/kg of silica [24]. More recently, it was observed that overexpression of the multifunctional miR-138 alleviates silica-induced pulmonary fibrosis in mice [105].

Chronic fibrosis was also investigated in lung parenchyma and BALF after exposure to microcrystalline silica. As shown previously with acute fibrosis, this type of silica induces a significant increase in expression of various profibrotic markers, such as TGF-β1 [37,39,50], CXCL10 [28], IL-10 [27,37,39], and PDGF-B [37,39]. Immunohistochemistry revealed contrasting effects of chronic versus acute exposure, with a significant increase in SULF1 protein levels and decreased 10E4 antigen levels following chronic exposure [36]. Some studies assessed collagen markers and reported significantly increased hydroxyproline content [23,24,25,27,37,39,44,50], along with soluble collagen [37] and type I collagen [27]. Using histological methods, lung tissue thickening [45] was linked to progressive and time-dependent fibrosis [47], but also to the emergence of fibrotic nodules 120 days after mice were intratracheally exposed to 100 mg/kg of silica particles [29].

- Nanometric Crystalline Silica and Fibrosis: No data.
- Micrometric Amorphous Silica and Fibrosis: No data.
- Nanometric Amorphous Silica and Fibrosis

Wang and colleagues [64] observed a significant increase in histological score for Masson’s trichrome staining in samples from mice exposed to SiNP-100. This staining colocalized with TGF-β1 at the cell membrane. Moreover, in human epithelial cells, immunofluorescence analysis revealed significant colocalization of 100 nm (but not 10 nm) SiNP and TGF-β1 at the membrane of human epithelial cells (A549) [64]. These cells enhanced and prolonged TGF-β1 activity in their corona, which induces fibrogenic pathways; free TGF-β1 does not have this effect [64]. Investigation of the pathways regulating amorphous nanosilica-induced TGF-β1 release revealed Smad2 to be implicated in the fibrogenic pathway. Indeed, incubation with 100 nm (but not 10 nm) SiNP led to a significant and prolonged increase in Smad2 phosphorylation [64]. In addition, one study linked exposure to amorphous silica NPs and alveolar remodeling in a sex-dependent manner. Thus, in rats exposed intratracheally to 2 mg/kg of 20 nm SiNP, females showed a significant increase in expression of caveolin-1, a regulator of ECM (including collagen) deposition, and MMP-9 compared to males [62].

For chronic fibrosis, Sutunkova and colleagues [43] reported a significant dose-dependent increase in the fibrotic marker hydroxyproline after 90 days’ exposure in rats (“nose-only” inhalation exposure; dose range 2.6 to 10.6 mg/m^3^).

Conclusion on acute or chronic fibrosis:

No data are available for micrometric amorphous silica or nanoquartz. In studies involving exposure of rodent models to microcrystalline silica or nanosilica, acute or subacute fibrosis was generally studied. Both types of particles induce a significant increase in profibrotic markers in lung parenchyma and in BAL. Thus, TGF-β1 is the main marker enhanced, and the effect is dose dependent with nanosilica.

### 4.4. Cell Death

Cell death is a biological event that is required for the natural process of cell regeneration, but it may also be triggered by factors such as disease, aggression, or injury. Morphologically, cell death can take various forms, including apoptosis or autophagy. The destruction of cells, known as cytolysis, can be detected based on the release of nonspecific intracellular cytotoxicity markers (e.g., lactate dehydrogenase (LDH) or albumin protein).

#### 4.4.1. Cytotoxicity

- Micrometric crystalline silica and cytotoxicity

LDH seems to be an important factor in the reaction to silica exposure. Indeed, LDH activity is reported to be significantly increased in a large number of studies [18,19,20,21,27,29,30,37,41,45,47,51,69], with the increase being potentially dose dependent [26]. Cellular cytolysis has also been reported based on serum albumin concentrations [98]. In vitro experimental studies in a human epithelial cell line demonstrate that exposure to 40 µg/cm^2^ of quartz particles triggered increased LDH release [73]. However, no significant differences were detected when using rat epithelial cells, even with high quartz concentrations (500 µg/mL, equivalent to 130 µg/cm^2^) [106]. In a non-pulmonary macrophage cell line exposed to at least 50 µg/cm^2^ silica, LDH activity increased significantly [107] in a dose-dependent manner [108].

- Nanometric crystalline silica and cytotoxicity

The unique study identified using nanoquartz showed that these particles induce cytotoxicity after a single intratracheal installation in rats, as indicated by high levels of LDH in BALF, detected only after the 5 mg/kg single dose, and until 3 months post exposure [46].

- Micrometric amorphous silica and cytotoxicity

In contrast to the effect of crystalline silica, after exposure to amorphous silica, LDH release seems to be more transient. Indeed, in rats exposed to 10 mg/m^3^ of precipitated silica or 50 mg/m^3^ colloidal silica, a significant increase in LDH release was detected at early stages but not 10 days after the initial exposure [20]. Moreover, no significant difference in LDH release was found in rats exposed to 1.2 mg/kg silica particles (200 nm) either intratracheally [55] or by nose-only inhalation [109]. However, when using a human pulmonary epithelial cell line, incubation with 300 and 500 nm silica particles triggered increased LDH release [73,87].

- Nanometric amorphous silica and cytotoxicity

In vivo, LDH release in rats following intratracheal exposure to 30 µg (1.2 mg/kg) of amorphous silica nanoparticles was not significantly different to levels in controls [55]. In contrast, when using a human pulmonary epithelial cell line exposed to nanosilica, LDH activity was significantly increased [87,89,92] in a dose-dependent manner [90,110]. At lower nanosilica concentrations (5 µg/cm^2^), no significant difference in LDH release was demonstrated in human A549 adenocarcinoma alveolar basal epithelial cells or THP-1 cells [88]. Finally, based on LDH release, nanosilica significantly increased cytotoxicity in non-pulmonary mouse RAW264.7 macrophages and human THP-1 macrophages at high doses (from 50 µg/mL) [104,111], and also in human H441 lung adenocarcinoma cells (up to 600 µg/mL NexSil20 or Ludox TM-40) [89]. The effect was time- and dose dependent. In [89], no significant effect on cell viability (MTS assay) was demonstrated with either type of nanosilica at 300 μg/mL. In contrast, in Chinese hamster fibroblast V79 cells, Guichard and colleagues [112] observed a significant and size-dependent (no effects for sizes greater than 20 nm) decrease in cell viability (WST-1 assay) with pyrogenic, precipitated, or colloidal amorphous silica particles.

Conclusion on cytotoxicity:

Overall, whatever the size and crystallinity, silica has a dose-dependent cytotoxic effect, revealed by LDH release in multiple studies.

#### 4.4.2. Apoptosis

Apoptosis is a process of programmed cell death whereby a programmed sequence of events allows the elimination of cells without any release of internal constituents into the surrounding area. Apoptosis plays a crucial role during early fetal development, childhood, and adulthood in the elimination of old, unnecessary, and unhealthy cells.

- Micrometric crystalline silica and apoptosis

Upon exploration of the pathways regulating crystalline-silica-induced cytotoxicity, a number of authors highlighted the crucial role played by apoptosis. Indeed, a significant increase in caspase-3 cleavage is reported in mice following exposure to 1 mg DQ-12 [28]. Calculation of the cleaved/uncleaved caspase-3 ratio also indicated activation of the apoptotic pathway in rodent alveolar and non-pulmonary macrophages exposed to 50 µg/cm^2^ silica [28,113]. Similarly, in a human epithelial cell line, incubation with 60 µg/cm^2^ quartz led to phosphorylation of SFK, p38 [75], and extracellular signal-regulated kinase (ERK)1/2 [74,75,114]. Moreover, ERK activation was linked to a significant increase in mRNA expression, corresponding to activation of c-fos and JUN family members in rodent cells following exposure to 10 µg/cm^2^ quartz [114]. Propidium iodide staining revealed a significant increase in macrophage death [113], which was time- and concentration dependent [115].

For this type of silica, in mice exposed to 1 mg DQ-12, and in non-pulmonary macrophages incubated with 250 µg/mL quartz, other cell death pathways have been implicated [28], such as pyroptosis, with a significant increase in gasdermin D cleavage, and necrosis, assessed based on MLKL (mixed lineage kinase domain-like pseudokinase) phosphorylation. Autophagy, as estimated based on Fas and p62 expression levels, was also highly increased in histiocytes (in granulomas) [42]. Moreover, in mouse alveolar macrophages (MH-S cells) incubated with 50 µg/cm^2^ crystalline silica, Joshi and colleagues [116] showed that this exposure can result in either apoptosis or necrosis, with either form of cell death occurring in a well-defined but temporally variable order.

- Nanometric crystalline silica and apoptosis: no data
- Micrometric amorphous silica and apoptosis

An increased apoptotic index (TUNEL assay) is reported in lung parenchyma [56] and in mouse alveolar macrophages (MH-S cells) following exposure to 50 µg/cm^2^ of 3 µm silica (no effects for 1 µm silica) [113]. Propidium iodide staining and caspase-3 cleavage demonstrated a significant increase in cell death rates.

- Nanometric amorphous silica and apoptosis

The essential role played by apoptosis, and specifically the potential induction of ER-stress was highlighted in some articles [89]. However, viability was reported to be relatively little affected after exposure to 50 nm nanosilica, with insignificant toxicity (based on propidium iodide staining) in human epithelial cells (BEAS-2B cells) at 200 µg/mL [91]. In contrast, incubation with 10 nm Si at 25 μg/mL (and to a lower extent with 50 nm Si at 100 μg/mL) induced p38 phosphorylation in human BEAS-2B cells at timescales from 1 to 8 h after exposure [92]. Other authors used a caspase-3 assay to demonstrate activation of the apoptotic pathway in Chinese hamster fibroblast V79 cells exposed to at least 50 µg/cm^2^ (negligible effects for particle sizes greater than 20 nm) [112].

Conclusion on apoptosis:

Overall, whatever the size and the crystalline structure (no data available for nanoquartz), silica triggers apoptosis. Furthermore, it can be clearly seen that apoptosis is mainly mediated via the intrinsic and/or mitochondrial pathway (caspase-dependent pathway) in a size- and dose-dependent manner.

#### 4.4.3. Autophagy

Autophagy is a cellular process that allows the degradation of cytoplasmic components such as damaged or unwanted proteins or organelles after their capture in a double lipid membrane—the autophagosome. Degradation occurs after fusion of the autophagosome with a lysosome to form the autolysosome.

- Micrometric crystalline silica and autophagy

Exposure to crystalline silica disrupts normal autophagic degradation in alveolar macrophages leading to autophagosome accumulation and lysosome disruption. Indeed, crystalline silica regulates autophagic activity via the PI3K/Akt/mTOR (phosphatidylinositol 3 kinase/α-serine-threonine protein kinase/mammalian target of rapamycin) signaling pathway. In addition, recent studies indicate that autophagy reduces crystalline-silica-induced apoptosis of alveolar macrophages when mTOR is inhibited by rapamycin [117]. However, the authors did not specify the mean size and concentration of silica applied. Autophagy also reduces TNF-α and TGF-β (transforming growth factor-β) expression in silica-exposed alveolar macrophages [101]. Moreover, mitophagy contributes to this process when mitochondrial reactive oxygen species (mtROS) released by alveolar macrophages leads to dysregulation of mitochondrial function [118]. Recently, the cGAS-STING (stimulator of interferon genes) pathway, which is crucial for immune defense, has been explored. STING activation triggers multiple signaling cascades leading to activation of the NF-κB pathway and autophagy. One recent study linked inflammation triggered by exposure to micrometric crystalline silica with a role for the STING protein [28]. In this study, non-pulmonary macrophages were incubated with 250 µg/mL quartz, leading to an increase in ds-DNA alongside overexpression of the STING and cGAS genes. In parallel, quartz disrupts lysosomes, resulting in excessive autophagosome formation; the backlog in autophagic degradation in alveolar macrophages then triggers an apoptotic mechanism. Another recent study showed that exposure to micrometric crystalline silica also induces lysosome damage and autophagosome accumulation in lung tissues in wild-type mice, and reduced autophagosome formation in Gas-6^−/−^ and Mer^−/−^ mice [119]. The growth arrest protein Gas-6 binds the TAM (Tyro3, Axl, and Mertk) receptor, a member of a family of tyrosine kinase receptors. Gas-6 depletion suppresses mitophagy by activating mTOR signaling to decrease autophagosome formation. Significant increases in the LC3B-II/LC3B-I ratio, ATG5 (only after 7 days), Beclin-1, Mer and P62 protein, and/or mRNA expression were observed in WT mice [119]. In addition, expression of p-mTOR and LAMP1 was significantly decreased after exposure to micrometric crystalline silica [119].

- Nanometric crystalline silica and autophagy: no data
- Micrometric amorphous silica and autophagy: no data
- Nanometric amorphous silica and autophagy

As for micrometric crystalline silica, amorphous nanosilica induces autophagy mainly via oxidative stress and the PARP (poly (ADP-ribose) polymerase)/TRPM2 (transient receptor potential melastatin-2) signaling pathways. Indeed, after inhibition of reactive oxygen species (ROS) generation, the PARP and TRPM2 channels suppress nanosilica-induced lysosome impairment and autophagy dysfunction in human BEAS-2B cells and in mouse lungs [68]. PARP and TRPM2 are known to promote autophagy via the mTOR and c-JNK (c-Jun N-terminal kinase) signaling pathways, respectively [120]. Lysosome acidification is a prerequisite for particle-induced lysosome membrane permeabilization, and the subsequent leakage of lysosome cathepsins is a primary regulator of NLRP3 inflammasome activity and HMGB1 protein release [51]. Furthermore, the induction of autophagy correlates with the extent of cytotoxicity, suggesting that nanosilica exposure causes irreversible cellular damage, ultimately leading to autophagic cell death [104].

Conclusion on autophagy:

Overall, microcrystalline or nanometric amorphous silica induce autophagy mainly via oxidative-stress-mediated upregulation of autophagy-related genes and differential regulation of the PI3K/Akt/mTOR signaling pathway. Induction of autophagy correlates with the extent of cytotoxicity, suggesting that both types of particles cause irreversible cellular damage leading to autophagic cell death. In support of this hypothesis, lysosomal and autophagic dysfunction is a known mechanism of microcrystalline silica toxicity.

### 4.5. Oxidative Stress

Oxidative stress results from an imbalance between the production of oxidants (e.g., ROS or free radicals) and antioxidant defenses; it can lead to cell and tissue damage, and is also involved in the aging process.

- Micrometric Crystalline Silica and Oxidative Stress

The relationship between silica exposure, oxidative stress, and pulmonary damage has been investigated in numerous studies.

In acute studies, ROS release seems to be an important reaction to exposure to silica. Indeed, some studies reported a significant increase in the ·OH hydroxyl radical [32,41], specifically localized in phagocytes [21]. Some studies suggest that ROS release is size dependent (only triggered by exposure to particles smaller than 300 nm) [28,79]. In vitro studies in non-pulmonary macrophages demonstrate that mitochondrial ROS release is triggered by silica exposure, and immunofluorescence assays staining for MitoSOX confirmed that ROS originate in mitochondria [28]. Other oxidant markers were also increased, such as the NOx concentration [22] and lipid peroxidation levels [22]. In response to ROS release, significant increases in the enzyme heme-oxygenase-1 (HO-1) are detected specifically in alveolar macrophages [121]. Antioxidant markers, such as glutathione peroxidase [17] and SOD activity [22], also increase significantly [22], in particular the mitochondrial Mn-SOD (at both mRNA and protein levels) [16,17].

In lungs from rats exposed to micrometric crystalline silica by inhalation (15 mg/m^3^, 6 h/day) for 20, 40, or 60 days, a relationship was observed between increasing ROS production levels and the time post exposure [98]. Lung NO (nitric oxide) production was also significantly increased in silica-exposed rats, with further increases during the post exposure period [98]. NO and ROS production both increase in alveolar macrophages after exposure of lungs to micrometric crystalline silica, with iNOS (inducible nitric oxide synthase) protein expression displaying a dose-effect pattern in response to silica exposure [98]. In addition to iNOS expression, oxidative stress elicited by crystalline silica also leads to increased expression of antioxidant enzymes, such as manganese superoxide dismutase (Mn-SOD) and glutathione peroxidase [13]. In human bronchial epithelial cells, exposure to silica is linked to significantly increased intracellular H_2_O_2_ [122]. Superoxide anions (O_2_^•−^), and H_2_O_2_ are generated in silica-treated medium by a process requiring iron [122]. Furthermore, generation of oxidants in cells following exposure to crystalline silica microparticles and silica activation leads to the activation of multiple cell signaling pathways. These pathways include MAPK/ERK kinase (MEK), and ERK phosphorylation, and increased expression of inflammatory cytokines [13]. In rats chronically exposed by inhalation (15 mg/m^3^ silica, 6 h/day, 5 days/week for up to 4 months), generation of ROS and NO, in particular, is temporally and anatomically associated with the development of lung damage, alongside inflammation, granulomas, and fibrosis [71]. Based on these observations, a central role has been suggested for NO in the initiation of silicosis [71]. In an iNOS-KO mouse model, Zeidler and colleagues [70] showed that activation of alveolar macrophages was reduced in iNOS-KO mice compared to WT mice. Moreover, lung hydroxyproline levels were significantly lower in iNOS-KO versus WT mice, suggesting that iNOS-derived NO also contributes to the pathogenesis of silica-induced lung disease in WT animals [70].

- Nanometric Crystalline Silica and Oxidative Stress: No data.
- Micrometric Amorphous Silica and Oxidative Stress

Brown and colleagues [55] assessed oxidative stress based on Nrf2 staining and found no particular induction in rats exposed intratracheally to 1.2 mg/kg of 0.2 µm silica particles [55]. However, in vitro studies with a mouse lung epithelial cell line (FE1 cells) indicate that incubation with at least 12.5 µg/mL of micrometric amorphous particles had a higher potential to induce ROS and intracellular glutathione (GSH) than exposure to nanometric particles [123]. Similarly, when mouse alveolar macrophages (MH-S cells) were incubated with 20 µg/cm^3^, NOX2-generated ROS were specifically detected in the cytoplasm in parallel to an increase in phagosomal ROS. During late apoptosis, these species were also associated with mitochondrial ROS production [124].

- Nanometric Amorphous Silica and Oxidative Stress

In vitro studies in human and rodent lung epithelial cells indicate that ROS release appears to represent an important cellular response to silica exposure. Indeed, some articles indicated that exposure to nanosilica triggers ROS release in a mouse lung epithelial cell line (FE1 cells) [123]. ROS release was shown to be time dependent in BEAS-2B human lung epithelial cells [91], and dose dependent in human A549 adenocarcinoma alveolar basal epithelial cells [110]. In parallel to ROS release, HO-1 mRNA and protein levels were significantly increased after exposure to 50 nm silica particles at 50 µg/mL [91], with induction shown to involve the Nrf-2-ERK MAP kinase signaling pathway in human BEAS-2B cells [125]. Other oxidative stress markers were also perturbed in a dose-dependent manner in human epithelial cells: lipid peroxidation was significantly increased, whereas intracellular GSH was slightly depleted [110,126]. However, oxidative stress is only detected at high doses. Indeed, human epithelial cell lines exposed to low concentrations (10 µg/mL) of nanosilica display no significant differences in ROS formation, intracellular GSH, or SOD activity [127]. Among markers of oxidative stress, altered cell membrane integrity was also detected, with a decrease in phospholipids determined by metabolic analysis [126]. These effects may be species specific, however, as Guichard and colleagues [112] observed no significant difference in terms of ROS formation following exposure of a Chinese hamster fibroblast cell line to nanosilica [112].

Conclusion on oxidative stress:

Overall, all types of silica particles, with the exception of nanocrystalline silica (for which no data is available), induce a considerable degree of oxidative stress with disruption of the oxidant/antioxidant balance in a dose-dependent manner. The pathways triggered generally lead to NO and/or ROS formation (e.g., intracellular ·OH, O_2_^•−^ or H_2_O_2_) rather than depletion of antioxidant capacity (e.g., GSH).

### 4.6. Alveolar Macrophage Phenotype and Phagocytic Activity

- Micrometric Crystalline Silica and Macrophages

Zhao and colleagues [128] showed that crystalline silica particles could disrupt alveolar macrophage polarization of in mice [128]. Indeed, inhalation of α-quartz by rodents induces (arginase-1 positive) M2 anti-inflammatory alveolar macrophage phenotype polarization. M2 macrophages are the archetypical phagocytic subtype, displaying a strong potential to take up mineral particles without triggering inflammation. M2 subtypes owe their phagocytic capacity to the expression of abundant levels of scavenger receptors for quartz; they also appear to be relatively insensitive to inflammatory stimuli, as suggested by their low-level expression of iNOS. However, accumulation of quartz particles in M2 macrophages leads to macrophage overload, when the cells can no longer encapsulate any further quartz particles. As a consequence, free quartz particles distributed in interstitial lung tissue also come into contact with activated M1 inflammatory macrophages (iNOS-positive), triggering secretion of inflammatory cytokines. These silica-laden M1 macrophages initiate the formation of lung granuloma, where further silica particles are sequestered. In addition to overload, the capacity of M2 macrophages to clear particles is perturbed by lipopolysaccharides, which cause them to secrete IL-1β in response to α-quartz exposure. This IL-1β in turn stimulates M1 macrophages and dendritic cells to produce TNF-α and IFN-β, respectively [128], further exacerbating the localized inflammation.

Phagocytosis is a critical mechanism through which innate immune cells eliminate microbes, necrotic or apoptotic cells, and mineral particles, such as crystalline silica. Expression levels for various markers of activated phagocytosis have been reported to be altered after exposure to microcrystalline silica: significant increase in lysosomal enzymes NAG (*N*-acetyl glucosaminidase) [20,23] and glucuronidase [23], heightened levels of extracellular cathepsins (L, B, V) and intracellular cathepsin B [51] and cathepsin K [50], increased free cytosolic Ca^2+^ concentration and plasma membrane potential in bovine macrophages exposed to microcrystalline silica, and significantly decreased pHi [129]. However, phagocytosis could also be impaired by microcrystalline silica. Indeed, scanning electron micrograph (SEM) observations of pulmonary macrophages showed a deficit of phagocytic function in rats exposed to repeated 100 mg/m^3^ doses of Min-U-Sil through an aerosol chamber [19].

- Nanometric Crystalline Silica and Macrophages: No data.
- Micrometric Amorphous Silica and Macrophages

The phenotypic profiles of alveolar macrophages exposed to micrometric amorphous silica are not reported in the literature. However, as with microcrystalline silica, phagocytosis of micrometric amorphous silica particles seems to be activated, as indicated by significant increases in lysosomal enzymes NAG (*N*-acetyl glucosaminidase) in rat macrophages [20], free cytosolic Ca^2+^ concentration [129] and plasma membrane potential [129], and a significant decrease in pHi observed following exposure, in bovine macrophages for these three last ones [129]. Endolysosomal leakage is demonstrated in mouse alveolar macrophages (MH-S cells) exposed to 50 µg/cm^2^ of 3 µm amorphous silica (no effects for 1 µm amorphous silica) [113]. When NOX activity is inhibited in the same cell line, delayed phagolysosomal leakage occurred after exposure to 20 µg/cm^3^ of amorphous silica particles [124].

- Nanometric Amorphous Silica and Macrophages

Inhaled nanosilica are phagocytosed by alveolar macrophages [60]. Surprisingly, we only found one study that analyzed how nanosilica affects macrophage polarization [130]. The results indicated that increased nanosilica uptake in human THP-1 cell lines is associated with M2 polarization [130]. In corroboration of this conclusion, various markers of activated phagocytosis are detected after exposure to nanosilica. Indeed, in mouse pulmonary epithelial cells, incubation with nanosilica (12 nm) significantly modifies the expression of genes implicated in lysosomal functions, but also the internalization of the nanosilica itself and lysosomal rearrangements in the cytoplasm [123]. In addition, Sanchez and colleagues [131] reported a significant increase in basal calcium concentration [Ca^2+^] in human and rodent epithelial cell lines [131].

Conclusion on alveolar macrophage phenotype and phagocytic activity:

The main cell types used to study immune responses to silica were innate immune cells such as monocytes and macrophages (but also epithelial cells for nanosilica). No analysis of phenotypic profiles of alveolar macrophages exposed to micrometric amorphous silica or nanoquartz was found in the literature. Furthermore, the data on phagocytic responses are very limited for amorphous silica, whatever its size. Therefore, no firm conclusions can be drawn for these two types of particles. However, with microcrystalline silica or nanosilica, exposure is mainly linked to M2 polarization. Overall, microcrystalline silica of any size can be eliminated by phagocytosis, but it may also impair phagocytosis, and consequently disrupt the elimination of microbes, necrotic or apoptotic cells, and other mineral particles. Microcrystalline silica-laden M1 macrophages initiate lung granuloma formation. Although nanometric amorphous silica is mainly internalized by macrophages, and associated with M2-polarization, epithelial cells may also be involved. Currently, no data are available on a potential nanosilica-linked impairment of alveolar macrophage phagocytic processes.

### 4.7. Epithelial–Mesenchymal Transition

Epithelial to mesenchymal transition (EMT) is the process whereby epithelial cells, which cover the internal and external surface of the organism, are transformed into mesenchymal cells. EMT is the key step in lung fibrogenesis, and TGF-β1 is a potent mediator of this process.

- Micrometric Crystalline Silica and EMT

HMGB-1 is a nuclear protein that interacts with nucleosomes, transcription factors, and histones in order to organize DNA and regulate transcription. Recently, Ma and colleagues [132] showed that, in BALF from mice intratracheally instilled with crystalline silica, HMGB-1 is involved in macrophage and neutrophil accumulation, and in modulating IL-6 and TNF-α expression levels [132]. HMGB-1 seems to be implicated in fibronectin and collagen-1 expression, suggesting that HMGB-1-mediated EMT contributes to the development of silicosis on day 28 and 84 after microcrystalline silica administration [132]. Results from in vitro studies indicate that incubation of human epithelial cells with 25 µg/cm^2^ of microcrystalline silica leads to a significant increase in the EMT-related markers MMP-2 (matrix metalloproteinase), MMP-9, Col-1, and Col-3. Western blot analysis also revealed higher expression of α-SMA (smooth muscle actin) and vimentin (mesenchymal marker), along with weaker expression of E-cadherin and ZO-1 (epithelial marker) [133]. Moreover, Hu and colleagues [134] suggest that microcrystalline silica-induced EMT is mediated by Snail, a transcription factor that downregulates E-cadherin and upregulates vimentin expression [134]. More recently, in human A549 cells exposed to micrometric crystalline silica, expression of α-SMA and vimentin were found to be significantly increased, whereas E-cadherin was significantly decreased after transfection with miR-138 [105].

- Nanometric Crystalline Silica and EMT: No data.
- Micrometric Amorphous Silica and EMT

Recently, contrary to microcrystalline silica, following exposure to micrometric amorphous silica, reduced α-SMA protein activity was observed in human A549 cells. This reduction was the result of inhibition of silica-induced EMT, regulated by ZEB2. Furthermore, this delayed EMT was associated with upregulation of miR-138 [105].

- Nanometric Amorphous Silica and EMT

One study with nanosilica (100 nm SiNP) demonstrated possible induction of EMT in mice after intratracheal instillation [64]. Thus, the authors showed 100 nm SiNP in combination with TGF-β1to promote EMT in human A549 cells after incubation. The effect strictly required TGF-β1, as it was significantly reduced upon inclusion of a TGF-β1 receptor blocker (SB431542) [64]. Similarly, when human epithelial cells are incubated with 100 nm SiNP and TGF-β1, EMT is triggered. This effect is associated with a higher expression of vimentin (mesenchymal marker) and weaker E-cadherin (epithelial marker) expression. No effect was observed with 10 nm particles, suggesting that the effect is size dependent [64]. TGF-β1 was also increased in THP-1 cells exposed to amorphous nanosilica [104].

Conclusion on epithelial to mesenchymal transition:

Overall, despite the limited data available, it appears that EMT is mainly induced by microcrystalline silica and nanometric amorphous silica, with a possible size-dependent effect. EMT could lead to lung fibrogenesis as a result of TGF-β1 expression.

### 4.8. Granulomas (or Silicotic Nodules)

The hallmark of pulmonary silicosis is the formation of granulomas, also known as silicotic nodules: well-demarcated, rounded fibrotic lesions that tend to concentrate in the upper lung lobes.

- Micrometric Crystalline Silica and Granuloma

In addition to fibrosis, silicotic nodules are often observed at chronic stages when silica is retained in the lungs [18,27,35,39]. Indeed, aggregated/agglomerated silica particles are found in granuloma centers [24,42]. Furthermore, some studies reported emphysema-like effects, with a significant increase in neutrophil elastase levels [33], along with increased insoluble elastin [24].

- Nanometric Crystalline Silica and Granuloma: No data.
- Micrometric Amorphous Silica and Granuloma: No data.
- Nanometric Amorphous Silica and Granuloma

According to Lee and Kelly [60], in rats exposed in chambers to 50 to 150 mg/m^3^ of colloidal amorphous silica, granulomas initially form in a dose-dependent manner in alveoli. Particle-laden alveolar macrophages are observed alongside epithelioid cell proliferation [60]. This type of lesion can appear after just 1 week in mice exposed intratracheally to at least 1 mg/kg nanosilica particles [61]. However, in contrast to micrometric crystalline silica, collagen deposition is minimal in granulomas induced following exposure to nanosilica [60].

Conclusion on granulomas:

Despite the limited data available (no data for micrometric amorphous silica or nanoquartz), silicotic nodules are mainly induced by microcrystalline silica, but can also be triggered by nanometric amorphous silica. In comparison with microcrystalline silica, collagen deposition is minimal in silicotic nodules induced following exposure to nanosilica.

### 4.9. Muco-Ciliary Clearance and Other Effects

Muco-ciliary clearance is one the processes by which the lungs clean themselves of dust and particles that enter with inhaled air. A constant beating of the cilia entrains a conveyor belt of mucus from the bottom of the lungs to the top of the trachea.

- Micrometric Crystalline Silica and Muco-Ciliary Clearance

Microcrystalline silica could affect muco-ciliary clearance. Indeed, Yu and colleagues [48] reported a perturbed muco-ciliary structure and altered MUC5B production in mice following intratracheal exposure to a dose of 100 mg/kg [48]. Moreover, intratracheal instillation of 50 mg/kg silica in rats significantly increased the albumin concentration in BALF, suggesting an impact on the capillary–epithelial barrier [40]. Finally, changes in lung metabolism were observed in rats intratracheally exposed to 20 mg Min-U-Sil, with a significant increase in lung microsomal concentrations of proteins linked to CYP4501A1- and 2B1-mediated reactions [32].

- Nanometric Crystalline Silica and Muco-Ciliary Clearance: No data.
- Micrometric Amorphous Silica and Muco-Ciliary Clearance: No data.
- Nanometric Amorphous Silica and Muco-Ciliary Clearance

In human and rodent epithelial lung cell lines, compared to control conditions, incubation with 300 µg/mL of 10 nm colloidal silica particles led to a decrease in ciliary beat frequency through a significant inhibition of the TRV4 cation channel and a TRPV4-independent increase in basal [Ca^2+^] [131]. In vitro, porcine pulmonary surfactant extract incubated with at least 250 µg/mL of (50 nm) nanosilica displayed altered surfactant parameters. Indeed, the compression isotherm (a parameter simulating the physiological conditions in alveoli during breathing) and the foaming ability (a key indicator of the interface properties of pulmonary surfactant) were significantly perturbed [135].

Conclusion on muco-ciliary clearance and other effects:

Despite the limited data available (no data for micrometric amorphous silica or nanoquartz), in vivo exposure to very high doses of microcrystalline silica leads to disrupted muco-ciliary clearance in rodents. However, it should be noted that no data were available at realistic doses. In vitro, meanwhile, exposure to nanosilica leads to a decrease in ciliary beat frequency and altered pulmonary surfactant properties.

## 5. Discussion

### 5.1. Relevance of Doses Used

The majority of the experimental studies on animals reviewed here involved high—sometimes unrealistic—doses. Extrapolation to real human exposure levels in the workplace reveals maximal atmospheric concentrations ranging from a few mg/m^3^ to a few tens of mg/m^3^ when the occupational exposure limit values for most countries are significantly exceeded. Indeed, the exposure limit value for quartz recommended in the United States (NIOSH-REL value) is 0.05 mg/m^3^, and in France it is 0.1 mg/m^3^; for amorphous silica (nonspecific dusts) it is 6 or 4 mg/m^3^, respectively. These values should be kept in mind when considering data from whole-body inhalation studies, where the atmospheric concentrations used sometimes exceed 150 mg/m^3^ (https://www.osha.gov/chemicaldata/613 (accessed on 7 June 2022) and https://www.osha.gov/chemicaldata/278 (accessed on 7 June 2022)). With intratracheal or pharyngeal instillation, considering the regulatory daily occupational thresholds in most Western countries (examples above), realistic equivalent cumulative doses should be about 0.5 µg per mouse per week for quartz, and 50 µg per mouse per week for amorphous silica [136]. Similarly, for in vitro studies, if a silica mass concentration of 5 mg/m^3^ is applied, the realistic cumulative daily dose would be around 0.2 µg/cm^2^ according to the equation: Daily dose deposited on alveolar surface = (occupational exposure limit (OEL) (5 µg/L = 5 mg/m^3^) × percentage of deposited fraction of emitted aerosol (50% for NP with a mean aerodynamic diameter of 20 nm) × mean minute ventilation (22.5 L/min = tidal volume of 1.5 L × respiratory frequency of 15/min) × duration (480 min, 8 h per day))/alveolar surface (1,500,000 cm^2^ = 150 m^2^) [136]. However, in the studies used for this review, very high doses were used in most studies: ranging from 6.2 to 150 mg/m^3^ in whole-body inhalation studies, 2.6 to 27 mg/m^3^ in “nose-only” inhalation studies, and 0.02 to 640 mg/kg in intratracheal instillation studies. The use of such doses presents certain advantages in toxicology when seeking to assess risks, but cannot be considered to mimic actual work situations. Therefore, the biological, cellular, and tissue effects observed following exposure to such high doses are difficult to extrapolate to realistic working conditions. Consequently, when considering the data presented, it is essential to take into account the relevance of the dose applied. Interestingly, Di Cristo and colleagues designed a human-relevant 3D in vitro platform for simulation of workplace exposure to nanoparticles. Based on efficiency of pulmonary clearance of inhaled silica nanoparticles compared to recent in vivo data, this in vitro platform could compare tested doses to occupational exposure limit values [137].

### 5.2. Silica Physicochemical Characteristics

The in vitro and in vivo experimental studies reviewed here indicated that silica, whatever its crystallinity, shape, or size, can affect lung biodistribution and can induce adverse lung effects. However, we noted some inconsistencies in the toxicity dataset as a whole. For example, differences in physicochemical properties sometimes contributed to significant variations in toxicity. In this literature review, the physicochemical characteristics of silica, such as its chemical composition, primary particle size, and crystallinity, were taken into account. This selection effectively excluded many articles where no elementary physicochemical characterization was presented. Most studies associated toxic endpoints with crystallinity and size, whereas only a few linked toxicity to porosity, shape, surface charge, and surface chemistry. Studies using amorphous silica nanoparticles, aggregated or agglomerated states in vehicle or cell culture medium, were almost never included for this review. The reason for this exclusion is that these physicochemical characteristics considerably alter the overall size, shape, and surface area, potentially influencing the biological impact of the particles tested [55,87,88,138,139]. The presence of impurities, (e.g., metals) on nanoparticle surface may play an important role in nanosilica toxicity [135]. In addition, the modified surface of mesoporous silica nanoparticles by some surfactants or chemical agents can also modulate cytotoxic responses [140]. Interestingly, particle surface area could also be transformed by natural molecules, such as ascorbic acid. Indeed, ascorbic acid is known to generate hydroxyl radicals on the surface of quartz particles in the sponge *Chondrosia reniformis* [141]. Therefore, it appears critical to determine how these physicochemical variations influence lung toxicity. However, the nature of these variations remains unclear, limiting our understanding of the specific toxicities of each form of silica to considerations of crystalline structure and size.

Moreover, recent studies indicate that hydroxyl groups at the surface of silica—as with hydrophilic (geminal or vicinal hydroxyl structure) or hydrophobic (siloxane structure after silanols condensation) surfaces—could significantly change its biological toxicity [10]. Indeed, the presence of silanols at the surface of nanosilica or micrometric crystalline silica appears to better correlate with toxicity than their crystallinity [142]. Further studies will be required to verify this hypothesis. Finally, in silicosis patients, the association of crystalline silica particles with bacterial endotoxins, such as lipopolysaccharides (LPS), is well known to further disrupt autophagic degradation of alveolar macrophages, resulting in an accumulation of autophagosomes and disrupting lysosomal function [143]. In our review, very few studies were found that considered this type of variation in physicochemical conditions.

### 5.3. Influence of Silica Particle Generation Processes

For exposure and risk assessment in occupational settings involving nanoparticles, it is important to understand their mechanisms of release from non-nanostructured materials, or their industrial manufactured process characteristics [144,145,146]. The manufacturing method used to produce synthetic silica particles (such as precipitated, pyrogenic, or fumed silica), especially for amorphous silica nanoparticles, could strongly influence their adverse biological effects [147]. In addition, the toxicological specificities of micrometric or nanometric silica particles emitted after fracturing of nanostructured or nonstructured materials (e.g., by high-energy rotating tools) are very rarely addressed in the literature. Nevertheless, silica particles are commonly emitted through this process in a large number of occupational activities, such as construction. It is important to bear in mind that the freshness of fractured silica particles could also influence their toxicity. Indeed, according to some authors, the spatial organization of surface silanols is more toxic for freshly emitted particles [12,59,142]. Porter et al. [148] indicated that none of the doses of aged crystalline silica used in their study induced NO production. In contrast, induction of this highly cytotoxic product has been reported in vivo with freshly fractured silica particles [148]. The other important question is: can silica nanoparticles be emitted after fracturing? To our knowledge, this question still remains unsolved. More systematic studies are clearly required to verify these production process-dependent differences.

### 5.4. Silica and Lung Toxicity

In the past, most research was focused on micrometric silica particles measuring between 0.5 and 10 μm, mainly in crystalline form. More recently, synthetic amorphous nanosilica, due to its many industrial applications, has received greater attention. Studies suggest that these different forms may have distinct toxicological properties. Thus, nanosilica may present specific hazards to human health, including an enhanced ability to penetrate intracellular compartments in the lung and to access the systemic circulation [149]. It should be noted that the choice of cell type, treatment medium, culture system, and assay conditions, along with the animal model used in experimental studies, can influence the toxic responses to amorphous or crystalline silica, regardless of the particle size used [122,150]. The lack of in vitro and in vivo studies implementing crystalline silica nanoparticles, apart from that of Warheit and colleagues [46], makes comparison of toxicity mechanisms between amorphous nanosilica and micrometric crystalline silica essential, while awaiting the possible advent of crystalline silica nanoparticles. A general overview of lung toxicity induced by different types of silica is shown in Table 1 and Table 2. Alterations to lung inflammation, oxidative stress, cytotoxicity, and phagocytosis are induced not only by micrometric crystalline and amorphous silica nanoparticles, but also by micrometric amorphous silica, even if far fewer studies are available. Fibrosis and epithelial–mesenchymal transition have so far only been observed with amorphous silica nanoparticles and rarely with micrometric amorphous silica particles (due to the limited number of studies using this class of silica). Crystalline silica-induced granulomas are well known and responsible for silicosis in humans. However, observations with amorphous nanosilica lead us to suggest that this type of lesion could be induced by lower doses and following faster kinetics [61]. Correlation between in vitro and in vivo effects indicates that specific cells or tissues are potential targets of toxicity. The results of in vitro and in vivo experiments suggest that exposure to nanosilica, as well as mainly crystalline silica, could induce lesions via the activation of NF-kB signaling pathways, autophagy, and the inflammasome [35,67,80,81,101,120]. Finally, this review shows that the biological effects of amorphous silica nanoparticles and micrometric crystalline silica are relatively similar at the cellular and tissue levels, although we lack solid arguments on the occurrence of silicosis after exposure to amorphous silica nanoparticles. Comparison is much more difficult for crystalline silica nanoparticles and for micrometric amorphous silica particles due to the very low number of studies, knowing that negative studies are seldom published.

## 6. Conclusions

Despite the accumulated evidence of its toxicity, silica particles—whatever their size or crystallinity—continue to represent an intriguing subject for basic and applied research in a number of scientific and technical fields. This review is particularly timely given the relatively recent emergence of manufactured nanoparticles, but also the new light that has been shed on toxicological determinants at the micrometric scale. The pathogenicity of silica varies depending on the physicochemical characteristics of the particles involved. For 50 years, its crystalline nature and ability to trigger the generation of free radicals have been recognized as relevant and unique characteristics contributing to its toxicity. More recently, the surface chemistry of silica—such as the presence of hydroxyl groups (–OH)—has also been shown to play an important role.

Our understanding of the molecular mechanisms underlying the undesirable effects of silica particles is obviously far from complete. However, the future is more optimistic, thanks to the advent of new characterization methodologies allowing finer analysis and more precise quantification, for example, based on the presence of silanols at the surface of silica particles. Moreover, the presence of impurities within the crystalline structure, or surface coatings on the particles must be better taken into account in experimental studies to avoid drawing erroneous conclusions. Studies on the in vitro and in vivo toxicity of nanosilicas remain in the exploratory stage, and the toxicity mechanisms of nanosilicas are still poorly understood. Better understanding of nanosilicas toxicity is necessary to guarantee an optimal safety level, in particular in biomedical applications, including nanosilica uses in drug delivery systems to diagnose and treat various human diseases. Minimal silica characterization standards are needed for reporting and understanding their biological or environmental behaviors and implications. New experimental studies thus seem necessary to better understand the mechanisms underlying the toxicity of this large family of particles with, in particular, the possible discovery of new molecular targets such as surface-chemistry-specific interactions between certain cellular recognition systems and silica particles.

## Figures and Tables

**Table 1:** Summary of results in vivo studies in mice and rats.

Silica Types	Type of Effects	Inflammation	Fibrosis	Cell Death and Linked Mechanisms	Oxidative Stress	Epithelial–Mesenchymal Transition (EMT)	Granulomas	Muco-Ciliary Clearance and Other Effects
Crystalline, micrometric size	Acute (and subacute)	− ↑ total cell count (neutrophils, macrophages, dendritic cells, lymphocytes) − ↑ proteins (TNFα, IL-1αβ, HMGB1, IL-6, IL-8, IL-18, IL-33, MCP-1, BAFF) − NLRP3 inflammasome + STING pathways − SR-B1 is a silica receptor mediating inflammasome activation − M1 macrophages dominant at inflammatory sites	− ↑profibrotic markers (TGFβ1, CXCL10, PA, 10E4 antigen levels) − ↑ collagen markers (hydroxyprolin, MARCO receptor, collagen III) − M2 macrophages dominant at fibrotic sites − ↓ miR-138 in fibrotic lung tissues	− ↑ LDH activity (dose-dependent) − cell death by apoptosis, pyroptosis, necrosis, autophagy * − other cell defense mechanisms: phagocytosis − lysosome damage and autophagosome accumulation − ↑LC3B-II/LC3B-I, Beclin-1, Mer, and P62 − ↓mTOR and LAP-1	− ↑hydroxyl and nitric radicals − ↑HO-1 − ↑SOD activity − ↑iNOS and nitrotyrosine (localized in granulomatous regions and BALT) − NF-κB activation	− miR-138 inhibits EMT in silica-induced pulmonary fibrosis by regulating ZEB2 (zinc finger E-box-binding homeobox)	− granulomas (silicotic nodules) colocalized with silica − emphysema (↑neutrophil elastase and insoluble elastin) − ↑iNOS and nitrotyrosine (localized in granulomatous regions and BALT)	− impaired muco-ciliary structure and MUC5B production − ↑albumin concentration − ↑lung microsomal protein concentration (mediated by CYP4501A1 and 2B1)
	Chronic (and subchronic)	− ↑ total cell count (neutrophils size-dependent, macrophages, lymphocytes CD4 CD80) − ↑ proteins (TNFα, IFNγ, IL-1β) − NLRP3 inflammasome + STING pathway	− ↑ profibrotic markers (TGFβ1, CXCL10, IL-10, PDGFB, SULF1) − ↑ collagen markers (hydroxyprolin, soluble collagen, type I collagen) − lung tissue thickening, time-dependent fibrosis	No data		No data		
Crystalline, micrometric size	Acute (and subacute)	− ↑ macrophages accumulation	− early pulmonary fibrosis development	− ↑ LDH activity	No data	No data	No data	No data
	Chronic (and subchronic)		No data	No data		No data		
Amorphous, micrometric size	Acute (and subacute)	− ↑ total cell count (neutrophils) − ↑ proteins (TNFα, IL-1αβ, IL-6, IL-8, MIP-1α, MIP-2) − less severity compared with NPs	No data	− transient increase in LDH activity (not occurring at low doses) − cell death by apoptosis − other cell defense mechanisms: phagocytosis	No data	No data	No data	No data
	Chronic (and subchronic)	No data	No data	No data		No data		
Amorphous, nanometric size	Acute (and subacute)	− ↑ total cell count (neutrophils size-dependent, macrophages, lymphocytes) − ↑ proteins (TNFα, IL-1αβ, IL-5, IL-6, IL-8, IL-13, IL-17a, IL-18, MCP-1, MIP-1α) − NLRP3 inflammasome − PARP pathway	− ↑ Masson’s trichrome staining	− ↑ LDH activity − other cell defense mechanisms: phagocytosis	No data	− EMT activated by nanosilica + TGFβ1 − ↑ sex dependent (higher in females) of EMT markers (caveolin-1 and MMP-9)	− granulomas appearing even after 1 week of exposure to 1 mg/kg	No data
	Chronic (and subchronic)	No data	− ↑ dose-dependent collagen markers (hydroxyprolin)	No data		No data		

Note: * Autophagy: cellular process that allows the degradation of cytoplasmic components such as damaged or unwanted proteins or organelles after their capture in a double lipid membrane—the autophagosome; Phagocytosis: a critical mechanism through which innate immune cells eliminate microbes, necrotic or apoptotic cells, and mineral particles.

**Table 2:** Summary of results in vitro studies in epithelial cells, macrophages, and fibroblasts.

Silica	Inflammation	Fibrosis	Cell Death and Linked Mechanisms	Oxidative Stress	Epithelial–Mesenchymal Transition (EMT)	Muco-Ciliary Clearance and Other Effects	Type of Cells
Crystalline, micrometric size	− ↑ proinflammatory markers (IL-1β, HMGB1, IL-6, IL-8, MCP-1) − NLRP3 inflammasome pathway	− ↑ profibrotic markers (SULF1, FGF-2, bFGF) − ↑ thickening markers (collagen and fibronectin)	− ↑ LDH release − Cell death by apoptosis (ERK1/2, SFK and p38 phosphorylation)	− ↑ ROS release − NAD(P)H pathway	− ↑ EMT markers (MMP-2, MMP-9, Col-1, Col-3, α-SMA, vimentin) − miR-138 inhibits EMT in silica-induced pulmonary fibrosis by regulating ZEB2 (zinc finger E-box-binding homeobox	No data	Epithelial
	− ↑ proinflammatory markers (TNF-α, IFN-γ, caspase-1, IL-1αβ, HMGB1, IL-6, IL-8, IL-13, IL-18) − ↑ other molecules (ABA, pCREB, AP-1) − NLRP3 inflammasome + STING + cyclooxygenase (↑ COX-2)-prostaglandin (↑ PGE2) pathways	− ↑ profibrotic marker bFGF (surface-reactivity dependent) − TGF-β activated kinase (TAK) involved in silica-mediated COX-2 expression	− ↑ LDH release (dose dependent) − cell death by apoptosis necrosis, autophagy * − other cell defense mechanisms: phagocytosis *↑LC3, PI3K/Akt/mTOR pathway − ↑ LC3, PI3K/Akt/mTOR pathway	− ↑ ROS release (size dependent) colocalized in mitochondria − nuclear translocation of NF-κB (binding site in COX-2 promoter − NF-κB inducing kinase (NIK) involved in silica-mediated COX-2 expression	No data	No data	Macrophage
	− activation of cyclooxygenase-prostaglandin pathway	− ↑ fibrosis parameters (Masson’s trichrome staining, α-SMA, matrix contraction)	No data	No data	No data	No data	Fibroblast
Amorphous, micrometric size	− ↑ proinflammatory markers (IL-6, IL-8)	No data	− ↑ LDH release	− ↑ ROS release and intracellular GSH (higher compared to nanosize)	No data	No data	Epithelial
	− ↑ proinflammatory markers (TNF-α, IL-1αβ, MIP-1α) − NLRP3 inflammasome + cyclooxygenase (↑ COX-2) pathways	No data	− cell death by apoptosis, necrosis, autophagy + Ca^2+^ pathway − other cell defense mechanisms: phagocytosis	− ↑ ROS release colocalized in mitochondria and phagolysosomes and intracellular GSH − ↑ K+ efflux	No data	No data	Macrophage
	− activation of cyclooxygenase-prostaglandin pathway	No data	No data	No data	No data	No data	Fibroblast
Amorphous, nanometric size	− ↑ proinflammatory markers (TNF-α, CXCL1, IL-1 αβ, IL-6, IL-8, MIP-1αβ) − NLRP3 inflammasome + PARP pathways − SR-B1 is a silica receptor-mediating inflammasome activation	− colocalization of silica and TGF-β1 in cell membrane − ↑ phosphorylation of Smad2	− ↑ LDH release (dose dependent, not occurring at low doses) − cell death by apoptosis (p38 phosphorylation), autophagy − other cell defense mechanisms: phagocytosis through NF-κB activation − lysosome damage and impairment in autophagic flux	− ↑ ROS release (time and dose dependent), lipid peroxidation and intracellular GSH − ↑ HO-1 and 8-OHdG − mitochondrial dysfunction − downregulation of NRF-2 signaling − phosphorylation of MAPKs (p38 and JNK) and p65 − ERK pathway	− ↑ EMT marker vimentin (incubation with TGF-β1)	− ↓ciliary beat frequency	Epithelial
	− ↑ proinflammatory markers (TNF-α, caspase-1, IL-1αβ, IL-8, MIP-1α) − NLRP3 inflammasome pathway	− ↑ profibrotic marker TGF-β − M2 macrophages promotes NPs internalization	− ↑ LDH release (time and dose-dependent, only with highest doses) − cell death by apoptosis, autophagy − other cell defense mechanisms: phagocytosis	− ↑ ROS release (dose-dependent, in endosomal compartment) − NF-κB activation	No data	No data	Macrophage
	− SERPINB2 (plasminogen activator inhibitor 2) protein identified as a potential biomarker of inflammatory responses	− ↑ collagen markers (hydroxyprolin, type I collagen) − SERPINB2 protein identified as a potential biomarker of fibrosis	− ↓ cell viability (size dependent) − cell death by apoptosis	− no difference in ROS formation	− ↑ EMT markers (α-SMA)	No data	Fibroblast

Note: * Autophagy: cellular process that allows the degradation of cytoplasmic components such as damaged or unwanted proteins or organelles after their capture in a double lipid membrane—the autophagosome; Phagocytosis: a critical mechanism through which innate immune cells eliminate microbes, necrotic or apoptotic cells, and mineral particles.

## Data Availability

Not applicable.

## Supplementary Materials

The following are available online at https://www.mdpi.com/article/10.3390/nano12142392/s1, Tables S1–S7 summarize all the in vivo and in vitro studies according to the type of silica particle (i.e., according to its size and crystallinity).
