# Supplementary material for: Pulmonary Toxicity of Silica Linked to Its Micro- or Nanometric Particle Size and Crystal Structure: A Review
Source: Nanomaterials (Basel). 2022 Jul 13;12(14):2392. doi: 10.3390/nano12142392 (PMC9318389; doi:10.3390/nano12142392)
Supplement: Supplementary file 1 [file nanomaterials-12-02392-s001.zip › nanomaterials-1784645-Supplementary Materials.pdf]

nanomaterials

MDPI

Review

Pulmonary Toxicity of Silica Linked to Its Micro- or Nanometric Particle Size and Crystal Structure: A Review

Vanessa Marques Da Silva [1], Manon Benjdir [1], Pierrick Montagne [1], Jean-Claude Pairon [1,2], Sophie Lanone [1] and Pascal Andujar [1,2,*]

[1] University Paris Est Créteil, INSERM, Institut Mondor de Recherche Biomédicale, F-94010 Créteil, France; vanessa.marques-da-silva@inserm.fr (V.M.D.S.); manonbn@gmail.com (M.B.); montagnepierrick@gmail.com (P.M.); jc.pairon@chicreteil.fr (J.-C.P.); sophie.lanone@inserm.fr (S.L.)

[2] Hôpital Intercommunal de Créteil, Service de Pathologies Professionnelles et de l'Environnement, F-94010 Créteil, France

* Correspondence: pascal.andujar@chicreteil.fr; Tel.: +33-1-57-02-20-95

Table S1. In vivo studies, micrometric crystalline silica.

Exposure Route	Animal Model	Dose Duration	Silica Name and Supplier	Primary Particle Size	Tests	Main Results	Reference
Whole-body Inhalation	Rats Lewis, male (adults, 6-8 weeks) (*n* = 5)	6.2 mg/m^3 (6 h/ day, 5 days/ week during 6 weeks)<br><br>Timepoints:<br>- Stage 1: 4-28 days post exposure<br>- Stage 2: 10 weeks post exposure	Crystalline:<br>- Min-U-Sil (U.S. Silica co)	1.75 µm	- Silica content in lung<br>- Cytotoxicity (LDH release)<br>- BAL analysis<br>- Flow Cytometry<br>- Histopathology	- Silica deposition in lung at both stages.<br>- Significant increase of LDH release at stage 2.<br>- Significant increase of protein concentration in BAL at stage 2.<br>- Neutrophils and lymphocytes infiltration at stage 2<br>- Granulomatous changes around silica-containing epithelioid macrophages at stage 2.	Langley, et al. Am J Respir Cell Mol Biol 2004
Whole-body Inhalation	Rats Fischer 344, male (Age N/A) (*n* = 5–15)	15 mg/m^3 (6 h/ day during 5 days)<br><br>Timepoints:<br>- 5, 10, 16, 20, 30, 41, 79 days (acute and sub-chronic)<br>- 116 days (chronic)	Crystalline:<br>- Min-U-Sil (U.S. Silica co)	≤ 2 µm	- Cytotoxicity (LDH release)<br>- BAL and blood cell differentials analysis<br><br>- NF-κB gel shift assay<br><br>- ELISA (TNF-α and IL-1)	- Elevation of LDH relatively constant the first 30 days and steadily increase until 116 days of exposure.<br>- Significant increase of BAL and blood neutrophils at every exposure time.<br>Significant increase of alveolar macrophages and blood monocytes after 41 days until 116 days of exposure.<br>- NF-κB activation in BAL cells occurred after 5 days and continue to increase until 116 days of exposure.<br>- Significant production of TNF-α and IL-1 in BAL cells after 10 days until 116 days of exposure.	Porter, et al. Inhal Toxicol 2002
Whole-body Inhalation	Rats Fischer 344, male (Age N/A) (*n* = 5–15)	15 mg/m^3 (6 h/ day during 5 days/week)	Min-U-Sil (U.S. Silica co)	1.61 µm	- BAL analysis (differential and NOx)<br>- NF-κB/DNA binding	- Significant elevation of PMN and NOx but stable new level through the first 41 days and increased at a steep rate thereafter.	Castranova, et al. Mol Cell Biochem 2002

		Timepoints:<br>- 5, 10, 16, 20, 30, 41, 79 days (acute and sub-chronic)<br>- 116 days (chronic)			- ELISA (TNF-α and IL-1)<br>- Activation of alveolar type II epithelial cells<br>- Hydroxyproline content<br>- Zymosan-stimulated AM chemiluminescence<br>- Immunohistochemistry (iNOS and nitrotyrosine)	- Linear increase with continued activation after 5 days of silica exposure.<br>- Significant production of TNF-α and IL-1 after 41 days of exposure.<br>- Significant elevation of hypertrophy, hyperplasia and lipid production but stable new level through the first 41 days and increased at a steep rate thereafter.<br>- Significant increase of hydroxyproline after 116 days of exposure.<br>- ROS measurable only after 41 days of exposure.<br>- iNOS and nitrotyrosine localized in granulomatous regions and BALT.	
Whole-body Inhalation	Rats Crl:CD(SD)IGS BR<br>(Sex N/A)<br>(Age N/A)<br>(*n* N/A)	- Min-U-Sil 5: 100 mg/m³<br>(6 h/day, 3 days)<br>- α-cristobalite: 10 or 100 mg/m³<br>(3 days)<br>Timepoints:<br>- 10, 20, 30, 40, 50, 60, 70, 80, 90 days post exposure	Crystalline:<br>- Min-U-Sil 5 (Pittsburgh Glass and Sand Corp)<br>- α-cristobalite (C&E Mineral Corp)	- 3.3–3.5 μm<br>- 3.4–3.6 μm	- Cytotoxicity (LDH release)<br>- BAL analysis (protein content and N-acetyl glucosaminidase)	- Significant and persistent increase of LDH release at all timepoints.<br>- Significant and persistent increase of neutrophil recruitment and NAG at all timepoints.<br>(Results higher for α-cristobalite 100 mg/m³)	Warheit, et al. Scand J Work Environ Health 1995
Whole-body Inhalation	Rats Fischer 344, male<br>(Age N/A)<br>(*n* N/A)	9.4 and 10.8 mg/m³<br>(6 h/day, 5 days/week, 10 days)	Crystalline:<br>- Cristobalite (C&E Mineral Corp	N/A	- Western Blot (Mn-SOD)<br>- Immunocytochemical localization of Mn-SOD	- Significant increase of Mn-SOD.<br>- Mn-SOD located predominantly in mitochondria of type II epithelial cells.<br>- Number of immunogold particles per unit of mitochondrial area	Holley, et al. Am J Pathol 1992

		Timepoint:<br>- 10 days post exposure			- Ultrastructural morphometry<br>- Histopathology	increased in the terminal airways of lung.<br>- Increased size of type II epithelial cells (morphometry), number of interstitial macrophages and neutrophils in the terminal respiratory tissue.	
Whole-body Inhalation	Rats Fischer 344, male (Age N/A) (*n* = 4)	7 and 10 mg/m^3 (6 h/day, 5 days/week, 1, 3, 6, 9 or 10 days)<br><br>Timepoint:<br>- 1, 3, 6, 9 days post exposure<br>- 24 days post exposure	Crystalline:<br>- Cristobalite (C&E Mineral Corp)	2.6 µm	- Northern Blot<br>- Antioxidant enzymes (AOE) activity<br>- Western Blot<br>- BAL analysis<br>- Hydroxyproline content	- Significant increase of MnSOD mRNA at all timepoints.<br>- Significant increase of glutathione peroxidase mRNA at 9 days post exposure.<br>- No significant differences for AOE activity.<br>- Significant increase of MnSOD protein.<br>- Significant increase of total cell count, neutrophils and alkaline phosphatase at all timepoints.<br>- Significant and time-dependent increase of hydroxyproline content.	Janssen, et al. J Biol Chem 1992
Whole-body Inhalation	Rats Crl:CD(SD)IGS BR, male (Adults, 7-10 weeks) (*n* N/A)	100 mg/m^3<br>(6 h)<br>± CVF (cobra venom factor to inhibiting the complement)<br>Timepoints:<br>- 0, 24, 48 h, 8 days or 1 month post exposure	Crystalline:<br>- Min-U-Sil (Pittsburgh Glass and Sand Corp)	3.8 µm	- Cytotoxicity (LDH release)<br>- BAL analysis<br>- Pulmonary macrophage studies by scanning electron micrographs (SEM)	- Significant increase of LDH release at all timepoints.<br>- Significant and sustained increase of neutrophils, alkaline phosphatase and protein content at all timepoints.<br>- Deficit in pulmonary macrophage phagocytic function.<br>- No effects in rats treated with CVF suggesting complement may not play an important role in the acute pulmonary response to silica.	Warheit, et al. Environ Res 1991

Whole-body Inhalation	Rats Lister (8 weeks–8 months)<br>Guinea pigs (Strain, sex and age N/A)<br>(*n* N/A)	3–36 mg/m^3 (Overnight: from 6 to 18 h, 5 days/week)<br>± polyvinylpyridine-N-oxide (PNO) treatment<br><br>Timepoints:<br>- 6 to 14 months	Crystalline:<br>- Min-U-Sil<br>- Dowson and Dobson<br>- DQ-12	<5 µm	- Histology	In rats:<br>- PNO treatment abolish fibrosis of type II cells and lipidosis.<br>- Inhalation of low concentration over a long period promotes nodulation.<br>Guinea pigs:<br>- Massive alveolar accumulation of dust-bearing macrophages and type II cell hyperplasia but not lipidosis.	Heppleston. Br J Exp Pathol 1986
"Nose only" Inhalation	Rats Fischer 344, male<br>(Age N/A)<br>(*n* = 5)	15 mg/m^3<br>(6 h/ day during 5 days)<br><br>Timepoints:<br>- 20, 40, 60 days post (± 36 days of ecovery)	Crystalline:<br>- Min-U-Sil<br>(U.S. Silica co)	N/A	- BAL analysis (SOD activity and NOx concentration)<br>- NO-dependent chemiluminescence<br>- Lung lipid peroxidation<br>- Immunohistochemistry<br>- Counting and classifying macrophages for iNOS and silica	- Significant increase of state of oxidative stress, severity increasing in 36 days recovery groups.<br>- Significant increase in lung NO production, increasing in 36 days recovery groups.<br>- Significant increase of lipid peroxidation with or without recovery.<br>- Presence of silica particles in alveolar macrophages highly associated with iNOS protein.	Porter, et al. Toxicol Sci 2006
"Nose only" Inhalation	Rats Fischer 344, male<br>(Age N/A)<br>(*n* = 5)	15 mg/m^3<br>(6 h/ day, 5 days/ week during 21 or 59 days)<br>± IT of *L. monocytogenes*<br><br>Timepoints:<br>- 0, 3, 7 days post Listeria instillation	Crystalline:<br>- Min-U-Sil<br>(U.S. Silica co)	1.4 µm	- Cytotoxicity (LDH release)<br>- BAL analysis<br>- Pulmonary clearance of *L. monocytogenes*<br>- Chemiluminescence (release of ROS)	- Significant increase of LDH activity after 59 days.<br>- Significant increase of neutrophils after 59 days.<br>- Significant enhanced of pulmonary clearance after 21 and 59 days.<br>- Significant increase of chemilumiscence in phagocytes after 59 days.	Antonini, et al. Lung 2000
Intratracheal Instillation	Rats Wistar, male	250 mg/kg	Crystalline	0.5–10 µm	- Histopathology<br>- RT-PCR	- Positive Masson-Trichrome staining from 28 to 56 post IT.	Li, et al. Environ Toxicol

	(adult)<br>(n = 6)	Timepoints:<br>7, 14, 28, 56 days post IT (acute and sub-chronic)			- ELISA<br>- TEM	- Significant and time-dependent increase of collagen III mRNA.<br>- Significant increase of TNF-α and TGF-β.<br>- Persistent activation of autophagy in the development of pulmonary fibrosis.	2021
Intratracheal Instillation	Mice C57BL6 WT, Gas6$^{-/-}$ and Mer$^{-/-}$, male (young, 6–8 weeks)<br>(n = 3–6)	2.5 mg/mouse (unique)<br><br>Timepoints:<br>- 7, 28 and 84 days post IT (acute and sub-chronic)	Crystalline (National Institute of Occupational health and Poisons Control, Chinese Centers for Disease Control and Prevention)	<5 µm	- TEM analysis<br><br>- Western blot (LC3, Beclin1, ATG5, p-mTOR, P62, LAMP1, Mer, Tyro3)<br>- RT-qPCR	- Lysosome damage and autophagosomes accumulation in lung tissues of WT mice.<br>(Reduction of autophagosome formation in Gas6$^{-/-}$ and Mer$^{-/-}$ mice)<br>- Significant increases of LC3B-II/LC3B-I ratio, ATG5 (only after 7 days), Beclin-1, Mer, and P62<br>Significant decreases of p-mTOR and LAMP1<br>- Elevation of mRNA expression of ATG5, Beclin-1, Mer receptor in WT mice.	Li, et al. Toxicol Lett 2021
Intratracheal Instillation	Rats Wistar, male (Adults, 5–7 weeks)<br>(n = 15)	50 mg/rat (unique)<br><br>Timepoints:<br>1, 7, 14, 21 and 28 days post intranasal instillation (acute and sub-chronic)	SiO_2 dust particles (Sigma-Aldrich)	1–5 µm	- Histopathology<br><br>- Short time-series expression miner analysis	- Inflammatory cells appeared in the lung from the first day and peaked on day 7.<br>Fibroblasts appeared along with inflammatory cells decreasing gradually from day 14 (crucial timepoint in silicosis progression).<br>On day 28, extensive fibrosis appeared and the typical silicon nodules formed gradually.<br>- Number of altered LncRNAs (Long non-coding RNA) increased with exposure time. 149 LncRNAs were	Sai, et al. Genes Environ 2021

					- KEGG pathway and Gene Ontology analysis	increased and 136 LncRNAs decreased with 5 significant temporal expression patterns.<br>- 4 signaling pathways (“Complement and coagulation cascades”, “Vascular smooth muscle contraction”, “Tight junctions” and “Central carbon metabolism in cancer”) may be associated with the development of silicosis.	
Intratracheal Instillation	Mice C57BL6, male (Young, 6–8 weeks) (n = 8)	50 mg/kg (unique)<br>± miR-138 overexpression<br><br>Timepoints:<br>7, 14, 21, 28 days post IT (acute and sub-chronic)	S5631 (Sigma-Aldrich)	1–5 μm	- RT-qPCR<br>- Western Blot<br><br>- Immunohistochemistry	- Decreased expression of miR-138 in fibrotic lung tissues of mice.<br>- After transfection with miR-138, expression levels of α-SMA and vimentin were significantly increased and E-cadherin significantly decreased.<br>Overexpression of miR-138 alleviates silica-induced pulmonary fibrosis.<br>- Up-regulation of miR-138 reduced ZEB2 levels.	Wu, et al. Toxicology 2021
Intratracheal Instillation	Mice C57BL6, male (age N/A) (n = 24)	2.5 mg/mouse (unique)<br>± anti-HMGB-1 or recombinant HMGB-1<br><br>Timepoints:<br>- 7-, 28- and 84-days post IT (acute and sub-chronic)	Crystalline:<br>- Dörentrup quartz (87% α-quartz and 13% amorphous silica)<br>- DQ12 (Institute for Occupational Safety and Health of German Social Accident Insurance)	0.96 μm	- BAL analysis<br><br>- ELISA assay (HMBG-1, TNF-α and IL-6)<br>- RT-qPCR (TNF-α, IL-6, Col1a1 and fibronectin)<br><br>- Western blot	- Accumulation of macrophages and neutrophils decreased by anti-HMGB-1 treatment.<br>- IL-6 and TNF-α levels significantly elevated at all timepoints in mice exposed to silica only.<br>+ anti-HMGB-1: alleviation of IL-6 and TNF-α expression 28- and 84-days post IT<br>+ rm-HMGB-1: aggravation of this process<br>- Significant decrease of Col1a1 and fibronectin protein expression at days	Ma, et al. Toxicol Lett 2020

					- Histopathology	28 and 84 in groups exposed to silica and to silica +anti-HMGB-1.<br>- Greater and time-dependent infiltration of inflammatory cells (neutrophils and foamy macrophages) and alveolar septal thickening at all timepoints in mice exposed to silica only. Alveolar lipoproteinosis + granulomatous inflammation around the bronchus and alveoli at day 84.<br>+ anti-HMGB-1: less inflammatory cell infiltration and alveolar structure changes.<br>+ rm-HMGB-1: inflammation increased further.	
Intratracheal Instillation	Mice C57BL6, male (young, 5 weeks) (n = 2–3)	2.5 mg/mouse (unique)<br><br>Timepoints:<br>- 1-, 7-, 28- and 84-days post IT (acute)	Crystalline:<br>- α quartz (from National Institute of Occupational Health and Poison Control, China)	<5 μm	- Histological analysis:<br>Morphologic changes of cilia on airway epithelium:<br>. Hematoxylin and eosin (HE) staining<br>. Mucus production in trachea (Alcian blue – periodic acid Schiff (AB-PAS) staining<br>-Transmission electron microscopy analysis (TEM): Ultrastructural changes of cilia on airway epithelium	- Mucociliary structure impaired:<br>. Disordered, shortened or partially lost cilia on the surface (from day 7 to day 84).<br>. Increased mucus in mucous layer and submucosal glands (from day 7 to day 84).<br>- Ultrastructural abnormalities: absence of central pair of microtubules, disorganized microtubules and clusters of axonemes.<br>Significant decrease of numbers of ciliary axonemes and basal bodies.<br>Significant increase of abnormal axonemes.<br>- Significant decrease of MUC5B expression on the surface of airway epithelium on day 28 and 84.	Yu, et al. Exp Lung Res 2020

					- Immunohistochemistry	Significant increase of MUC5B in submucosal glands from day 1 to day 84.	
Intratracheal instillation	Mice C57BL6, male (young, 6–8 weeks) (*n* = 8)	2.5 mg/mouse (unique)<br><br>Timepoints:<br>- 1-, 3-, 7-, 14-, 28-, 42- and 56-days post IT (acute and sub-chronic)	Crystalline: (Sigma-Aldrich)	1.4 µm	- Flow cytometry<br>- RT-qPCR<br>- ELISA assay (IL-1β, TNF-α, TGF-β1 and IL-10)<br>- Western Blot<br>- Immunohistochemistry: markers of M1 (iNOS and CD86) / markers of M2 (CD206 and Arg1)	- Same change tendency for M1 and M2 macrophages (increasing peak at day 14) with higher M1 proportion compare to M2.<br>- Peak levels at day 7 for IL-6, day 14 for TNF-α and day 28 for IL-1 β. IL-10 expression continually rose.<br>- TNF-α and IL-1β increased until day 28 and then decreased.<br>IL-10 decreased at early stage and increased until day 56.<br>- STAT1 and IRF3 implicated in M1 polarization and correspond to expression of M1 marker iNOS. STAT6 and PPAR-γ implicated in M2 polarization and correspond to expression of M2 marker CD206.<br>- M1 and M2 macrophages mainly accumulated in lesioned areas<br>M1 macrophages dominant at inflammatory sites<br>M2 macrophages dominant at fibrotic sites	Zhao, et al. Ecotoxicol Environ Saf 2020
Intratracheal instillation	Mice C57BL6 (adults, 8-12 weeks)<br>- WT<br>- KO (STING$^{-/-}$, cGAS$^{-/-}$, IFNAR$^{-/-}$, TLR9$^{-/-}$, IL-	1 mg/mouse (unique)<br>± DNase I treatment (degrade extracellular dsDNA)<br><br>Timepoints:<br>- 7 days and 4 weeks	Crystalline:<br>- DQ-12 (quartz)	2.2 µm	- Measurement of double-stranded DNA<br>- RT-qPCR<br>- ELISA (CXCL10)<br>- Multiplex (IFN-αβ)<br>- Flow cytometry	- Release of self-dsDNA in bronchoalveolar space, activating STING pathway in WT and TLR9$^{-/-}$ mice.<br>+ DNase I: release of self-dsDNA reduced.<br>- Overexpression of STING and cGAS genes at all timepoints.	Benmerzoug, et al. Nat Commun 2018

	1R1$^{-/-}$, TLR2/4$^{-/-}$, post IT NLRP3$^{-/-}$)<br>(*n* = 4–5)<br><br>STING = Stimulator of interferon genes		- Quantification of mitochondrial (mtDNA) versus nuclear DNA (nDNA)<br>- Immunoblots:<br>. cleaved caspase-3 (apoptosis)<br>. cleaved gasdermin D (pyroptosis)<br>. phosphorylation of mixed lineage kinase domain-like pseudokinase (MLKL, necroptosis)	+ DNase I: overexpression of STING reduced.<br>- Significant increase of CXCL10 at all timepoints in WT mice.<br>+ DNase I: significant decrease of CXCL10.<br>Significant increase of IL-1β, TNF-α and IFN-γ 4 weeks post IT in WT and TLR9$^{-/-}$ mice.<br>- Significant increase of type I IFNs at all timepoints in WT mice.<br>In TLR9$^{-/-}$ mice: IFN-β release only.<br>+ DNase I: significant decrease of type I IFNs.<br>- Significant increase of cell death (of interstitial and alveolar macrophages but also neutrophils) excepting in mice with DNase I treatment.<br>- Significant increase of mtDNA and nDNA in WT mice.<br>- Significant caspase-3 cleavage, gasdermin D cleavage and phosphorylation of MLKL.<br>+ DNase I: caspase-3 cleavage and phosphorylation of MLKL reduced.<br>-STING$^{-/-}$ and cGAS$^{-/-}$ mice exhibited a reduction of all parameters of silica-induced lung inflammation.<br>Same for NLRP3$^{-/-}$ excepting for the release of self-dsDNA.<br>- DNase I: potential therapy for silica-induced lung inflammation?

Intratracheal instillation	Rats Wistar, female (adults, 8 weeks) (*n* = 4)	2 mg/rat<br><br>Timepoint:<br>- 3- and 90-days post IT	Crystalline:<br>- cristobalite (C&E Mineral Corp)<br>- DQ-12 (quartz) (IUF Dusseldorf)	N/A	- Immunohistochemistry (SULF1 and 10E4)	- Decrease SULF1 protein levels and increased 10E4 antigen levels at 3 days post IT but reverse effects at 90 days post IT.	Perkins, et al. Toxicol Appl Pharmacol 2018
Intratracheal instillation	Rats (from own breeding colony), female (age N/A) (*n* N/A)	7 mg/mL (unique)<br><br>Timepoints:<br>- 24 h (acute)	Crystalline:<br>- DQ-12 (quartz)	< 5µm	Acute:<br>- Cytotoxicity in BAL	- Significant increase of total cells, alveolar macrophages and neutrophils.	Sutunkova, et al. Toxicology 2017
Intratracheal instillation	Mice C57BL6 (Scarb1$^{+/-}$ and WT), male (age N/A) (*n* = 3–5)	1 or 5 mg/mouse<br>± AOB-1 pretreatment (masking SR-B1 receptor)<br><br>Timepoints:<br>- 24 h and 6 weeks post IT (acute and sub-chronic)	Min-U-Sil (U.S. Silica co)	N/A	- Microcomputed tomography<br>- BAL analysis<br>- ELISA (TNF-α, IL-1α and IL-1β)<br>- Histopathology	- High intensity signal by accumulation of inflammatory cells in alveolar spaces.<br>- Significant increase of neutrophils number.<br>- Significant increase of pro-inflammatory cytokines.<br>AOB-1 pretreatment ameliorated pathological inflammation.<br>- Large areas of fibrosis with 5 mg of silica with no amelioration by AOB-1 pretreatment.<br>Small areas of fibrosis with 1 mg of silica with amelioration by AOB-1 pretreatment.	Tsugita, et al. Cell Rep 2017
Intratracheal instillation	Mice C57BL6, female<br>- WT<br>- KO (IL-1$\alpha^{-/-}$ and IL-1$\beta^{-/-}$)<br>(Adults, 8-12 weeks)<br>(*n* N/A)	2.5 mg/mouse (unique)<br><br>Timepoints:<br>- 1, 3, 6, 12, 24 h post IT (acute)	Crystalline:<br>- DQ-12 (quartz) (DMT GmbH and Co)	960 nm	- BAL analysis<br>- RT-qPCR<br>- ELISA	- Significant increase of total cell count and neutrophils.<br>- Significant increase of IL-1α 1h post IT (less important compared with amorphous silica)<br>Early release (1h post IT) of endogenous gene and protein IL-1α and IL-33 precedes silica-induces IL-1β	Rabolli, et al. Part Fibre Toxicol 2014

						production and neutrophilic inflammation.<br>Significant increase of IL-1β 24 h post IT (less important compared with amorphous silica).<br>- Reduction of IL-1β and neutrophil accumulation in mice IL-1$\alpha^{-/-}$.	
Intratracheal instillation	Rats Wistar, female<br>(Adults, 8 weeks)<br>(*n* = 5)	2 mg/rat<br>(unique)<br><br>Timepoints:<br>- 3, 7, 28, 90,180, 360 days post IT	Crystalline:<br>- DQ-12 (quartz)<br>(IUF Dusseldorf)<br>± PVNO	N/A	- Histological analysis (H&E and Sirius Red)<br>- Immunohistochemistry<br>- Western Blot	- Silicotic nodules at 180 and 360 days.<br>- Increased levels of caspase-1 and IL1-β in DQ-12 group.<br>- Significant increase of caspase-1 activity in BALF of DQ-12 group at day 3 and 7.<br>- PVNO attenuated the grade of inflammation, lymphocyte influx and inflammasome activation	Peeters, et al.<br>Part Fibre Toxicol<br>2014
Intratracheal instillation	Mice C57BL6<br>(sex N/A)<br>- WT<br>- KO (MyD88$^{-/-}$)<br>(age N/A)<br>(*n* = 4–6)	2.5 mg/mouse<br>(unique)<br><br>Timepoint:<br>- 60 days post IT	Crystalline:<br>- DQ-12 (quartz)	2.2 µm	- BAL analysis<br>- Hydroxyproline content<br>- Flow cytometry<br>- RT-qPCR<br>- ELISA (IL-1β and PDGF-BB)<br>- Histological analysis<br>- Immunohistochemistry	- Significant increase of neutrophils and macrophages only for WT.<br>- Significant increase of OH-content for both mice.<br>- Significant increase of LyT CD4$^+$ but not LyT CD8$^+$ in WT mice.<br>- Significant increase of TGFβ1 and IL-10 for both mice.<br>- Significant increase of IL-1β only for WT.<br>- Significant increase of increase of PDGF BB for both mice.<br>- Granuloma formation and neutrophil infiltration less pronounced in MyD88 KO mice but lung collagen formation remaining for both mice:<br>. Pulmonary fibrosis localized in	Lo Re, et al.<br>PLOS One<br>2014

						granulomas for WT mice.<br>. Pulmonary fibrosis diffusely distributed throughout the parenchyma and lymphocyte accumulation in MyD88KO mice.<br>- MyD88-related immunity is central in the establishment of particle-induced lung inflammatory and granuloma responses.	
Intratracheal instillation	Mice NMRI, C57BL6 and DBA/2<br>(sex N/A)<br>(adults, 8 weeks)<br>(*n* = 3–4)	2.5 mg/mouse<br>(unique)<br><br>Timepoints:<br>- 3, 5, 10, 15, 20, 30, 60 days post IT	Crystalline<br>- DQ-12 (quartz)	2.2 µm	- BAL analysis<br>- Hydroxyproline content and soluble collagen assays<br>- RT-qPCR<br>- ELISA (IL-10, TGF-β)	- Significant increase of neutrophils and proteins at all timepoints. Significant increase of macrophages and LDH activity except at 10 days post IT.<br>- Significant increase of hydroxyproline and soluble collagen at 60 days post IT.<br>- Significant increase of TNF-α, IL-1β and KC at 3 days post IT<br>- Significant increase of IL-10 and TGF-β 60 days post IT.<br>- NMRI mice treated with dexamethasone had reduced lung injury, cellular inflammation and secretion of pro-inflammatory cytokines but no effects in lung fibrosis and expression of the fibrogenic and suppressive cytokines TGF-β and IL-10	Rabolli, et al. Toxicol Lett 2011
Intratracheal instillation	Rats Crl:CD BR, male<br>(Adult, 8 weeks)<br>(*n* = 5)	1 or 5 mg/kg<br>(unique)<br>Timepoints post-IT:<br>- 24 h, 1 week,	- Min-U-Sil<br>(U.S. Silica co)<br>- Fine-quartz particles	- 0.5 µm<br><br>- 0.3 µm	- Haemolytic potential<br>- Cytotoxicity (LDH release)<br>- Histopathology	- High haemolytic potential for Min-U-Sil.<br>- Significant increase of LDH release, higher for Min-U-Sil.	Warheit, et al. Toxicol Sci 2007

		1 month (acute and sub-chronic)<br>- 3 months (chronic)	(Synthetized hydrothermally)			- Typical quartz-related effects dose-dependent lung inflammatory macrophage accumulation responses concomitant with early development of pulmonary fibrosis, higher for Min-U-Sil.	
Intratracheal instillation	Rats Wistar, male<br>(adults, 10 weeks)<br>(*n* = 5)	1 or 2 mg/rat (unique)<br><br>Timepoints:<br>- 3 days, 1 week, 1, 3, 6 months	Crystalline:<br>- Min-U-Sil<br>(U.S. Silica co)	1.6 µm	- Western Blot (HO-1)<br>- Immunohistochemistry (HO-1)	- Significant increase of HO-1 after 3 days in rats exposed at 2 mg of silica. Significant increase of HO-1 after 1 to 6 months for both concentrations.<br>- HO-1 positive cells found particularly in the alveolar macrophages.	Nagatomo, et al. J Occup Health 2006
Intratracheal instillation	Rats Crl:CD(SD)IGS BR, male<br>(adults, 8 weeks)<br>(*n* = 5)	1 or 5 mg/kg (unique)<br><br>Timepoints:<br>- 24 h, 1 week, 1 and 3 months	Crystalline:<br>- Min-U-Sil<br>(Pittsburgh Glass and Sand Corp)	1.5 µm	- BAL analysis<br>- Cell proliferation assay<br>- Histopathology	- Significant and dose-dependent increase of pulmonary inflammation and cytotoxicity at all timepoints. Significant increase of LDH release at all doses and timepoints.<br>- Significant increase of tracheobronchial cell proliferation rate only for 5 mg/kg after 24 h of exposure.<br>- Dose-dependent lung inflammatory response (neutrophils and foamy multinucleated alveolar macrophages accumulation). Lung tissue thickening after 3 months of exposure.	Warheit, et al. Part Fibre Toxicol 2006
Intratracheal instillation	- Rats Sprague-Dawley, female<br>- Mice NMRI, female<br>(age N/A)<br>(*n* = 5–7)	2.5 and 25 mg/g lung: (unique)<br>- 0.5 or 5 mg/mouse<br>- 3 or 30 mg/rat<br><br>Timepoints:<br>- 3, 30, 60 days post IT	Crystalline:<br>- DQ-12 (quartz)	2.2 µm	- Cytotoxicity (LDH release)<br>- BAL analysis<br>- Hydroxyproline content<br>- ELISA (type I collagen, IL-10, TNF-α)	- Significant increase of LDH release at all timepoints (except for mice 60 days post IT).<br>- Significant increase of protein and neutrophils at all timepoints (except for mice 30- and 60-days post IT).<br>- Significant increase of hydroxyproline content with high dose in both	Barbarin, et al. Respir Res 2005

					- Immunohistochemistry<br>- Histopathology	species, 60 days post IT.<br>- Significant increase of type I collagen with high dose in both species, 60 days post IT.<br>Significant increase of TNF-α with high dose, 30- and 60-days post IT in rats only.<br>Significant increase of IL-10 for both doses 60 days post IT in mice only (linked with resolution of alveolitis).<br>- Silicotic nodules with high dose in both species, 60 days post IT.	
Intratracheal instillation	Hamsters Syrian Gold, male and female<br>(Age N/A)<br>(n = 4–7)	2, 20, 200 µg/hamster (unique)<br><br>Timepoint:<br>- 24 h post IT	Crystalline:<br>- Min-U-Sil	2 µm	- BAL analysis<br>- Elastase inhibition during lung inflammation and thrombosis	- Significant increase of macrophages and neutrophils in BAL (20 and 200 µg).<br>(Increase significantly reduced when hamsters were depleted of alveolar macrophages and neutrophils).<br>Significant increase of neutrophil elastase level (20 µg).<br>- No effects in lung inflammation and thrombosis.	Nemmar, et al. Am J Respir Crit Care Med 2005
Intratracheal instillation	Rats Wistar, male<br>(age N/A)<br>(n = 6)	2.3 mg/rat (unique)<br><br>Timepoints:<br>- 2, 7, 28, 90 days post IT	Crystalline:<br>- Min-U-Sil	0.5–3 µm	- Cytotoxicity (LDH release)<br>- BAL analysis<br>- Lung weight<br>- Histopathology	Results for all timepoints:<br>- Significant increase of LDH.<br>- Significant increase of protein concentration, total cell count and neutrophils.<br>- Significant increase of dry left lung weight.<br>- Progressive and time-dependent fibrosis.	Xu, et al. Toxicol Appl Pharmacol 2004

Intratracheal instillation	Rats Sprague-Dawley, male (Adults, 10 weeks) (*n* = 5)	5 or 20 µg/rat (repeated each week)<br><br>Timepoint:<br>12 weeks (sub-chronic)	α-quartz (Generic Respirable Dust Technology Center)<br>- freshly fractured<br>- aged	3.7 µm	- Cytotoxicity (LDH release)<br>- BAL analysis<br><br>- Zymosan-stimulated AM chemiluminescence	- Significant increase for mice weekly exposed to 20 µg of freshly fractured or aged silica.<br>- Significant increase of PMNs for mice weekly exposed to both doses of freshly fractured or aged silica.<br>- Greater for rats exposed to freshly fractured silica (=greater generation of ROS).	Porter, et al. Toxicology 2002
Intratracheal instillation	Mice Tg5 (transgenic, expressing mRNA IL-9 in all organs), FVB (WT), C57BL/6, female (adults, 8 weeks) (*n* = 5–12)	1 or 5 mg/mouse (unique)<br><br>Timepoints:<br>- 2, 4, 6 months post IT	Crystalline:<br>- DQ-12 (quartz)	2.2 µm	- Cytotoxicity (LDH release)<br>- BAL analysis<br>- Flow cytometry<br>- ELISA (IFN-γ, IL-4, IgG1, IgG2a)<br>- Hydroxyproline content<br>- Immunohistochemistry	- Significant and dose-dependent increase of LDH release in all mice groups.<br>- Significant and dose-dependent increase of protein concentration in all mice groups.<br>- Similar recruitment of macrophages, neutrophils and CD4 and CD8 in all mice groups.<br>Significant expression of B-lymphocyte population in BAL and parenchyma in Tg5 mice and C57BL/6 treated with IL-9 IP.<br>- Significant increase of IL-4 and IgG1/IgG2a ratio (type 2 immune response) in WT mice but not in TG5 mice.<br>Significant decrease of IFN-γ in all mice groups.<br>- Severity of fibrosis significantly less important in Tg5 mice.<br>- IL-4 co-localized in inflammatory macrophages.<br>Antifibrotic effect of IL-9	Arras, et al. Am J Respir Cell Mol Biol 2001

Intratracheal instillation	Rats Fischer 344, male (Age N/A) (*n* = 5)	- 3 (low) or 15 (high) mg/rat (unique: single-dose protocol)<br>- 0.6 (low) or 3 (high) mg/rat (5 daily instillations: multiple-dose protocol)<br><br>Timepoints:<br>- 14 days for low and high doses experiments<br>- 28 days for high dose	Crystalline:<br>- Min-U-Sil (U.S. Silica co)	3.5 µm	- BAL analysis<br>- Albumin concentration (capillary-epithelial barrier)	- Significant increase of total cells for both doses after 14 and 28 days for both single and multiple-dose protocol.<br>Significant increase of alveolar macrophages, neutrophils and lymphocytes 28 days for both single and multiple-dose protocol.<br>- Significant increase of albumin concentration 28 days for both single and multiple-dose protocol.<br>Multiple-dose protocol does not offer any advantages over the single-dose protocol.	Reasor and Antonini,<br>J Toxicol Environ Health<br>2001
Intratracheal instillation	Mice NMRI, female<br>± depleted in neutrophils (adults, 8 weeks)<br>(*n* = 4–5)	2.5 mg/mouse<br><br>Timepoints:<br>- 1, 3, 30, 120 days post IT	Crystalline:<br>- DQ-12 (quartz)	2.2 µm	- Cytotoxicity (LDH release)<br><br>- BAL analysis<br>- Histopathology<br>- Immunohistochemistry (p40 IL-12)<br>- ELISA (p40 and p70 IL-12, IFN-γ, IgG_1, IgG_{2a})	- Significant increase of LDH release at all timepoints (excepting 30 days post IT).<br>- Significant increase of protein content, total cell count, neutrophils and lymphocytes count at all timepoints.<br>- Observation of fibrotic nodules at 120 days post IT.<br>- Positive p40 IL-12 cells found exclusively in silicotic areas at 120 days post IT.<br>- Persistent increase of p40 IL-12 and IgG_1 at all timepoints.<br>Significant increase of IgG_{2a} only 30 days post IT.<br>No significant differences in p70 IL-12 and IFNγ.	Huaux, et al.<br>Am J Respir Cell Mol Biol<br>1999

Intratracheal instillation	Mice NMRI, female<br>± depleted in neutrophils<br>(Adults, 8 weeks)<br>(*n* = 4–5)	5 mg/mouse<br><br>Timepoints:<br>- 1, 3, 30 days post IT	Crystalline:<br>- DQ-12 (quartz)	2.2 µm	- Cytotoxicity (LDH release)<br>- BAL analysis<br>- Plasminogen Activator (PA) activity<br>- PCR (urokinase PA)<br>- Immunohistochemistry (uPA)	- Significant increase of LDH release 1 day post IT.<br>- Significant increase of total cell count 1 day post IT.<br>- Maximal and constant increase in early stages (1- and 3-days post IT) but lower upregulation at fibrotic stages (30 days post IT). PA activity almost totally abolished by amiloride (specific inhibitor of uPA form).<br>- Upregulation of uPA correlated with persistent increase in uPA mRNA levels in lung tissue at 3- and 30-days post IT.<br>- Positive staining in alveolar macrophages and pneumocytes 30 days post IT.<br>- No changes of uPA in mice with neutropenia suggesting that alveolar macrophages are the principal source of uPA response to silica	Lardot, et al. Am J Physiol 1998
Intratracheal instillation	Mice CD-1<br>(sex N/A)<br>(age N/A)<br>(*n* = 3)	0.2 mg/mouse<br>(3 days after ± bleomycin IT)<br><br>Timepoints:<br>- 3, 7 days or 12 weeks post IT	Crystalline:<br>- quartz<br>(Dowson and Dobson)	<3 µm	- BAL analysis<br>- Hydroxyproline content<br>- Histopathology:<br>. lung tissue<br>. lymph node	- Significant increase of protein levels and total cell count in mice exposed to bleomycin and silica 3 days post IT.<br>- Significant increase of hydroxyproline content in mice exposed to bleomycin and silica 12 weeks post IT.<br>- Silica particles translocated to the interstitium 7 days post IT.<br>Increased pulmonary fibrosis and retained-silica lung content in mice exposed to bleomycin and silica 12 weeks post IT.	Adamson and Prieditis, Exp Lung Res 1998

						Enlarged hilar lymph nodes with many granulomas-containing macrophages and silica in mice exposed to bleomycin and silica 12 weeks post IT.	
Intratracheal instillation	Rats Crl:CD BR, male (adult, 9 weeks) (*n* = 3)	10 mg/kg<br><br>Timepoints:<br>0.5, 2, 5-h, 2- or 10-days post exposure	Min-U-Sil (Pennsylvania Glass and Sand Corp)	1–4 µm	- BAL analysis<br>- Chemotaxis Assay<br>- RT-qPCR (MIP-2 and KC)	- Neutrophilic inflammation and maximal neutrophil recruitment after 5h and remaining prominent 10 days after exposure.<br>- Chemotactic activity for neutrophils detected directly in BAL within 2 h and remaining prominent 10 days after exposure.<br>- Neutrophil chemotactic cytokines expressed prior the detection of chemotactic activity, 0.5h after exposure, but no longer detectable 2 days post exposure.	Yuen, et al. Am J Respir Cell Mol Biol 1996
Intratracheal instillation	Rats Sprague Dawley (Sex N/A) (Age N/A) (*n* = 8)	50 mg/rat<br>Timepoint:<br>- 7 days post IT	Crystalline:<br>- Min-U-Sil 5 (Pittsburgh Glass and Sand Corp)	3.87 µm	- Cytotoxicity (LDH release)<br>- BAL analysis<br>- Production of ·OH in lungs	- Significant increase of LDH release.<br>- Significant increase of protein concentration, total cell count and neutrophils.<br>- Significant increase of ·OH production.	Schapira, et al. Am J Respir Cell Mol Biol 1995
Intratracheal instillation	Rats Sprague Dawley (Sex N/A) (Age N/A) (*n* = 9–25)	Dose 20 mg/0,5mL<br><br>Timepoint:<br>- 2 weeks post IT	Crystalline:<br>- Min-U-Sil (Pittsburgh Glass and Sand Corp) ± washed with HCl (remove contaminating Fe_2O_3)	<5 µm	- BAL analysis (phospholipids and protein content)<br>- Lung weight and lung microsomal protein concentration<br>- Measurement of xenobiotic	- Significant increase of phospholipids and protein content for both silica (effects exaggerated for silica washed with HCl).<br>- Significant increase of lung weight and lung microsomal protein concentration for both silicas (effects exaggerated for silica washed with HCl).<br>- Significant increase of reactions mediated by CYP4501A1 and 2B1 for	Miles, et al. Toxicol Appl Pharmacol 1994

					metabolism in lung microsomes<br>- Measurement of hydroxyl radicals and iron levels by electron spin resonance	silica washed with HCl only.<br>- Significant increase of hydroxyl radicals and iron levels for silica washed with HCl only.	
Intratracheal instillation	Mice 50 Swiss-Webster, male (Age N/A) (*n* = 4)	1 mg/mouse (unique)<br>± whole body irradiation to delay the inflammatory response and particle clearance<br><br>Timepoints:<br>- 0, 3 days, 1, 2, 4, 8, 12, 16 weeks post IT	Crystalline:<br>- crystalline quartz (Dowson and Dobson)	N/A	- White blood cell count<br>- BAL analysis<br>- Morphology<br>- Hydroxyproline content<br>- Tissue residue	- No significant difference for white blood cell count.<br>- Delayed increase of neutrophil and alveolar macrophages count in irradiation + silica group (2 weeks) compared to group exposed to silica alone (3 days).<br>Higher levels of protein content in irradiation + silica group overtime.<br>- Silica particles reached the interstitial macrophages in the first 2 weeks in irradiation + silica group compared to group exposed to silica alone.<br>Greatly increased fibroblast proliferation and deposition of collagen with large interstitial granulomas at the sites of silica retention in irradiation + silica<br>group compared to group exposed to silica alone.<br>More interstitial particles observed in irradiation + silica group.<br>- Significant and similar increase of hydroxyproline content from 2 to 16 weeks post IT.	Adamson, et al. Am J Pathol 1989

						- Significant increase of tissue residue (more important in irradiation + silica group) at 16 weeks post IT.<br>- Limiting initial inflammatory response is useful for increasing transepithelial passage of particles and their subsequent long-term retention in lung.<br>- Greater role of interstitial macrophages in fibrogenesis.	
Intratracheal instillation	Mice Swiss Webster, male (Age N/A) (*n* = 4)	2 mg/mouse (unique)<br><br>Timepoints:<br>- 1, 2, 3, 5, 7, 10 days post IT<br>- 2, 4, 6, 8, 10, 12, 16, 20 weeks post IT	Crystalline:<br>- crystalline quartz (Dowson and Dobson)	0.3 μm	- BAL analysis<br>- Lung morphology<br>- Biochemistry (total DNA and hydroxyproline content)	- Sustained increase of neutrophils and alveolar macrophages overtime but similar to control levels from 8 to 8 weeks post IT.<br>Increase of glucosaminidase and glucuronidase levels.<br>- Rapid neutrophils and alveolar macrophages recruitment to the alveoli. Silica found in interstitial macrophages.<br>- Increase DNA (from 2 days to 20 weeks) and hydroxyproline synthesis (from 4 weeks).	Adamson and Bowden. Am J Pathol 1984
Oro-pharyngeal aspiration	Mice C57BL6, male and female (adults, 9–12 weeks) (*n* = 4–12)	1 mg/mouse/exposure (unique or every week)<br><br>Timepoints:<br>- acute (24 h and 7 days post exposure)<br>- subchronic (4 weeks)	Crystalline:<br>- Min-U-Sil	from 1 to 2.5 μm for larger particles from 200 nm to 1 μm for smaller particles	- Flow cytometry<br>- LLF analysis<br>- Histological analysis	- Acute: no sex differences 24 h post exposure but greater airspace neutrophilia in female mice compared to males after 7 days.<br>- Significant increase of inflammatory cytokine levels (IFNγ, IL-10, IL-1β, IL-6, and TNFα) in female mice compared to males after 7 days.<br>- Subchronic: worse alveolitis and	Ray and Holian. Inhal Toxicol 2019

						greater dendritic cell presence within the lungs in male mice.	
Oro-pharyngeal aspiration	Mice C57BL6, male and female<br>- WT<br>- caspase-1 null<br>(Adults, 8–12 weeks)<br>(*n* = 4)	1 mg/mouse (unique or once a week during 4 weeks)<br><br>Timepoints:<br>- Acute: 7 days post exposure<br>- Chronic: 56 days post exposure	Crystalline:<br>- Min-U-Sil (Pennsylvania Glass Sand Corp)	N/A	- Cytotoxicity (LDH release)<br>- BAL analysis<br>- RT-PCR (NLRP3 inflammasome mRNA: Nlrp3, ASC, pro-IL-1β)<br>- Western Blot<br>- Immunofluorescences Detection of Cathepsins	- Significant increase of LDH release (acute).<br>- Significant increase of total cell count and total protein levels in mice (acute).<br>- Significant increase of NLRP3 inflammasome mRNA (acute).<br>- Significant increase of IL-1β, IL-18, HMGB1 and NLRP3 inflammasome proteins (acute).<br>- Greater extracellular cathepsins (L, B, V) and intracellular cathepsin B activity (acute).<br>- No effects in Mice C57BL6 caspase-1 null suggesting effects are dependent on inflammasome activation. Extracellular cathepsins could be the consequence of lysosome disruption and cell death.	Jessop, et al. Toxicol Appl Pharmacol 2017
Oro-pharyngeal aspiration	Mice C57BL/6 and Balb/c, female<br>(Age N/A)<br>(*n* = 4–6)	2.5 mg/mouse (unique)<br><br>Timepoints:<br>- 3 days, 1, 2 months post exposure	Crystalline:<br>- DQ-12 (quartz)	2.2 µm	- RT-qPCR (cathepsin transcripts: Cat K, S, L and B)<br>- Total TGF-β1 lung content<br>- Hydroxyproline content	- Cat K strongly upregulated at all timepoints.<br><br>- Significant increase of TGF-β1 only in C57BL/6 mice, 2 months post exposure. Cat K expression inversely related to the level of TGF-β1.<br>- Significant increase of hydroxyproline content at all timepoints.	Van Den Brûle, et al. Respir Res 2005
Oro-pharyngeal aspiration	Mice C57BL/6 (Sex N/A)<br>(Age N/A)	2.5 mg/mouse (unique)	Crystalline:<br>- DQ-12 (quartz)	2.2 µm	- BAL analysis<br>- Silica amount retained in the lung	- Significant increase of lymphocytes at both timepoints.	Barbarin, et al. Am J Lung Cell Mol Physiol

	(*n* = 5–6)	Timepoints:<br>IL-10 overexpression by adenoviral gene transfer<br>- 1 day before exposure<br>- 30 days after exposure			- Hydroxyproline content<br>- ELISA (fibronectin and type I collagen, IL-10, IL-4, IL-13, TGF-β, IgG1, IgG2a, PGE_2)	- No differences in silica content (compared with WT mice).<br>- Significant increase of hydroxyproline content.<br>- Significant increase of IgG1 (but not IgG2a), IL-4 and IL-13 (→ induction of a Th2-like immune response).<br>No differences for TGF-β and PGE_2 (compared with WT mice). - - IL-10 overexpression contributed to silica-induced lung fibrosis by exacerbation of Th2 response.	2005
Oro-pharyngeal aspiration	Mice C57BL6 iNOS KO or WT, male (adult, 8–10 weeks)<br>(*n* = 5)	40 mg/kg<br><br>Timepoints:<br>24 h and 42 days post exposure (acute and sub-chronic)	Min-U-Sil (U.S. Silica co)	<5 μm	- Cytotoxicity (LDH release)<br>- BAL analysis (total cell count and differential)<br>- ELISA (TNF- α, MIP-2, TAC)<br><br>- Zymosan-stimulated AM chemiluminescence<br>- Hydroxyproline content<br>- Histopathology	- Significant increase for iNOS KO and WT mice after silica exposure (acute and sub-chronic).<br>- Significant decrease of AM and increase of PMN for iNOS KO and WT mice after silica exposure (acute and sub-chronic).<br>- Significant increase of TNF-α and MIP-2 for iNOS KO and WT mice after silica exposure (acute and sub-chronic). Total Anti-oxidant Capacity decreased in WT mice but maintained in iNOS KO mice (compared to WT).<br>- Less AM activation for iNOS KO mice<br><br>- Less increase of hydroxyproline content for iNOS KO mice.<br>- Less lung damage for iNOS KO mice.	Zeidler, et al. Journal Toxicol Environ Health 2004

Intranasal instillation	Mice NZBWF1 female (Strain with robust autoimmune triggering by $cSiO_2$) (Adults, 8 weeks) (*n* = 40)	1 or 2.5 mg/mouse (unique)<br><br>Timepoints:<br>1, 7, 14, 21 and 28 days post intranasal instillation (acute and sub-chronic)	$cSiO_2$<br>Min-U-Sil 5 (Pennsylvania Sand Glass Corporation)	1.5–2 µm	- Cell death measurement (release of protein, dsDNS and LDH)<br>- ELISA assay (IL-1β, IL-1α, IL-6, TNF-α, IL-18, MCP-1 and BAFF)<br>- Lung histopathology, Immunohistochemistry and Morphometry<br>- NanoString Autoimmune Gene Profiling<br>- Gene Ontology and Network analysis	- Significant increase of cell death by 7 days post intranasal instillation.<br><br>- Significant secretion of cytokines and B cell activator (BAFF) by 7 days post intranasal instillation.<br><br>- Robust recruitment of macrophages, neutrophils and lymphocytes into the alveoli by 7 days post intranasal instillation<br><br>- Upregulation of genes associated with chemokines, proinflammatory cytokines, lymphocyte activation and type I interferon signaling by 7 days post intranasal instillation<br>- Ectopic lymphoid structure (ELS) development with emergence of organized $CD3^+$ T cells after 14 days and $CD45R^+$ B cells after 21 days post intranasal instillation.	Chauhan, et al. Frontiers Immunol 2021
Intranasal instillation	Mice C57BL6, male (Adults, 8–12 weeks) (*n* = 6)	25 µg/mouse<br><br>Timepoints:<br>5, 10, 60 and 120min post exposure (acute)	Min-U-Sil (U.S. Silica co)	1.6 µm	- Western Blot (NLRP3, γH2AX, pCHK2, ATX, Gprc5a, CC10)<br>- ELISA (ATX and CC10)	- Rapid DNA damage response with increased levels of γH2AX and pCHK2. Increase of Gprc5a indicating an involvement of respiratory epithelial cells.<br>- Increase of CC10 indicating an involvement of respiratory epithelial cells.	Wu, et al. Part Fibre Toxicol 2020
Trans nasal instillation	Mice C57BL6, male (adults, 6–8	8 mg/mouse (twice)	Crystalline (Japan Association for Working	N/A	- Immunohistochemical examination (Fas	- Fas and p62 expressed by histiocytes (macrophages) in granulomas after 7 and 56 days.	Shimizu, et al. J Clin Biochem Nutr

	weeks)<br>(n = 5)	Timepoints:<br>- 7 or 56 days	Environment Measurement)		and p62 protein expression)<br>- In-air micro-PIXE analysis of silica in lung tissue sections	- Silica particles co-localized in granulomas.	2015
Intranasal instillation	Mice C57BL6 and Balb/c, male and female<br>- WT<br>- KO (MARCO$^{-/-}$)<br>(Adults, 6–8 weeks)<br>(n = 5–6)<br><br>MARCO: macrophage receptor with collagenous structure	1 mg/mouse<br>(unique or chronic: once a week during 4 weeks)<br><br>Timepoints:<br>- Acute: 1, 3, 7, 14, 28 days post instillation<br>- Chronic: 3 months post instillation	Crystalline:<br>- Min-U-Sil (Pennsylvania Glass Sand Corp)	1.5–2 µm	- Uptake of silica<br>- Microarray analysis<br>- Flow cytometry<br>- ELISA (IL-1β, TNF-α, IL-6)<br>- Lung wet weight (chronic)<br>- Histopathological analysis (chronic): Gomori's trichrome staining	- Significant decrease of particle uptake in MARCO KO mice.<br>- Significant increase of mRNA MARCO after 7 and 14 days in WT mice.<br>- Significant increase of total cells, neutrophils, alveolar macrophages and dendritic cells in MARCO KO mice (only total cells and neutrophils in WT mice).<br>- Significant increase of cytokines, (IL-6 more important in MARCO KO mice).<br>- Significant increase of lung wet weight, more important in MARCO KO mice.<br>- Small difference of hydroxyproline content in both type of mice. Increased chronic inflammation in MARCO KO mice.<br>Increased chronic inflammation in MARCO KO mice.	Thakur, et al. Toxicol Sci 2009
Intranasal instillation	Mice C57BL6 ASC or Nalp3 KO or WT<br>(Sex N/A)<br>(Young, 6 to 8 weeks)	1 mg/mouse<br>(Twice: at day 0 and 14)<br>Timepoints:<br>- 3 months after the first exposure	Crystalline SiO_2	N/A	- Histopathology	- ASC and Nalp3 KO mice have decreased inflammation and collagen deposition in response to inhaled silica.	Cassel, et al. PNAS 2008

	(n = 5)						
Intranasal installation	Mice Balb/c (Sex N/A) (Age N/A) (n = 5)	1 mg/mouse (twice at day 0 and 14)<br><br>Timepoints:<br>- 3 days, 1, 2 months post exposure	Crystalline (acid-washed crystalline silica)	1.5–2 µm	- Flow cytometry (antigen presenting cell markers)	- Significant increase of classic APC markers (MCH class II and CD11c) in interstitial macrophages.	Migliaccio, et al. Toxicol Appl Pharmacol 2005

Table S2. In vivo studies, nanometric crystalline silica.

Exposure Route	Animal Model	Dose Duration	Silica Name and Supplier	Primary Particle Size	Tests	Main Results	Reference
Intratracheal instillation	Rats Crl:CD BR, male (Adult, 8 weeks) (n = 5)	1 or 5 mg/kg (unique)<br><br>Timepoints post-IT:<br>- 24 h, 1 week,<br>1 month (acute and sub-chronic)<br>- 3 months (chronic)	-Nanoquartz I<br>-Nanoquartz II<br>(Synthesized hydrothermally)	- 50 nm<br>- 12 nm	- Haemolytic potential<br>- Cytotoxicity (LDH release)<br>- Histopathology	- High haemolytic potential for Nanoquartz II.<br>- Significant increase of LDH release, higher for Nanoquartz II.<br><br>- Typical quartz-related effects dose-dependent lung inflammatory macrophage accumulation responses concomitant with early development of pulmonary fibrosis, higher for Nanoquartz II.	Warheit, et al. Toxicol Sci 2007

Table S3. In vivo studies, micrometric amorphous silica.

Exposure Route	Animal Model	Dose Duration	Silica Name and Supplier	Primary Particle Size	Tests	Main Results	Reference
Whole-body Inhalation	Rats CD (sex N/A) (age N/A) (n N/A)	- Zeofree 80:10 or 100 mg/m^3<br>(3 days)<br>Timepoints:<br>- 10, 20, 30, 40, 50, 60, 70, 80, 90 days post exposure<br><br>- Ludox colloidal	Amorphous:<br>- Zeofree 80<br>(JM Huber Co)<br>- Ludox colloidal silica<br>(DuPont Chemicals)	- 2.4–3.4 µm<br><br>- 2.9–3.7 µm	- Cytotoxicity (LDH release)<br>- BAL analysis (protein content and N-acetyl glucosaminidase)	Zeofree 80:<br>- Significant but transient increase of LDH release (similar to control from 10 to 90 days post exposure).<br>- Significant but transient increase of neutrophil recruitment and NAG (similar to control from 10 to 90 days post exposure).<br>Ludox colloidal:	Warheit, et al. Scand J Work Environ Health 1995

		silica: 10, 50 or 150 mg/m^3 (6 h/day, 5 days/week) Timepoints: - 2 or 4 weeks				Same transient inflammatory response at 50 and 150 mg/m^3.	
Intratracheal instillation and oro-pharyngeal aspiration	Mice C57BL6, female (adult, 7 weeks) (n = 7)	14 (IT), 43 or 128 µg/mouse<br><br>Timepoints: 24-, 72-h, 7- and 28-days post exposition (acute and sub-chronic)	Synthesized through wet-chemical procedures	0.3 µm ± porosity ± CuO doping	- BAL analysis<br>- Acute phase response<br><br>- Histopathology	- Increased accumulation of neutrophils for porous silica (128 µg)<br>- Greater induction of acute phase response for porous silica and CuO doping particles<br>- Milder inflammation in lung tissue at day 7 comparing with 100nm particles	Hadrup, et al. Nanotoxicology 2021
Intratracheal instillation	Mice C57BL6, female (adult, 8-10 weeks) (n = 11–14)	30 or 400 µg<br><br>Timepoints: 72 post IT (acute)	Amorphous silica microparticles (Micromod Partikeltechnologie GmbH)	3 µm	- BAL analysis<br><br>- Histological analysis<br>- RT-qPCR (MIP-1α, MIP2, TNF-α)<br>- ELISA (MIP-2)	- Less severity compared with NPs in terms of neutrophilic infiltration<br>- Less severity compared with NPs in terms of lung injury<br>- Significant increase of the expression of MIP-1α and TNF-α but not for MIP-2<br>- Attenuation of MIP-2 in comparison with NPs	Inoue, et al. Part Fibre Toxicol 2021
Intratracheal instillation	Mice C57BL6, sex N/A (young, 5–6 weeks) (n = 3–4)	25 mg/kg<br><br>Timepoints: 6 and 24 h post IT(acute)	Amorphous silica microparticles (Micromod Partikeltechnologie GmbH)	3 µm	- Flow cytometry<br>- ELISA (TNF-α, IL-1 β, IL-6)	- Neutrophil infiltration less important compared with 30nm.<br>- Significant increase of IL-6 only but less important compared with 30nm.	Kusaka, et al. PLOS One 2014
Intratracheal instillation	Mice C57BL6, female<br>- WT<br>- KO (IL-1$\alpha^{-/-}$ and IL-1$\beta^{-/-}$)<br>(adults, 8-12 weeks)	2.5 mg/mouse (unique)<br><br>Timepoints:<br>- 1, 3, 6, 12, 24 h post IT (acute)	Amorphous:<br>- VS (vitreous silica)	1.6 µm	- BAL analysis<br>- RT-qPCR<br>- ELISA	- Significant increase of total cell count and neutrophils.<br>- Significant increase of IL-1α 1h post IT (more important compared with DQ12 and Stöber silica).<br>Significant increase of IL-1β 24 h post IT (more important compared with DQ12).	Rabolli, et al. Part Fibre Toxicol 2014

	(*n* N/A)						
Intratracheal instillation	Rats Sprague Dawley, male (adults, 3 months) (*n* = 8)	30 µg (unique) ± media: saline, BSA, LLF<br><br>Timepoint:<br>- 24 h	Amorphous (± positive charge with NH_2 modification)	200 nm	- Cytotoxicity (LDH release)<br>- BAL analysis (albumin content)<br>- Nrf2 staining in BAL<br>- RT-PCR	- No significant difference in LDH release.<br>- No significant difference in total cell count and albumin content.<br>- No significant difference in Nrf2 staining.<br>- No significant differences in cytokine mRNa expression.	Brown, et al. Toxicol Lett 2013
Intratracheal instillation	Mice ICR, female (adults 10–12 weeks) (*n* = 3)	3 mg (unique)<br><br>Timepoints:<br>- 30min and 24 h post IT	Amorphous:<br>- fine colloidal silica (Fuso Chemical Co)	213 nm	- Histopathology<br>- Immunohistochemistry (laminin)<br><br>- Electron Microscopy	- Bronchiolar degeneration, necrosis, neutrophilic inflammation in alveoli with alveolar type II cell swelling and particle-laden alveolar macrophages accumulation at 3 days after instillation<br>- Positive immunolabelling.<br>- Presence of particles on bronchiolar and alveolar wall surface and cytoplasm of alveolar epithelial cells, macrophages and neutrophils	Kaewamatawong, et al. Toxicol Pathol 2005
Intratracheal instillation	Rats Crl:CD BR, male (Adult, 9 weeks) (*n* = 3)	10 mg/kg<br><br>Timepoints:<br>0.5-, 2-, 5-h, 2- or 10-days post exposure	Zeofree80 (JM Huber Corp)	2–3.5 µm	- BAL analysis<br><br>- Chemotaxis Assay<br><br>- RT-qPCR (MIP-2 and KC)	- Neutrophilic inflammation and maximal neutrophil recruitment 5h after exposure.<br>- Chemotactic activity for neutrophils detected directly in BAL within 2 h after exposure.<br>- Neutrophil chemotactic cytokines expressed prior the detection of chemotactic activity, 0.5h after exposure. but no longer detectable 2 days post exposure.	Yuen, et al. Am J Resp Cell Mol Biol 1996
Oro-pharyngeal aspiration	Mice C57BL6, male (adult, 8 weeks) (*n* = 6)	1.6 and 10 mg/kg<br><br>Timepoints:<br>6 and 40 h post exposition (acute)	Aerosil, commercial fumed silica (Sigma)	0.23 µm ± calcinated ± rehydrated ± Ti and Al doping	- BAL analysis<br>- Histopathology<br>- ELISA (IL-1β, MIP-1α, LIX)	- Significant increase of neutrophils for fumed silica.<br>- Focal inflammation induced by fumed silica.<br>- Significant increase of pro-inflammatory cytokines.	Sun, et al. ACS Nano 2015

						Those acute pro-inflammatory effects are attenuated by calcination (temporary), Ti and Al doping (stable) but exacerbated by rehydratation. Indeed, doping could reduce surface silanol density and expression of three-membered siloxane rings.	

Table S4. In vivo studies, nanometric amorphous silica.

Exposure Route	Animal Model	Dose Duration	Silica Name and Supplier	Primary Particle Size	Tests	Main Results	Reference
Whole-body Inhalation	Rats Crl:CD(SD)IGSBR, male (Adults, 9 weeks) (*n* = 25)	- 0, 10, 50 or 150 mg/m^3 (6 h/day, 5 days/week, 4 weeks)<br><br>Timepoint:<br>- at the end of the 4 weeks<br>- 10 days or 3 months post exposure	Amorphous<br>- Ludox colloidal silica (DuPont Chemicals)	22 nm	- Histopathology	- Dose-related pulmonary lesions observed at 50 and 150 mg/m^3.<br>Inhaled particles mostly phagocytized by alveolar macrophages and accumulated in BALT in peribronchiolar or perivascular aeras.<br>Few particles found in type I pneumocytes in the alveoli.<br>Silicotic granulomas initially formed in alveoli with particle-laden alveolar macrophages and proliferating epithelioid cells.<br>Collagen fiber deposition minimal in granulomas compared with those induced by crystalline silica.<br>Greater ability of ultrafine size to induce lung inflammation and tissue damages compared with fine size (cf table).	Lee and Kelly, Toxicology 1993
"Nose only" inhalation	Rats (from own breeding colony), female (age N/A) (*n* N/A)	2.6 and 10.6 mg/m^3 (4 h/ day, 5 times a week)<br><br>Timepoints:<br>- 3 and 6 months (chronic)	Amorphous<br>- NSCA (Nanosomoca containing aerosol)	90 nm	- Lung alterations<br>- SiO_2 content if organs, blood and excreta<br>- Genotoxicity (genomic DNA	- Significant and dose-dependent increase of relative mass of lungs and hydroxyproline content after 3 months of exposure (but not for 6 months).<br>- Significant increase of silica content in lungs but also in blood and excreta (lower concentration for 6 months).	Sutunkova, et al. Toxicology 2017

					fragmentation coefficient)	- Significant and dose-dependent increase of genomic DNA fragmentation coefficient.	
"Nose only" inhalation	Rats Brown Norway, female (adults, 8–9 weeks) (n = 6)	27 mg/m^3 (6 h/ day during 6 days)<br><br>Timepoint:<br>- 24 h after the last exposure (sensitization and challenge with TMA (Trimellitic anhydride))	Amorphous:<br>- fumed silica (pyrogenic) (Sigma-Aldrich)	14 nm	- Respiratory measurements<br>- Local effects, body and -Organ weight, necropsy and serum IgE levels<br>- Histopathology<br>- BAL analysis (cellular and biochemical changes)	- Transient changes in breathing parameters during silica exposure only. Irregular breathing pattern with TMA challenge.<br>- Highest increase of lung weight and tracheobronchial lymph node weight with TMA challenge.<br>- Reduction of mixed inflammation, ulceration and squamous metaplasia in larynx. Reduction of eosinophilic infiltration and oedema in lungs with TMA challenge.<br>- Increase of eosinophils and lymphocytes with TMA challenge.<br>Largest increase of LDH activity with TMA challenge.	Arts, et al. Inhal Toxicol 2008
Intratracheal instillation	Mice C57BL6, female (adult, 8–10 weeks) (n = 11–14)	30 or 400 µg<br><br>Timepoints:<br>24 and 72 post IT (acute)	Amorphous silica nanoparticles (Micromod Partikeltechnologie GmbH)	50 nm (without surface modification = plain or with amine surface modification: 50 nm–NH_2)	- BAL analysis<br>- Histological analysis<br>- RT-qPCR (MIP-1α, MIP-2, TNF-α)<br>- ELISA (MIP-2)	- Less severity for 50nm-NH2 in terms of neutrophilic infiltration<br>- Airway centered lung injury<br>- Significant increase of the expression of MIP-1α, MIP-2 and TNF-α<br><br>- Significant increase of MIP2 protein content	Inoue, et al. Part Fibre Toxicol 2021
Intratracheal instillation	Rats Sprague Dawley, male and female (young, 5 weeks) (n = 10)	12.5, 25 or 50 µg (3 times per week for 2 weeks)	Amorphous (Korea Research Institute of Standards and Science)	- 20 nm<br>- 50 nm	- Clinical pathology and organ weights<br>- Macroscopic and microscopic findings<br>- Bronchial alveolar lavage (BAL) fluid analysis<br>- Immunohistochemistry	- No dose-related differences in mortality, body weight gain or organ weight.<br>- Significant decrease of levels of hemoglobin and hematocrit for 20nm in female rats only (50 µg). Significant decrease of neutrophil proportion for both sizes of silica in male rats.<br>- Significant increase if total number of BAL cells for 20nm (50 µg).<br>- Significant increase expression of caveolin-1 and MMP-9 for 20nm in female rats.	Han, et al. Toxicol Appl Pharmacol 2020

Intratracheal instillation	Mice C57BL6 (Trm2$^{-/-}$ and WT), male (adult, 8 weeks) (n = 4–8)	10 mg/kg (unique) ± PJ34 treatment (PARP inhibitor) Timepoints: 7 days post IT (acute)	SiNPs (Sigma-Aldrich)	10–20 nm	- Cytotoxicity (LDH release) - BAL analysis - ELISA (TNF-α, IL-1β, IL-6)	- Significant increase of LDH release. - Significant increase of neutrophils and lymphocytes number. - Significant increase of pro-inflammatory cytokines. PJ34 treatment but also TRPM2 deficiency reduce SiNPs lung inflammation.	Wang, et al. Part Fibre Toxicol 2020
Intratracheal instillation	Mice BALB/c (WT and Nlrp$^{3-/-}$), female (adult, 6–10 weeks) (n = 4)	50 µg (unique) ± OVA for sensitization Timepoints: 5 days post IT (acute)	Amorphous silica nanoparticles (BASF SE)	50 nm ± PEGylated ± phosphonate ± amino	- RT-qPCR (cytokines: TNF-α, IL-1β, IL-6, IL-17a and chemokines: MCP-1, MIP-1α, MIP-2α, TARC, MDC, NAP-3) - TEM	- SiO_2 plain NPs induced NLRP3 inflammasome activation, increasing cytokines and chemokines. Effects of SiO_2 NPs attenuated by coating with phosphonate or amino groups while PEGylation enhanced it. - SiO_2 NPs were mostly localized in alveolar macrophages, within vesicles and/ or in phagolysosomes.	Marzaioli, et al. Nanomaterials 2017
Intratracheal instillation	Rats (from own breeding colony), female (age N/A) (n N/A)	7 mg/mL (unique) Timepoints: - 24 h post IT	Amorphous - NSCA (Nanosomoca containing aerosol)	90 nm	- Cytotoxicity in BAL	- Significant increase of total cells and neutrophils.	Sutunkova, et al. Toxicology 2017
Intratracheal instillation	Mice (strain, sex and age N/A) (n = 7)	0.02, 0.1, 0.2, 1, 2, 4 mg/mouse ± TGF-β1 Timepoints: - 20 or 56 days	Amorphous: -SiNPs (Kisker Biotech) (neutral or modified: . aminated N-SiNP . polymerated P-SiNP)	- 10 nm - 100 nm	- Histological score - Hydroxyproline content - Immunofluorescence analysis - Epithelial-Mesenchymal Transition (EMT)	- Significant increase of histological score for mice exposed to SiNP-100. - Significant increase of hydroxyproline content for mice exposed to SiNP-100 (less significant with N-SiNP or P-SiNP). - Colocalization of SiNP-100 and TGF-β1on the membrane. - Induction of EMT in mice exposed to SiNP-100 + TGF-β1. - Negative surface charge of SiNPs crucial to facilitate TGF-β1 binding.	Wang, et al. ACS Nano 2017

Intratracheal instillation	Mice BALB/c, female<br>(Adult, 8 weeks)<br><br>(*n* = 4)	- 7, 21 and 35 mg/kg<br>(repeated every 3 days, 5 times)<br><br>Timepoints:<br>14 days after first IT (acute)	Synthesized through the Stöber method	43 nm	- Histopathology<br>- Immunohistochemistry<br>- TEM<br><br>- Bead-based multiplex flow cytometry in serum (TNF-α, IL-1 β, IL-6, IL-18)	- SiNPs induced thick septa in the lung, capillary hyperemia and inflammatory cell infiltration with several consolidation areas.<br>- SiNPs were engulfed by the macrophages and internalized in lysosomes or pericytes<br>- Significant increase of inflammatory cytokines (dose-dependent for IL-1 β.)	Yang, et al.<br>Int J Nanomed<br>2016
Intratracheal instillation	Mice C57BL6, sex N/A<br>(Young, 5–6 weeks)<br>(*n* = 3–4)	25 mg/kg<br><br>Timepoints:<br>6 or 24 h post IT (acute)	Amorphous silica nanoparticles (Micromod Partikeltechnologie GmbH)	30 nm	- Flow cytometry (neutrophil infiltration)<br>- ELISA (TNF-α, IL-1 β, IL-6)	- Important neutrophil infiltration (higher than 3μm).<br><br>- Significant increase of all pro-inflammatory cytokines.	Kusaka, et al.<br>PLOS One<br>2014
Intratracheal instillation	Mice C57BL6, female<br>- WT<br>- KO (IL-1α$^{-/-}$ and IL-1β$^{-/-}$)<br>(Adults, 8–12 weeks)<br>(*n* N/A)	2.5 mg/mouse<br>(unique)<br><br>Timepoints:<br>- 1, 3, 6, 12, 24 h post IT (acute)	Amorphous:<br>- Stöber Silica<br>- fumed silica (Aerosil 200) (Sigma-Aldrich)	- 12 nm<br>- 12 nm	- BAL analysis<br>- RT-qPCR<br>- ELISA	- Significant increase of total cell count and neutrophils (more important for Aerosil 200).<br>- Significant increase of IL-1α 1 h post IT (more important for Aerosil 200).<br>Significant increase of IL-1β 24 h post IT (more important for Aerosil 200).	Rabolli, et al.<br>Part Fibre Toxicol<br>2014
Intratracheal instillation	Rats Sprague Dawley, male<br>(adults, 3 months)<br>(*n* = 8)	30 μg<br>(unique)<br><br>Timepoint:<br>- 24 h	Amorphous<br>(with neutral surface charge or positive NH_2 modification)	50 nm	- Cytotoxicity (LDH release)<br>- BAL analysis (albumin content)<br>- Nrf2 staining in BAL<br>- RT-PCR	- No significant difference in LDH release.<br>- Significant increase of neutrophils for both 50nm silica particles compared with 200 nm (except when dispersed in LLF).<br>No significant difference in albumin content.<br>- Significant increase in nuclear co-localization for Nrf2 staining for both 50 nm silica particles compared with 200 nm (when dispersed in saline).<br>- No significant differences in cytokine mRNa expression.	Brown, et al.<br>Toxicology Letters<br>2013

Intratracheal instillation	Rats Sprague-Dawley, male (Adult, 12 weeks) (*n* = 6)	600 μg/rat (unique)<br><br>Timepoints:<br>24 h, 7-or 30-days post IT (acute)	SiNPs (Degussa)	20 nm ± cadmium (Cd) doping	- F_2-IsoPs in lung<br>- Immunocytochemistry (SOD1, iNOS and COX-2)<br>- Histopathology<br><br>- Ultrastructural morphology	- F_2-IsoPs increased by 43% only in SiNPs-Cd group 30 days post IT.<br>- Immunoreactivity significantly enhanced in a time-dependent manner only in SiNPs-Cd group 30 days post IT.<br>- Patterns of injury with collapsed alveoli, thickening of alveolar wall and micro-hemorrhagic foci with bronchiolar deformation, epithelial cell desquamation and BALT formations<br>- High incidence of cytoplasmic phagosomes and residual bodies only in SiNPs-Cd group 30 days post IT.	Coccini, et al. Toxicology 2012
Intratracheal instillation	Mice A/J, male (Young, 5 weeks) (*n* = 5)	0, 2, 10 or 50 mg/kg<br><br>Timepoints:<br>-24 h, 1, 4 or 14 weeks	Amorphous:<br>- ultrafine amorphous silica (Sigma-Aldrich)	14 nm	- BAL analysis<br>- Histopathology and lung weights<br>- RT-qPCR<br>- Immunohistochemistry (IL-1β, IL-6, IL-8, TNF-α, MCP-1, MIP-2)	- Significant increase of total BAL cells.<br>- Severe neutrophilic inflammation after 24 h for all doses. Granulomatous inflammation after 1 and 4 weeks for 10 and 50 mg/kg. No effects after 14 weeks.<br>Significant increase of lung weights.<br>- Significant increase of mRNA and protein levels of IL-1β, IL-6, IL-8, TNF-α, MCP-1, MIP-2 after 24 h for 10 and 50 mg/kg but not after 1, 4 and 14 weeks.	Cho, et al. Toxicol Lett 2007
Intratracheal instillation	Mice ICR, male (adults, 7–8 weeks) (*n* = 7–10)	30 μg (unique)<br><br>Timepoints:<br>- 1, 3, 7, 15, 30 days post IT	Amorphous:<br>- ultrafine colloidal silica (Fuso Chemical Co)	14 nm	- BAL analysis<br>- Histopathology<br>- Immunohistochemistry (laminin and 8-OHdG)<br>- TUNEL assay	- Significant increase of total BAL cells, macrophages, neutrophils, lymphocytes and total protein at early timepoints (similar to control 30 days post IT).<br>- Alveolar macrophages and neutrophils infiltration into BALT (bronchus associated lymphoid tissue) of silica, 3 days post IT. Almost recovering of inflammatory lesions and lymph node except for slight thickening of alveolar septal, 30 days post IT.	Kaewamatawong, et al. Toxicol Pathol 2006

						- Discontinuous stain of laminin (suggesting pulmonary basement membrane destruction) at early timepoints. Positive stain 8-OHdG (linked to oxidative damage) at early timepoints.<br>- Significant increase of the apoptotic index in lung parenchyma at all timepoints.	
Intratracheal instillation	Mice ICR, female (adults 10–12 weeks) (n = 3)	3 mg (unique)<br><br>Timepoints:<br>- 30min and 24 h post IT	Amorphous:<br>- ultrafine colloidal silica (Fuso Chemical Co)	14 nm	- Histopathology<br>- Immunohistochemistry (laminin)<br>- Electron Microscopy	- Bronchiolar degeneration, necrosis, neutrophilic inflammation in alveoli with alveolar type II cell swelling and particle-laden alveolar macrophages accumulation.<br>+ extensive alveolar hemorrhage (compared with fine size).<br>- Positive immunolabelling but weaker compared with fine size.<br>- Presence of particles on bronchiolar and alveolar wall surface and cytoplasm of alveolar epithelial cells, macrophages and neutrophils.	Kaewamatawong, et al. Toxicol Pathol 2005
Oro-pharyngeal aspiration	Mice C57BL6, female (adult, 7 weeks) (n = 7)	14, 43 or 128 μg/mouse<br><br>Timepoints:<br>24-, 72-h, 7- and 28-days post exposition (acute and sub-chronic)	Synthesized through wet-chemical procedures	100 nm<br>± porosity<br>± CuO doping	- BAL analysis<br>- Acute phase response<br><br>- Histopathology	- Increased accumulation of neutrophils for porous silica<br>- Greater induction of acute phase response for porous silica and CuO doping particles<br>- Greater histological changes (perivascular and peribronchiolar) inflammatory cell infiltration by eosinophils and neutrophils in lung tissue) for porous silica (128 μg) after 24 h	Hadrup, et al. Nanotoxicology 2021
Intranasal instillation	Mice BALB/c, female (young, 6 weeks) (n = 6)	5, 10, 20 mg/kg (at day 21, 23 and 25)<br>+ OVA sensitization and challenge<br>Timepoints:<br>26 days post exposure (subchronic)	SiNPs (Sigma-Aldrich)	5–15 nm	- ELISA (IL-1 β, IL-5, IL-6, IL-13, TNF-α and IgE)<br>- Histopathology<br><br>- Immunohistochemistry and	- Significant increase of pro-inflammatory cytokine and IgE levels.<br><br>- Significant increase of Penh values, additional airway inflammation and mucus secretion.<br>- Significant increase of TXNIP, NLRP3 and IL-1β.	Ko, et al. Regul Toxicol Pharmacol 2020

				immunoblot<br>(TXNIP, NLRP3,<br>IL-1 β)		

Table S5. In vitro studies, micrometric crystalline silica.

Cell Line	Concentration and Duration	Silica Name and Supplier	Primary Particle Size	Tests	Main Results	Reference
A549 human adenocarcinoma alveolar basal epithelial cells	150 μg/mL<br>± miR-138 up-regulation<br>± siRNA transfection and gene silencing of ZEB2 (zinc finger E-box binding homeobox)<br>→ 24 h	S5631<br>(Sigma-Aldrich)	1–5 μm	- Western Blot<br>- Immunofluorescence<br><br>- Dual-luciferase reporter assay	- Up-regulation of miR-138 associated to a delayed silica-induced EMT process.<br>- Up-regulation of miR-138 lead to less fluorescence activity of α-SMA.<br>- Up-regulation of miR-138 reduced ZEB2 levels.	Wu, et al.<br>Toxicology<br>2021
- 16 hBE human bronchial epithelial cell<br>- A549 human adenocarcinoma alveolar basal epithelial cells	5 μg/cm²<br>± siRNA transfection and gene silencing of NLRP3<br><br>→3, 5, 10, 30, 60min	Min-U-Sil<br>(U.S. Silica co)	1.6 μm	- Comet assay<br><br>- ROS measurement<br><br>- Mitochondrial membrane potential measurement<br>- Western Blot<br>- ELISA	- Significant increase of DNA repair signaling but not in NLRP3 KO cells.<br>- No significant differences in mtROS production: DNA damage response not related to increased ROS levels.<br>- DNA damage response related to a NLRP3-dependent mitochondrial depolarization.<br>- Increased levels of γH2AX and pCHK2.<br>- Significant and rapid increase of NLRP3, Cas-1 and IL-1β: rapid NLRP3 activation by silica.	Wu, et al.<br>Part Fibre Toxicol<br>2020
- BEAS-2B human lung epithelial cells<br>- PBECs normal human primary bronchial epithelial cells	150X10⁶μm²/cm²<br>± inhibition or overexpression of SULF1<br><br>→ 24 and 48 h	Crystalline:<br>- cristobalite<br>(C&E Mineral Corp)<br>- DQ-12 (quartz)<br>(IUF Dusseldorf)	N/A	- Cell viability<br>- RT-qPCR analysis<br>- Immuno-fluorescence	- SULF1 inhibition: protection of cell viability.<br>SULF1 overexpression: exacerbation of loss of BEAS-2B cells viability with cristobalite.<br>- Down-regulation of SULF1 for both silica after 24 and 48 h in BEAS-2B cells.	Perkins, et al.<br>Toxicol Appl Pharmacol<br>2018

					+ SULF1 overexpression: significant increase of proliferative (CCND1, JUN, VEGFA and BIRC3) and fibrogenic (COLIA1, PAI1, ACTA2, Collagen) gene expression with cristobalite.<br>- Increase in sulphated-HSPGs expression for both silica in BEAS-2B cells.<br>- Potential role of SULF1 in silica-induced proliferative and fibrogenic signaling that could lead to epithelial hyperplasia, fibroblast recruitment and deposition of extracellular matrix.	
- BEAS-2B human lung epithelial cells<br>- A549 human adenocarcinoma alveolar basal epithelial cells	0, 12.5, 25, 50, 100 or 200 μg/cm²<br>(± TGF-β inhibitor)<br>→ 24 h	Crystalline:<br>- DQ-12PM1<br>(Institute for Occupational Safety and Health of German Social Accident Insurance)	<1 μm	- ELISA analysis (MMP-2, MMP-9, Col-1, Col-3: ECM-related markers)<br>- Western Blot analysis (E-cadherin and ZO-1: epithelial markers, α-SMA and vimentin: mesenchymal markers)<br>- RT-PCR analysis	- Significant increase of ECM-related markers.<br>- Significant loss expression of epithelial markers with significant increase expression of mesenchymal markers.<br>- Changes restricted with TGF-β inhibitor.	Rong, et al. Environ Toxicol Pharmacol 2015
BEC human bronchial epithelial cells	200 μg/mL<br>→ 0, 1, 6, 18, 24 h	Crystalline<br>(Sigma-Aldrich)	0.1–10 μm	- Western blot (Snail, E-cadherin: epithelial marker, α-SMA and vimentin: mesenchymal markers)<br>- EMSA (electrophoretic mobility shift assay)<br>- siRNA transfection (knock down of Snail expression)	- Significant increase expression of Snail (transcription factor downregulating the expression of E-cadherin and up-regulates the expression of vimentin).<br>- Significant increase of DNA binding activity of Snail.<br>- Inhibition of silica-induced expression of Snail. Up-regulation of E-cadherin and attenuation of α-SMA and vimentin in silica-stimulated cells.	Hu, et al. Biomed Environ Sci 2015
BEAS-2B human lung epithelial cells	0, 75, 150 or 300 × 10⁶ μm²/cm²	Crystalline:<br>- cristobalite (C&E Mineral Corp)	N/A	- Caspase-1 activity assay	- Caspase-1 activity attenuated with TRX addition.	Peeters, et al. Part Fibre Toxicol

	(± TRX treatment (thioredoxin, linked oxidative stress with inflammasome activation))<br>→ 24 h	- DQ-12 (quartz)<br>(IUF Dusseldorf)<br>± PVNO		- ELISA (IL-1β, bFGF, HMGB1)	- Secretion of IL-1β, bFGF, HMGB1 in a surface reactivity dependent manner (not when PVNO added).<br>- Hypothesis of protective role of TRX	2014
- BEAS-2B human lung epithelial cells<br>- NHBE human bronchial epithelial cells	15, 75, 150 or 300 × 10^6 $\mu m^2/cm^2$<br>→24 h	Crystalline:<br>- cristobalite<br>(C&E Mineral Corp)	1–4 μm	- Caspase-1 activity assay<br>- RT-qPCR<br>- Western Blot<br>- ELISA<br>- siRNA transfection (knock down of NLRP3)<br>- MRC-5 cells (fibroblasts) proliferation assay	- Significant increase of enzymatic activity of caspase-1.<br>- Significant increase of mRNA levels of caspase-1 and NLRP3.<br>- Significant increase of HMGB1 and IL-1β.<br>- Significant decrease of alarmins bFGF and HMGB1 in cells transfected with siNLRP3.<br>- No fibroblast proliferation in cells transfected with siNLRP3.	Peeters, et al. Part Fibre Toxicol 2013
- A549 human adenocarcinoma alveolar basal epithelial cells<br>- HIVE-26 endothelial cells<br>(± co-cultured)	80 and 160 $\mu g/cm^2$<br>(± inhibition of TNF-α and IL-1β receptors)<br>→43h	Crystalline:<br>- Min-U-Sil<br>(U.S. Silica co)	1.6 μm	- ELISA (TNF-α, IL-1α, IL-1β, IL-8, PTX3, FGF-2)<br>- Number of monocytes and pneumocytes	- Significant increase of IL-8 in A549 cells alone (both concentrations).<br>Significant increase of FGF-2 in A549 cells only (160 $\mu g/cm^2$). IL-8 and FGF-2 release suppressed in co-culture.<br>+ inhibition of IL-1β receptor: significant decrease of IL-8 in co-culture.<br>+ inhibition of IL-1β receptor: decrease number of pneumocytes correlated with increase of FGF-2 release in co-culture.	Herseth, et al. Part Fibre Toxicol 2008
- A549 human adenocarcinoma alveolar basal epithelial cells<br>- Primary rat T2 type-II epithelial cells	60 $\mu g/cm^2$<br>(± inhibition of EGFR)<br>→ 1 and 4 h	Crystalline:<br>- Min-U-Sil<br>(U.S. Silica co)	1.6–1.7 μm	- Chemokine measurements<br>- Immunoblotting<br>- Scanning electron microscopy	- +EGFR inhibitor: attenuation of IL-8 releases and of phosphorylation of SFK and ERK1/2.<br>- Increase of SFK and p38 phosphorylation after immediate silica exposure. Increase of ERK1/2 phosphorylation 10min after silica exposure	Øvrevik, et al. Toxicology 2006

					- Silica particles phagocytosed between 1 and 4 h of exposure but the majority remain bound by microvilli on the cell surface.	
- A549 human adenocarcinoma alveolar basal epithelial cells<br>- Primary rat T2 type-II epithelial cells	20, 40, 60, 80 μg/cm²<br><br>→ 15min, 30min, 1, 2, 4 and 24 h	Crystalline:<br>- Min-U-Sil (U.S. Silica co)	1.6 μm	- ELISA (IL-8, MIP-2)<br>- Immunoblotting	- Significant, concentration and time-dependent increase of IL-8 in A549 cells. Significant increase of MIP-2 in primary T2 cells.<br>- Phosphorylation of the MAPKs p38 and ERK1/2 (but not JNK or ERK5) and of the SFKs.	Øvrevik, et al. Toxicol Sci 2004
C10 murine non tumorigenic alveolar type II epithelial cells	10 or 20 μg/cm²<br><br>→ 8 and 24 h	Crystalline:<br>- DQ-12 (quartz)	960 nm	- Flow cytometry<br>- Western Blot<br>- Ribonuclease protection assay	- Significant increase of hypodiploid cells and cells in S phase.<br>- Phosphorylation of extracellular signal regulated kinases ERK1/2.<br>- Significant increase of *fos* and *jun* family members.	Albercht, et al. Toxicol Appl Pharmacol 2002
BECs human bronchial epithelial cells	10 or 50 μg/mL<br>± directly in cells or in culture medium with 10% of human serum<br><br>→ 15 min	DQ12 (Dorentruper Sand und Thonwerke GmbH of Dorentrup)	<0.5 μm	- Intracellular H_2O_2<br><br>- Intracellular ROS levels<br><br>- Enzyme inhibitory studies<br><br>- Spectrofluorometric experiments	- Significant increase of intracellular H_2O_2 after 10 μg/mL of DQ12 treatment in culture medium.<br>- Significant and identical increase of ROS levels in cells directly exposed to DQ12 or through DQ12-treated medium.<br>- Increased generation of intracellular ROS appears to involve both mitochondrial respiration and NAD(P)H oxidase-like system.<br>- Superoxide anions (O_2^{-}) and H_2O_2 are generated in DQ12-treated medium.	Deshpande, et al. Toxicol Sci 2002
16 hBE human airway epithelial cells	50 μg/mL<br><br>→24 h	Crystalline (microcrystalline silica)	1–5 μm	- RT-PCR (IL-1α, IL-1β, IL-6)<br>- Northern Blot (bFGF, TGF-β1)<br>- ELISA (IL-1α, IL-1β, IL-	- No significant differences.<br>- Significant increase of mRNA bFGF (basic fibroblast growth factor).<br>- Slight increase of IL-6 without affecting IL-1 and TGF-β isoforms.	Bodo, et al. Mol Med 2001

				6, bFGF, TGF-β1)<br>- Total proteins, collagen and fibronectin synthesis analysis	Significant increase of bFGF protein secretion.<br>- Significant production of total proteins, collagen and fibronectin production.	
A549 human adenocarcinoma alveolar basal epithelial cells	20, 40, 60, 80, 100 μg/cm^2<br><br>→40 h	Crystalline:<br>- Norquartz-45 (Glamsland)	<10 μm (PM_{10})<br><2 μm (PM_2)<br><0.5 μm ($PM_{0.5}$)	- Cytotoxicity (LDH release)<br>- ELISA (IL-6, IL-8)	- Significant increase of LDH release from 40 to 100 μg/cm^2 (except for PM_{10} at 40 μg/cm^2).<br>- Significant increase of IL-6 and IL-8 ($PM_{0.5}$ are more potent to induce IL-8).	Hetland, et al.<br>Hum Exp Toxicol<br>2001
MLE15 murine alveolar epithelial type II cells	50, 100, 200, 400 μg/mL<br>± serum in culture media<br><br>→6 h	Crystalline:<br>- Cristobalite	1.2 μm	- Cytotoxicity (LDH release)<br>- RNase protection assay<br><br>- Particle binding experiments	- Shift of dose-response curve when serum is added.<br>- Significant decrease of silica-induced chemokine response (MCP-1 and MIP-2 mRNA) when serum is added in MLE15 cells.<br>- Apolipoprotein-A1 identified in serum binds selectively to silica.	Barrett, et al.<br>Toxicol Appl Pharmacol<br>1999
AE6 rat lung alveolar epithelial cells	5, 50 μg/mL<br><br>→ 24, 48, 72 h	Crystalline:<br>- Cristobalite (C&E Mineral Corp): unheated CRIS<br>. heated: CRIS-, 800°C, -1300°C	1–20 μm (higher percentage of particles in 10–20 μm range for CRIS-1300)	- Cytotoxicity:<br>. Colony forming efficiency (CFE)	- Significant reduction of CFE for CRIS-800 and complete inactivation for CRIS-1300 in AE6 cells.<br>- CRIS-800 and 1300: hydrophobic confirming crucial role played by the hydrophilic patches of silanols on the silica surface for cytotoxic effects.	Fubini, et al.<br>Chem Res Toxicol<br>1999
Rats alveolar type II epithelial cells	100, 200, 300, 400, 500 μg/mL<br><br>→ 2, 4, 6, 8 h	Crystalline:<br>- Min-U-Sil (Pennsylvania Glass Sand Corp)	N/A	- Permeability<br>- Electrical resistance<br>- Cytotoxicity (LDH release)<br>- Electron microscopy	- Significant, dose and time-dependent increase of permeability except when silica was pre-treated with serum, charged dextrans or aluminium sulfate.<br>- Significant decrease on electrical resistance in cells exposed to 500 μg/mL of silica after 4 h.<br>- No significant difference in LDH release in cells exposed to 500 μg/mL of silica after 4 h.	Merchant, et al.<br>J Appl Physiol<br>1990

					- Adherence of the silica to the surface of alveolar epithelial cells.	
- NR8383 rat alveolar macrophages<br>- RAW 264.7 mouse monocyte-macrophage	50 µg/mL<br>± NAC (N-acetylcysteine)<br>→ 20 h	Crystalline	0.5–10 µm	- Immunofluorescence<br>- Western Blot	- Significant LC3 expression in macrophages.<br>- PI3K/Akt/mTOR signaling pathway is involved in the autophagy changed mediated by silica.<br>NAC can reduce the inflammatory response on NR8383 cells.	Li, et al. Environ Toxicol 2021
- BMDC murine bone marrow-derived dendritic cells<br>- BMDM murine bone marrow-derived macrophages	250 µg/mL<br>± DNase I treatment (degrade extracellular dsDNA)<br><br>→ 18 h	Crystalline:<br>- DQ-12 (quartz)	2.2 µm	- Measurement of double-stranded DNA<br>- Quantitative RT-qPCR analysis<br>- Quantification of mitochondrial versus nuclear DNA<br>- Immunofluorescence (MitoSOX)<br>- Flow cytometry<br>- Immunoblots:<br>. cleaved caspase-3 (apoptosis)<br>. cleaved gasdermin D (pyroptosis)<br>. phosphorylation of mixed lineage kinase domain-like pseudokinase (MLKL, necroptosis)	- Release of self-dsDNA, activating STING (stimulator of interferon genes) pathway in both type of cells.<br>+ DNase I: no more release of self-dsDNA.<br>- + DNase I: reduction of pro-inflammatory response (CXCL10 and Ifnα/β) in macrophages but not in dendritic cells.<br>- mt-DNA is the main source of DAMP activating STING pathway in dendritic cells.<br>- Significant increase of ROS production in both type of cells.<br>In macrophages, ROS are co-localized in mitochondria.<br>- Significant increase of cell death in both type of cells but also apoptosis in dendritic cells.<br>- Significant caspase-3 cleavage in both type of cells.<br>Phosphorylation of MLKL in macrophages only.<br>-DNase I: potential therapy for silica-induced lung inflammation?	Benmerzoug, et al. Nat Commun 2018

U937 human lymphocytes differentiate into macrophages	2.5 μg/cm^2<br>→0.5, 2, 8,16 and 24 h	Crystalline:<br>- Quartz silica powder<br>(Sigma-Aldrich)	0.5–10 μm	- RNA-seq<br>- qPCR<br>- ELISA<br>- Western blot<br>- Inhibition of ATF3 by siRNA	- Upregulation of ATF3 during silica exposure.<br>- Significant further increase of IL-1β, IL-6 and TNFα.	Chan, et al. Toxicology 2018
BMdM mouse (bone marrow derived macrophages)	0, 12.5, 25, 50 μg/mL<br>± bafilomycin A1 (inhibits lysosome acidification)<br><br>→4 h	Crystalline:<br>- Min-U-Sil<br>(Pennsylvania Glass Sand Corp)	N/A	- LMP (Lysosome membrane permeabilization) assay<br>- NLRP3 inflammasome activity: IL-1β and IL-18	- Significant increase of LMP in cells exposed to 50 μg/mL.<br>+ bafilomycin A1: decrease of LMP.<br>- Significant increase of IL-1β and IL-18 in cells exposed to 50 μg/mL.<br>+ bafilomycin A1: decrease of cytokines.<br>- Lysosome acidification prerequisite for particle-induced LMP and activation of NLRP3 inflammasome.	Jessop, et al. Toxicol Appl Pharmacol 2017
RAW 264.7 mouse monocyte-macrophage	16 μg/cm^2<br>→ 1, 2 or 4 h (TEM and live-cell analysis)<br>3h (ROS)<br><br>50 μg/cm^2<br>→ 2, 4, 8 h (cytokines analysis)	Crystalline<br>- SiO_2 quartz<br>(Nano-structured & Amorphous Materials)	- 4.1 μm<br>- 2.1 μm<br>- 700 nm<br>- 300 nm	- TEM analysis<br>- Live-cell analysis<br>- Cytokine analysis (20-plex)<br>- ROS measurements	- Differences in formation of subcellular organelles based on particle size.<br>- Differences in particle uptake based on particle size (faster for 300 nm).<br>- Significant increase of TNF-α for the smallest particles<br>(300 nm).<br>- Significant increase of ROS for the smallest particles<br>(300 nm).	Mischler, et al.<br>J Occup Med Toxicol<br>2016
THP-1 human macrophage-like cells	0, 75, 150 or 300X10^6 μm^2/cm^2<br>(± TRX treatment (thioredoxin, linked oxidative stress with inflammasome activation))<br>→ 24 h	Crystalline:<br>- cristobalite (C&E Mineral Corp)<br>- DQ-12 (quartz)<br>(IUF Dusseldorf)<br>± PVNO	N/A	- Caspase-1 activity assay<br><br>- ELISA (IL-1β, bFGF, HMGB1)	- Caspase-1 activity attenuated with TRX addition.<br>- Secretion of IL-1β, bFGF, HMGB1 in a surface reactivity dependent manner (not when PVNO added).<br>- Hypothesis of protective role of TRX	Peeters, et al. Part Fibre Toxicol<br>2014
- Mouse (*ex vivo*, from C57BL/6) alveolar macrophages	1.25, 2.5, 5, 10, 20, 40 μg/well<br><br>→ 4 h and 24 h	Crystalline:<br>- DQ-12 (quartz)	960 nm	- ELISA (pro-IL-1β or IL-1α)<br>- Western Blot	- Production of pro-IL-1β significantly induced by recombinant IL-1α but not IL-33 in culture of mouse alveolar	Rabolli, et al.

- J774 mouse monocyte macrophage cells		(DMT GmbH and Co)			macrophages.<br>- IL-1α released by J774 cells after silica exposure correlated with the degree of lung inflammation induced in vivo (cf table).	Particle and Fibre Toxicology 2014
MH-S mouse alveolar macrophages	50 μg/cm²<br><br>→ kinetic	Crystalline:<br>- Min-U-Sil<br>(U.S. Silica co)	N/A	- Time-lapse imaging of physiological indicators of cell death	- Phagolysosomal leakage 30 to 120 min after particle uptake.<br>- Cell blebbing, increase in mitochondria transmembrane potential and caspase-3 and -9 activation occurred at the same time between 3 and 6 h later.<br>- Possible secondary necrosis with swelling and decrease in mitochondria transmembrane potential.	Joshi and Knecht, Apoptosis 2013
- RAW 264.7 mouse monocyte-macrophage<br>- Rat BAL macrophages	100 μg/mL<br>± siRNA transfection and gene silencing for LANCL2 in RAW cells<br>± anti-ABA (abscisic acid) in RAW cells<br><br>→ 5, 10, 15, 30 and 60 min	Crystalline:<br>- Quartz (Min-U-Sil 5)<br>(US Silica)	N/A	- ABA release from each cell type<br>- Lipid peroxidation analysis<br><br>- $[Ca^{2+}]$i measurements<br>- NF-κB nuclear translocation<br><br>- COX-2 expression, PGE_2 and TNF-α production	- Significant increase of ABA release (earlier for alveolar macrophages).<br>- Activation of the plasma membrane receptor LANCL2 and lipid peroxidation through NADPH oxidase (NOX).<br>- Influx of extracellular Ca^{2+} and intracellular Ca^{2+} release.<br>- NF-κB nuclear translocation (inhibition in RAW cells treated with LANCL2 silencing or anti-ABA).<br>- PGE_2 and TNF-α release (inhibition in RAW cells treated with LANCL2 silencing or anti-ABA).	Magnone, et al.<br>FASEB J 2012
- RAW 264.7 mouse monocyte-macrophage<br>- Primary rat lung macrophages	- 10, 25, 50,100, 200 μg/mL<br>(± LPS treatment)<br><br>→ 6 h	Crystalline:<br>- Min-U-Sil<br>(U.S. Silica co)	N/A	- siRNA transfection (knock down of NLRP3)<br>- ELISA (IL-1β)<br>- RT-qPCR<br>- Western Blot	- Reduction of particle-induced IL-1β release in RAW264.7 cells.<br>- Significant and more potent increase of IL-1β compared with amorphous particles Si50 and Si500.	Sandberg, et al.<br>Part Fibre Toxicol 2012

					- Rat primary macrophage more sensitive to silica exposure compared with RAW264.7.	
- MH-S mouse alveolar macrophages<br>- RAW 264.7 mouse monocyte-macrophage	50 μg/cm^2<br>→ 4, 8, 12, 24 h	Crystalline:<br>- Min-U-Sil<br>(Pennsylvania Glass Sand Corp)	1.6 μm	- Cytotoxicity (propidium iodide)<br>- Western Blot<br>- Endosome-Phagosome fusion and endo-lysosomal leakage	- Significant increase of cell death in MH-S cells.<br>- Activation of the apoptotic pathway for 3 μm in MH-S cells.<br>- Endolysosomal leakage in MH-S cells.	Costantini, et al.<br>PLoS One<br>2011
- Primed macrophages from ASC or Nalp3 KO or WT C57BL6 mice<br>- Human alveolar macrophages	25, 50, 100 or 300 μg/cm^2<br>→ 4, 8, 12, 16, 20 or 24 h	Crystalline SiO_2	N/A	- ELISA (TNF-α, IL-6)<br>- RT-PCR (IL-1β, IL-12p40)<br>- Cytotoxicity (LDH release)<br>- ROS measurement<br>- Inhibition of Potassium Efflux	- Poor induction of TNF-α and IL-6 comparing with LPS treatment.<br>- Significant induction of IL-1β in a Nalp3-dependent manner (no induction in macrophages from Nalp3 KO mice).<br>- Significant increase of LDH release independent of Nalp3.<br>- Silica-induced ROS are required for Nalp3 inflammasome activation.<br>- Silica-induced IL-1 β secretion requires crystal internalization and potassium efflux.	Cassel, et al.<br>PNAS<br>2008
- MH-S mouse alveolar macrophages<br>- RAW 264.7 mouse monocyte-macrophage	5, 15, 25, 50, 100 μg/cm^2<br>→ 4 and 10 h	Crystalline:<br>- Min-U-Sil<br>(U.S. Silica co)	N/A	- Live cell particle binding assay<br>- Confocal particle imaging assay<br>- Electron microscopy<br>- Quantitative phagocytosis assay<br>- Cytotoxicity (propidium iodide)	- Macrophages bind silica particles and extend protrusions to capture distant particles.<br>- Silica particles present within the cytoplasmic volume of live cells.<br>- Silica particles present in internal cellular sections.<br>- Silica particles phagocytosed by macrophages.<br>- Silica induced cell-death in a time and concentration-dependent manner. Significant decrease of cell-death with antibody coated silica particles.	Gilberti, et al.<br>Am J Respir Cell Mol Biol<br>2008

- THP-1 human macrophage-like cells<br>- HIVE-26 endothelial cells<br>(± co-cultured)	80 and 160 μg/cm²<br>(± inhibition of TNF-α and IL-1β receptors)<br>→43h	Crystalline:<br>- Min-U-Sil<br>(U.S. Silica co)	1.6 μm	- ELISA (TNF-α, IL-1α, IL-1β, IL-8, PTX3, FGF-2)	- Significant increase of IL-8 in THP-1 cells alone (both concentrations).	Herseth, et al.<br>Part Fibre Toxicol<br>2008
- RAW 264.7 mouse monocyte-macrophage<br>- Rat BAL macrophages	15, 50 or 100 μg/mL<br>→ 6 or 18 h	Quartz (Min-U-Sil 5)<br>(US Silica)	N/A<br>± ascorbic acid pretreatment	- COX-2 expression and PGE_2 production<br>- RT-qPCR<br>- EMSA (Electrophoretic Mobility Shift Assay)<br>- Scavenger treatment	- COX-2 expression and PGE_2 production enhanced with quartz + ascorbic acid pretreatment.<br>- COX-2 mRNA threefold increase in cells incubated with quartz + ascorbic acid pretreatment.<br>- NF-κB, pCREB and AP-1 all implicated in the increased inflammatory response.<br>- ROS (H_2O_2 and $OH^{\cdot}$) involved in COX-2 expression.	Scarfi, et al.<br>The FEBS Journal<br>2006
- Mouse alveolar macrophages (*ex vivo*, from BAL of Balb/c)<br>- BMDM murine bone marrow-derived macrophages	0.2 mg/mL<br>→ 4 h	Crystalline<br>(acid-washed crystalline silica)	N/A	- Antigen presenting cell (APC) assay<br>- ELISA (IFNγ, IL-13)	- Significant increase of APC activity in alveolar macrophages.<br>- Significant increase of both cytokines.	Migliaccio, et al.<br>Toxicol Appl Pharmacol<br>2005
RAW 264.7 mouse monocyte-macrophage	50, 100, 200, 400 μg/mL<br>± serum in culture media<br>→6 h	Crystalline:<br>- Cristobalite	1.2 μm	- Cytotoxicity (LDH release)<br>- Particle binding experiments	- Shift of dose-response curve when serum is added.<br>- Apolipoprotein-A1 identified in serum binds selectively to silica.	Barrett, et al.<br>Toxicol Appl Pharmacol<br>1999
J774 mouse monocyte macrophage cells	5, 50 μg/mL<br>→ 24, 48, 72 h	Crystalline:<br>- Cristobalite<br>(C&E Mineral Corp): unheated CRIS<br>. heated: CRIS-, 800°C, -1300°C	1–20 μm<br>(higher percentage of particles in 10–20 μm range for CRIS-1300)	- Cytotoxicity:<br>. LDH release	- Significant increase of LDH release for CRIS but not for CRIS-1300 (substantially inert) in J774 cells.<br>- CRIS-800 and 1300: hydrophobic confirming crucial role played by the hydrophilic patches of silanols on the silica surface for cytotoxic effects.	Fubini, et al.<br>Chem Res Toxicol<br>1999
Mouse resident alveolar macrophages (*ex vivo*, from BAL of NMRI)	→ 18 h	Crystalline:<br>- DQ-12 (quartz)	2.2 μm	- PCR (*ex vivo* release of p40 and p70 IL-12)	- Rapid and marked overexpression of p40 subunit mRNA, regulation down-regulated as fibrosis progressed (similar	Huaux, et al.<br>Am J Respir Cell Mol Biol

					to control in BAL cultured 120 days post IT)	1999
BAM bovine alveolar macrophages cells	200 µg/mL<br><br>→ <10min	Crystalline:<br>- Sikron F600 (quartz powder)	<5 µm	- Flow cytometry:<br>. cytosolic free calcium concentration $[Ca^{2+}]i$<br>. cytosolic pH (pHi)<br>. plasma membrane potential (PMP)	- Significant increase of cytosolic free calcium concentration $[Ca^{2+}]i$.<br><br>- Significant decrease in pHi.<br>- significant increase of PMP.	Tarnok, et al. Anal Cell Pathol 1997
Rat alveolar macrophages (from BAL of Sprague-Dawley)	0.1, 1, 10 mg/mL<br>± 27µM of lipid surfactant (DPL) or surfactant preparation Survanta<br><br>→ 24 and 72 h	Crystalline:<br>- Min-U-Sil	N/A	- Eicosanoid levels by specific radioimmunoassay (RIA):<br>. Prostaglandin E_2 (PGE_2)<br>. Thromboxane B_2 (TxB_2)<br>. Leukotriene B_4 (LTB_4)	- Significant increase of PGE_2 for all concentrations in cells treated during 24 h and 72 h with silica + surfactants<br>- Significant decrease of TxB_2 for all concentrations in cells treated during 24 h with silica + surfactants compared with silica only.<br>- Significant decrease of LTB_4 for all concentrations in cells treated during 24 h with silica + surfactants. But significant increase in cells treated during 72 h.	Khun and Demers, Am J Physiol 1995
Human alveolar macrophages culture (from BAL of healthy donors)	0, 60, 100 µg/mL<br><br>→ 3 and 24 h	Crystalline:<br>- Min-U-Sil (Pennsylvania Glass Sand Corp)	2.2 µm	- HPLC analysis of eicosanoid production<br>- Eicosanoid levels by specific radioimmunoassay (RIA):<br>. Prostaglandin E_2 (PGE_2)<br>. Thromboxane B_2 (TxB_2)<br>. Leukotriene B_4, C_4, D_4, E_4 (LTB_4, C_4, D_4, E_4)<br>. 5-hydroxyeicosatetraenoic acid (5-HETE)	- Release of a variety of metabolites of both cyclooxygenase and lipoxygenase pathways.<br>- Significant decrease of PGE_2 and TxB_2 after 24 h for both concentrations.<br>-Significant increase of LTB_4, C_4, D_4, E_4 after 3h for both concentrations.<br>-Significant increase of 5-HETE after 3 and 24 h for both concentrations.	Koren, et al. Environ Health Perspects 1992
Rat alveolar macrophages (from BAL of Fischer 344)	50, 100, 200, 400 µg/2X10^6 cells<br><br>→ 24 h	Crystalline:<br>- Respirable silica particles	N/A	- Cytotoxicity (LDH release)<br>- Electron microscopy	- Increase concentration-dependent LDH release in uncoated silica.	Emerson and Davis,

		(Thermal American Fused Quartz)<br>± coated with rat protein and alveolar lining material (ALM)			- Comparable physical association by ALM-coated and uncoated silica particles with alveolar macrophages.<br>- Surfactant lipids presumably coat silica particles in the lung may reduce or delay their toxicity.	Environ Health Perspect<br>1983
WI38-VA13 human pulmonary fibroblast cells	1 μg/mL<br><br>→ 24 and 48 h	Crystalline:<br>- Min-U-Sil<br>(U.S. Silica co)	1.6 μm	- Flow cytometry<br>- Immunofluorescence staining<br>- Histopathology (Masson's trichrome staining)<br>- Gel contraction assay	- Significant increase of α-SMA expression.<br>- α-SMA localized in stress fibers.<br>- Remodeling of collagen matrix.<br>- Significant increase capacity to contract the matrix.	Hindman and Ma.<br>Arch Toxicol<br>2018
WI-38 human lung fibroblasts	- 1, 10, 50, 100, 150, 200 μg/mL<br><br>→ 4, 8, 12, 18 and 24 h	Crystalline (Sigma)	N/A	- Western Blot<br>- RT-qPCR<br>- Site-directed mutagenesis of COX-2 promoter<br>- Transient transfection and luciferase Assay<br>- EMSA (Electrophoretic Mobility Shift Assay)	- Dose- and time-dependent increase of COX-2 at gene and protein level<br>- NF-κB binding site in COX-2 promoter is responsible for silica-induced COX-2 expression<br>- NF-κB inducing kinase (NIK) and TGF-β activated kinase (TAK) are involved in the silica-mediated COX-2 expression<br>- Silica induce direct binding of NF-κB on the binding site in COX-2 promoter	Choi, et al.<br>J Environ Pathol Toxicol Oncol<br>2005
Primary human lung fibroblast culture	1, 2.5, 10, 25, 100 μg/mL<br><br>→ 6, 24, 48, 72 h	Crystalline	800 nm	- RNase protection assay<br>- Immunohistochemistry<br>- Western blot (COX-2 and mPGES)<br>- Enzyme immunoassay for prostaglandin production	- Significant increase of COX-2 gene expression (100 μg/mL).<br>- Significant increase of COX-2 and mPGES protein expression (100 μg/mL).<br>- Significant and dose-dependent increase of PGE_2 protein expression (100 μg/mL).	O' Reilly, et al.<br>Am J Lung Cell Mol Physiol<br>2005
Mouse (*ex vivo*, from C57BL/6) and human (from donors) fibroblasts cultures	(± TGF-β)	Crystalline:<br>- DQ-12 (quartz)	2.2 μm	- Cat K enzymatic activity	- Cat K downregulated in mouse (2 months after receiving 2.5 mg of silica) and human lung fibroblasts by the profibrotic growth factor TGF-β1.	Van Den Brûle, et al.<br>Respir Res<br>2005

Table S6. In vitro studies, micrometric amorphous silica.

Cell Line	Concentration and Duration	Silica Name and Supplier	Primary Particle Size	Tests	Main Results	Reference
Mouse FE1 lung epithelial cells derived from Muta™Mouse	12.5, 25, 50, 100 μg/mL<br>→ 24 h	Amorphous:<br>- SiP2μ<br>(NIST)	2 μm	- Cellular viability<br>- ROS generation<br>- Micronucleus assay	- Decreased cellular viability from 25 μg/mL for all particle types<br>- Highest potential to induce oxidative stress compared to SiNPs for all concentrations.<br>- No significant differences in micronucleus formation	Decan, et al. Mutat Res Genet Toxicol Environ Mutagen 2016
BEAS-2B human lung epithelial cells	- 50, 100, 150 or 200 μg/mL<br>± media LHC-9 (marked agglomeration) or DMEM:F12 (no agglomeration)<br>→ 20 h	Amorphous	500 nm	- Cytotoxicity (LDH release)<br>- ELISA (IL-6, IL-8)<br>- RT-PCR	- Slight increase of cytotoxicity.<br>- Significant increase and more important increase (compared with 50nm at the same surface area) of IL-6 and IL-8 in DMEM:F12.<br>- No qualitative changes in the cytokine gene-expression patterns (compared with 500 nm): suggesting effects through similar mechanisms.	Skuland, et al.<br>Toxicol In Vitro<br>2014
A549 human adenocarcinoma alveolar basal epithelial cells	20, 40, 60, 80, 100 μg/cm²<br>→ 40 h	Amorphous:<br>- Fesil microsilica<br>(Hafslund Metall)	300 nm	- Cytotoxicity (LDH release)<br>- ELISA (IL-6, IL-8)	- Significant increase of LDH release from 40 to 100 μg/cm²<br>- Significant increase of IL-6 and IL-8 (compared to crystalline, amorphous particles are more potent to induce IL-6)	Hetland, et al.<br>Hum Exp Toxicol<br>2001
RAW 264.7 mouse monocyte-macrophage	100 μg/mL<br>→ 6 and 24 h	Amorphous silica microparticles<br>(Micromod Partikeltechnologie GmbH)	3 μm	- Uptake of silica<br>- RT-qPCR (MIP-1α, MIP-2, TNF-α)	- Lower uptake in comparison with NPs<br>- Significant increase of the expression of MIP-1α and TNF-α but not for MIP-2	Inoue, et al.<br>Part Fibre Toxicol<br>2021
THP-1 human macrophage-like cells (polarized towards an M1 or M2 type)	10, 50, 100 μg/mL<br>→ 24 h	Synthesized fluorescent silica nanoparticles	1.75 μm	- Flow cytometry<br>- Fluorescence microscopy	- No significant difference observed between M1 and M2 cells regarding uptake.	Hoppstädter, et al.<br>Front Pharmacol<br>2015

- MH-S mouse alveolar macrophages<br>- Cos7 cells (not expressing NOX2)	± inhibition of NOX activity	Amorphous<br>- spherical amorphous silica<br>(Alltech)	3 μm	- Phagolysosomal leakage assay<br>- Detection of ROS<br>- Microscopy	- + inhibition of NOX activity: delayed of phagolysosomal leakage.<br>- ROS generated by NOX2 detected in phagolysosomes.<br>- Increase in phagosomal ROS with mitochondrial production of ROS late in apoptosis.	Joshi, et al.<br>Mol Biol Cell<br>2015
THP-1 human macrophage-like cells	100 μg/mL<br>→ 24 h	Aerosil, commercial fumed silica<br>(Sigma)	0.23 μm<br>± Ti and Al doping	- ELISA (IL-1β)<br>- Intracellular GSH levels<br>- Intracellular Potassium efflux	- Significant increase of IL-1β production.<br>- Significant decrease of intracellular GSH levels.<br>- Significant increase of intracellular Potassium efflux.<br>Those effects are attenuated by Ti and Al doping. Indeed, doping could reduce surface silanol density and expression of three-membered siloxane rings.	Sun, et al.<br>ACSNano<br>2015
Bone marrow-derived macrophages (BMDMs)	0.03, 0.1, 0.3 mg/mL<br>→ 24 h	Amorphous silica microparticles<br>(Micromod Partikeltechnologie GmbH)	- 0.3 μm<br>- 1 μm<br>- 3 μm<br>- 10 μm	- Cytotoxicity (LDH release)<br>- ELISA (IL-1β)<br>- Microscopy (internalization and lysosomal damage)<br><br>- Immunoblot analysis (inflammasome activation)	- Significant cell death only for 0.3 and 1 μm particles.<br>- Significant IL-1β secretion only for 0.3 and 1 μm particles.<br>- Particles efficiently internalized via an actin cytoskeleton-dependent pathway independent of diameter size. Lysosomal destabilization only for 0.3 and 1 μm particles.<br>- Particles induce caspase-1 activation independent of diameter size.	Kusaka, et al.<br>PLOS One<br>2014
- Mouse (*ex vivo*, from C57BL/6) alveolar macrophages<br>- J774 mouse monocyte macrophage cells	1.25, 2.5, 5, 10, 20, 40 μg/well<br><br>→ 24 h	Amorphous:<br>- VS (vitreous silica)	1.6 μm	- ELISA (pro-IL-1β or IL-1α)<br>- Western Blot	- IL-1α released by J774 cells after silica exposure correlated with the degree of lung inflammation induced *in vivo*	Rabolli, et al.<br>Part Fibre Toxicol<br>2014
- RAW 264.7 mouse monocyte-macrophage	- 1, 5, 10, 25, 50,100, 200 μg/mL	Amorphous:<br>- fused silica (Suprasil 200)	- 500 nm–10 μm	- siRNA transfection (knock down of NLRP3)<br>- ELISA (IL-1β)	- Reduction of particle-induced IL-1β release in RAW264.7 cells.<br>- Significant increase of IL-1β.	Sandberg, et al.

- Primary rat lung macrophages	(± LPS treatment)<br><br>→ 6 h	- monodisperse (Si500)	- 370 nm	- RT-qPCR<br>- Western Blot	- Rat primary macrophage more sensitive to silica exposure compared with RAW264.7	Part Fibre Toxicol 2012
- MH-S mouse alveolar macrophages<br>- RAW 264.7 mouse monocyte-macrophage	50 μg/cm^2<br>→ 4, 8, 12, 24 h	Amorphous:<br>- 3μm spherical silica particles (Grace Davison Discovery Science formally Alltech)<br>- 1μm spherical silica particles (Spherotech Inc)	- 3 μm<br><br>- 1 μm	- Cell death assay<br>- Western Blot (quantification of cleaved/uncleaved caspase-3 ratio)<br>- Endosome-Phagosome fusion and endo-lysosomal leakage	- Significant increase of cell death for 3 μm in MH-S cells.<br>- Activation of the apoptotic pathway for 3μm in MH-S cells.<br>- Endolysosomal leakage for 3 μm in MH-S cells.	Costantini, et al.<br>PLOS One<br>2011
- RAW 264.7 mouse monocyte-macrophage<br>- Rat BAL macrophages	15, 50 or 100 μg/mL<br>→ 6 or 18 h	Aerosil OX50 (Degussa AG)	N/A<br>± ascorbic acid pretreatment	- COX-2 expression	- Significant and dose-dependent COX-2 synthesis but no changes with acid ascorbic pretreatment (amorphous silica unable to chemically interact with ascorbic acid))	Scarfi, et al.<br>The FEBS Journal<br>2006
BAM bovine alveolar macrophages cells	200 μg/mL<br><br>→ <10 min	Amorphous:<br>- microfine precipitated silica gel (Philadelphia Quartz Company)	<10 μm	- Flow cytometry:<br>. cytosolic free calcium concentration [Ca^{2+}]i<br>. cytosolic pH (pHi)<br>. plasma membrane potential	- Significant increase of cytosolic free calcium concentration [Ca^{2+}]i<br>- Significant decrease in pHi<br>- Significant increase of PMP<br>- Existence of silica-activated transmembrane ion exchange involved in cytotoxicity by Ca^{2+} dependent pathway	Tarnok, et al.<br>Anal Cell Pathol<br>1997
Primary human lung fibroblast culture	1, 2.5, 10, 25, 100 μg/mL<br><br>→ 6, 24, 48, 72 h	Amorphous	N/A	- RNase protection assay<br>- Immunohistochemistry<br>- Western blot (COX-2 and mPGES)<br>- Enzyme immunoassay for prostaglandin production	- Significant increase of COX-2 gene expression (10 μg/mL).<br>- Significant increase of COX-2 and mPGES protein expression (10 μg/mL).<br>- Significant and dose-dependent increase of PGE_2 protein expression (10–100 μg/mL).<br>-Those increases are more potent compared to crystalline silica	O' Reilly, et al.<br>Am J Lung Cell Mol Physiol<br>2005

Table S7. In vitro studies, nanometric amorphous silica.

Cell Line	Concentration and Duration	Silica Name and Supplier	Primary Particle Size	Tests	Main Results	Reference
BEAS-2B human lung epithelial cells	- Si10: 12.5, 25 and 50 µg/mL<br>- Si50: 50, 100 and 200 µg/mL<br>→ 20 h<br><br>± siRNA transfection and gene silencing for SR-B1, LOX-1, SREC-1 and CXCL16	SiNPs (Kisker Biotech)	- 10 nm<br>- 50 nm	- ELISA (IL-6, CXCL-8, IL-1α and IL-1β)<br><br>- Analysis of TGF-α release<br><br>- Western (p38, JNK, p65)	- Marked interleukins (IL-6, CXCL-8, IL-1α and IL-1 β) responses with Si10 and Si 50. Transient KO of SR-B1, LOX-1 and CXCL16 reduced the SiNPs induced cytokine response.<br>- Important TGF-α release with Si10 and Si 50. Transient KO of SR-B1 and CXCL16 reduced the SiNPs induced TGF-α response.<br>- Phosphorylation of MAPKs (p38 and JNK) and p65 with Si10 and Si 50. Transient KO of SR-B1 induced no significant reductions of phosphorylation.	Refsnes, et al. Toxicol Lett 2021
BEAS-2B human lung epithelial cells	- 2, 10, 50 µg/mL<br>→ 24 h	SiNPs (synthesized with Stöber method)	- 57.7 nm	- UPLC-MS analysis (ultraperformance liquid chromatography-mass spectrum)<br>- TEM analysis<br><br>- Mitochondrial stress analysis<br>- Intracellular ROS assay<br>- ELISA<br><br>- RT-qPCR	- Five metabolic pathways significantly perturbed but in particular glutathione metabolism and pantothenate and coenzyme A biosynthesis (top two metabolic pathways)<br><br>- Abnormality in mitochondrial structure and mitochondrial dysfunction<br>- Inhibition of cellular respiration and ATP production<br>- Dose-dependent ROS-generation<br>- Elevation of 8-OHdG (correlated with ROS accumulation) and decrease of phospholipids screened through metabolic analysis probably responsible of secondary response with oxidative DNA damage and cell membrane dis-integrity<br>- Downregulation of NRF2 signaling =	Zhao, et al. Chemosphere 2021

					downregulation of intracellular antioxidant responses and GSH depletion	
BEAS-2B human lung epithelial cells	-7.9 mg/mL (20 nm)<br>-11.5 mg/mL (50 nm)<br>→ 24 h	Amorphous<br>(Korea Research Institute of Standards and Science)	- 20 nm<br>- 50 nm	- Screening for particle size function	- Significant increase secretion of inflammatory mediators (TNF-α, IL-6, IL-8 and CXCL1) for Si20nm.	Han, et al.<br>Toxicol Appl Pharmacol<br>2020
NCI-H292 human airway epithelial cell line	1.5625, 3.125, 6.25, 12.5 μg/mL<br>→ 12 h	SiNPs<br>(Sigma-Aldrich)	5–15 nm	- RT-PCR (IL-1β, TNF-α, IL-6, IL-8)<br>- Immunoblot (TXNIP, NLRP3, ASC, caspase-1, IL-1β)	- Significant increase of mRNA expression levels of inflammatory cytokines.<br>- Concentration-dependent elevation of proteins TXNIP, NLRP3 and IL-1β.	Ko, et al.<br>Regul Toxicol Pharmacol<br>2020
- BEAS-2B human lung epithelial cells	12.5, 25, 50, 100, 200 μg/mL<br>± PJ34 treatment (PARP inhibitor)<br>→ 3, 24 or 48 h	SiNPs<br>(Sigma-Aldrich)	10–20 nm	- Cytotoxicity (CCK-8)<br>- RT-qPCR (IL-1β, IL-6, CXCL-1, CXCL-8)<br>- Lysosomal degradation capacity<br>- Intracellular Ca^{2*} measurement	- Significant decrease of cell viability.<br>- Significant increase of pro-inflammatory cytokines.<br>- Impairment in lysosome function.<br>- Impairment in autophagic flux.<br>Inhibition of PARP and TRMP2 channel suppressed SiNPs-induced lysosome impairment and autophagy dysfunction but also inflammatory response.	Wang, et al.<br>Part Fibre Toxicol<br>2020
BEAS-2B human lung epithelial cells	50,100,150, 200 μg/mL<br>→ 20 h	Amorphous	50 nm	- ELISA (CXCL8, IL-6 and TGF-α)<br>- Cytotoxicity (propidium iodide)<br>- ROS measurements<br>- Expression of mRNA for HO-1, CXCL8 and IL-6<br>- Suppression of DUOX-1 by siRNA<br>(NADPH oxidase analogue)	- Significant increase of CXCL8 and IL-6.<br>- Very low cytotoxicity<br>- Time-dependent ROS formation.<br>- Postponed increase in expression of mRNA HO-1.<br>- siDUOX-1 reduced Si50nm induced CXCL8 but not IL-6.<br>- p38 and p65 phosphorylation inhibited by siDUOX-1.<br>Postponed increase in expression of protein HO-1.	Refsnes, et al.<br>Basic Clin Pharmacol Toxicol<br>2019

				- Western Blot analysis (phosphorylation of MAPK p38, NF-κ p65 and protein HO-1)		
- BEAS-2B human lung epithelial cells<br>- HBEC3-KT human bronchial epithelial cells	50, 100, 150, 200 μg/mL<br>→ 20 h (ELISA)<br>or 1–8 h (Western analysis)<br>or 1–6 h (FG)	Amorphous	- 10 nm<br>- 50 nm	- Cytotoxicity (LDH release)<br>- ELISA (CXCL8, IL-6, TGF-α)<br>- PCR (CXCL8, IL-6)<br>- Western Blot analysis	- Significant release of LDH for Si10nm (compared to Si50nm).<br>- Significant and more potent increase of CXCL8 and IL-6 (gene and protein) for Si10nm (compared to Si50nm). TACE-mediated TGF-α release and NF-κB activation seem to be important triggering mechanisms for both particles.<br>- p38 phosphorylation seem to be important triggering mechanism for both particles.	Lag, et al. Basic Clin Pharmacol Toxicol 2018
- 16 hBE human airway epithelial cells<br>- Primary cultured mTEC (mouse tracheobronchial epithelial cells)	- 300 μg/mL	Amorphous:<br>- SM30 Ludox (colloïdal)	- 10.2 nm	- Intracellular Ca^{2+} imaging experiments<br>- Ciliary beat frequency measurements (mTEC)	- Inhibition of cation channel TRV4 by SiNPs (16 hBE and mTEC). Significant increase in basal [Ca2+], but in a TRPV4-independent manner.<br>- Decrease of ciliary beat frequency (TRPV4-mediated) by SiNPs.	Sanchez, et al.<br>Part Fibre Toxicol 2017
A549 human adenocarcinoma alveolar basal epithelial cells	50 μg/mL<br>± TGF-β1<br><br>→ 4 h	Amorphous:<br>-SiNPs<br>(Kisker Biotech)	-10 nm<br>-100 nm	- Immunofluorescence analysis<br>- Epithelial-Mesenchymal Transition (EMT)<br>- Western Blot analysis:<br>. E-cadherin: epithelial marker, vimentin: mesenchymal marker)<br>. Smad2 (fibrogenic pathway)	- Significant colocalization of SiNP-100 and TGF-β1 on the membrane.<br>- Induction of EMT in cells treated with SiNP-100 incubated with TGF-β1.<br>- Higher expression of vimentin and weaker expression of E-cadherin in cells treated with SiNP-100 incubated with TGF-β1.<br>- Increased and prolonged phosphorylation of Smad2.	Wang, et al.<br>ACS Nano 2017
Mouse FE1 lung epithelial	12.5, 25, 50, 100 μg/mL	Amorphous:<br>- SiNP12		- Cellular viability	- Decreased cellular viability from 25 μg/mL for all particle types.	Decan, et al.

cells derived from Muta™ Mouse	→ 24 h	- SiNP5-15<br>- SiNP10-20<br>(Sigma-Aldrich)	- 12 nm<br>- 5–15 nm<br>- 10–20 nm	- ROS generation<br>- Micronucleus assay<br>- Cellular internalization<br>- DNA microarrays (biological pathway and functional perturbations)	- Significant increase of ROS generation.<br>- Size-dependent increase in micronucleus formation.<br>- Internalization and lysosomal rearrangements in the cytoplasm of SiNP12 treated cells.<br>- Significant changes in the expression of genes implicated in lysosomal functions in SiNP12 treated cells.	Mutat Res Genet Toxicol Environ Mutagen 2016
A549 human adenocarcinoma alveolar basal epithelial cells	10 μg/mL<br><br>→ 24 h	Amorphous:<br>- nano-SiO_2<br>(Shanghai Yito Bio-instrument Co)	39.6 nm	- ROS generation<br>- Intracellular glutathione (GSH) content<br>- Measurements of antioxidant enzyme activities:<br>. SOD activity<br>. GSH-Px activity<br>- Determination of malondialdehyde (MDA) content<br>- DNA damage by alkaline comet assay	- No significant difference in all parameters of intracellular oxidative stress.<br>- No significant difference in DNA damage.	Lu, et al.<br>Ecotoxicol Environ Saf 2015
BEAS-2B human lung epithelial cells	- 50, 100, 150 or 200 μg/mL<br>± media LHC-9 (marked agglomeration) or DMEM:F12 (no agglomeration)<br><br>→ 20 h	Amorphous<br>(Kisker-Biotech)	- 50 nm	- Cytotoxicity (LDH release)<br>- ELISA (IL-6, IL-8)<br><br>- RTPCR	- Slight increase of cytotoxicity.<br>- Significant increase and more important increase (compared with 500 nm at the same concentration) of IL-6 and IL-8 in both media.<br>- No qualitative changes in the cytokine gene-expression patterns (compared with 500nm): suggesting effects through similar mechanisms.	Skuland, et al.<br>Toxicol In Vitro 2014
- H441 human adenocarcinoma	- 0.6-6000 μg/mL<br><br>→ 4 h	Amorphous:<br>- Ludox TM-40<br>(Sigma-Aldrich)	-31.2 nm	- Cytotoxicity (MTS assay, LDH release and TER measurement)	- Significant increase of toxicity with 600 and 6000 μg/mL for both particles (Co-culture model less sensitive to apical particle	Kasper, et al.<br>Part Fibre Toxicol

epithelial cell line<br>- ISO-HAS-1 human micro-vascular endo-thelial cell line<br>(± co-cultured)		- NexSil20 aqueous silica<br>(Nyacol Nano Tech-nologies)	-31.4 nm	- ELISA (sICAM-1, IL-6, IL-8)<br>- Apoptosis markers analysis (Human apop-tosis Array Kit)	exposure).<br>- Increase more sensitive in the co-culture model with release of IL-6 and IL-8 to the basolateral side from 6 to 6000 μg/mL.<br>- Apoptosis markers (intrinsic pathway) up-regulated in the co-culture model.<br>Significant increase of DNA damage, hy-poxia and ER-stress individual markers with 600 μg/mL of NexSil20.	2011
A549 human adenocarci-noma alveolar basal epithe-lial cells	- 50,100, 200, 400 μg/mL<br>→ 48 h	Amorphous:<br>- Spherical, porous<br>- Spherical<br>(NanoAmor)	-10 nm<br>-80 nm	- Cytotoxicity (MTT and LDH release)<br>- Intracellular ROS meas-urements<br>- Lipid peroxidation (LPO)<br>- Intracellular GHS measurements<br>- Glutathione reductase and glutathione peroxi-dase assay	- Significant and dose-dependent (from 100 to 400 μg/mL) increase for both sizes of par-ticles.<br>- ROS and LPO induced in a dose-depend-ent manner for both sizes of particles.<br>- Slight depletion of GSH and of enzymes activities for both sizes of particles.	Akhtar, et al.<br>Toxicology<br>2010
BEAS-2B hu-man lung epi-thelial cells	1 mg/l<br>→ 24 h	- Fumed SiNPs<br>- Porous SiNPs<br>(Sigma)	-7 nm<br>-5–15 nm	- Cytotoxicity (MTT as-say)<br><br>- ROS measurement and induction of antioxidant enzymes (SOD, HO-1)<br>- Western Blot (NF-B, Nrf-2, MAP kinase)	- Significant decrease of cell viability (higher for cells exposed to porous SiNPs).<br>- Significant induction of HO-1 (higher for cells exposed to porous SiNPs).<br><br>- HO-1 induction involve Nrf-2-ERK MAP kinase signaling pathway with more sensi-tive response for cells exposed to porous SiNPs (greater surface area) than those ex-posed to fumed SiNPs.	Eom, et al.<br>Toxicol In Vitro<br>2009
MLE15 mu-rine alveolar epithelial type II cells	4.7, 9.5, 18.9 μg/cm^2<br>(± MG-132 (proteosomal inhibitor) or inhibition of NF-κB and MAPK)	Amorphous<br>- fumed silica (Aer-osil200)<br>(Degussa)	12 nm	- Cytotoxicity (LDH re-lease)<br>- ELISA (MIP-2)<br>- Western Blot	- Significant and dose-dependent increase of LDH release<br>- Significant increase of MIP-2 protein for 9.5 and 18.9 μg/cm^2.	Singal and Finkelstein.<br>Exp Lung Res

	→ 24 h				+ MG-132: synergic increase.<br>+ MAPK inhibitor: significant decrease.	2005
- A549 human adenocarcinoma alveolar basal epithelial (co-cultured + insert of EA.hy926 hybrid human endothelial cell line)	5 μg/cm² or 10cm² particles SA/cm²<br><br>→ 24 h<br>(insert introduced)	Amorphous:<br>- monodisperse	-2 nm<br>-16 nm<br>-60 nm<br>-104 nm	- Cytotoxicity (LDH release)<br>- Cytokine analysis (multiplex with TNF-α, IL-6, IL-8, MIP-1α, MIP-1β)	- No damage of cellular membrane.<br>- Significant increase of all cytokines for 2 nm at both concentrations and in both compartments.<br>Significant increase of all cytokines for 60 nm at 10 cm² particle SA/cm².	Napierska, et al.<br>Toxicol Lett<br>2012
RAW 264.7 mouse monocyte-macrophage	100 μg/mL<br><br>→ 6 and 24 h	Amorphous silica nanoparticles (Micromod Partikeltechnologie GmbH)	50 nm<br>(Without surface modification*n*=plain or with amine surface modification:<br>50 nm–NH_2)	- Uptake of silica<br><br>- Endosomal ROS signal<br><br>- RT-qPCR (MIP-1α, MIP-2, TNF-α)<br>- Selective inhibition of NADPH oxidase 2 (NOX2)	- Unchanged efficacy in cellular uptake between 50 nm-NH_2 and 50 nm- plain silica<br>- Significant increase of ROS in endosomal compartments cells treated with 50 nm-plain<br>- Significant increase of the expression of MIP-1α, MIP-2 and TNF-α<br><br>- Inhibition of ROS endosomal bursts and chemokine expression	Inoue, et al.<br>Part Fibre Toxicol<br>2021
THP-1 human macrophage-like cells	- 6.25, 12.5, 25, 50 and 100 μg/mL<br>→ 24 h	SiNPs<br>(Jinhao Co)	-20–50 nm	- Cytotoxicity (LDH activity and MTT assay)<br>- Cell apoptosis assay<br>-ELISA (TGF-β, TNF-α and IL-1β)<br>- Proteomic analyses with LC-MS (liquid chromatograph-tandem mass spectrometry)	- Dose-dependent cytotoxicity (increase of LDH content) in THP-1 cells<br>- SiNPs triggered apoptosis in THP-1 cells<br>- Significant increase of inflammatory factors (TGF-β, TNF-α and IL-1β) in THP-1 cells<br>- SERPINB2 (plasminogen activator inhibitor 2) protein identified as a potential biomarker of inflammatory responses and fibrosis	Li, et al.<br>Toxicol In Vitro<br>2021
- RAW 264.7 mouse	10, 15, 20, 30, 50 μg/mL	Amorphous:<br>- fumed silica	12 nm	- Western Blot analysis<br>- ELISA	- Significant activation of MAPKs (JNK1/2, ERK1/2 and p38).	Fritsch, et al.<br>Arch Toxicol

monocyte-macrophage<br>- THP-1 human macrophage-like cells<br>- MDM human monocyte-derived primary macrophages	→ 2, 4, 6 h	(Aerosil200)<br>(Evonik)		- Cytotoxicity (LDH release)<br>- RT-qPCR	- Significant increase of TNF-α.<br>+ MAPKs (JNK1/2 and ERK1/2) inhibitors: suppression of TNF-α release.<br>- Significant time and dose-dependent increase of LDH activity from 50 μg/mL<br>- Slight significant increase of genes Hmox1, Nqo1 and Gclc (moderate anti-oxidative response).<br>Significant increase of genes Tnf, Ptgs2, Cxcl2 and Ccl2 (pro-inflammatory response).	2018
RAW 264.7 mouse monocyte-macrophage	5, 10, 20, 40 μg/mL<br>→ 24 h	Synthesized through the Stöber method	43 nm	- Cytotoxicity<br>- Cell phagocytic ability assay<br>- Western Blot	- Dose-dependent cytotoxicity.<br>- Dose-dependent decrease of phagocytic ability.<br>- Significant increase of NLRP3, IL-1β and NF-κB with a peak at 10 μg/mL.	Yang, et al.<br>Int J Nanomedicine<br>2016
- THP-1 human macrophage-like cells (polarized towards an M1 or M2 type)	10, 50, 100 μg/mL<br>→ 24 h	Synthesized fluorescent silica nanoparticles	- 26 nm<br>- 41 nm	- Cytotoxicity (MTT assay)<br>- Flow cytometry<br>- Fluorescence microscopy	- Significant decrease of cell viability for macrophages exposed to 100 μg/mL of NPs.<br>- NPs uptake enhanced in M2-polarized macrophages<br>- M2 polarization of macrophages promotes NPs internalization.	Hoppstädter, et al.<br>Front Pharmacol<br>2015
Bone marrow-derived macrophages (BMDMs)	0.03, 0.1, 0.3 mg/mL<br>→ 24 h	Amorphous silica nanoparticles (Micromod Partikeltechnologie GmbH)	- 30 nm<br>- 100 nm	- Cytotoxicity (LDH release)<br>- ELISA (IL-1β)<br>- Microscopy (internalization and lysosomal damage)<br>- Immunoblot analysis (inflammasome activation)	- Significant cell death only for both sizes of NPs.<br>- Significant IL-1β secretion for both sizes of NPs.<br>- NPs efficiently internalized via an actin cytoskeleton-dependent pathway and lysosomal destabilization.<br>- NPs induce caspase-1 activation.	Kusaka, et al.<br>PLOS One<br>2014

- Mouse (*ex vivo*, from C57BL/6) alveolar macrophages<br>- J774 mouse monocyte macrophage cells	1.25, 2.5, 5, 10, 20, 40 µg/well<br><br>→ 24 h	Amorphous:<br>- Stöber Silica<br>- fumed silica (Aerosil 200) (Sigma-Aldrich)	-12 nm<br>-12 nm	- ELISA (pro-IL-1β or IL-1α)<br>- Western Blot	- IL-1α released by J774 cells after silica exposure correlated with the degree of lung inflammation induced *in vivo*	Rabolli, et al. Part Fibre Toxicol 2014
- RAW 264.7 mouse monocyte-macrophage<br>- Primary rat lung macrophages	1, 5, 10, 25, 50,100, 200 µg/mL (± LPS treatment)<br><br>→ 6 h	Amorphous:<br>- fumed silica (Aerosil 200)<br>- monodisperse (Si50)	- 20 nm<br><br>- 65 nm	- siRNA transfection (knock down of NLRP3)<br>- ELISA (IL-1β)<br>- RT-qPCR<br>- Western Blot	- Reduction of particle-induced IL-1β release in RAW264.7 cells.<br>- Significant increase of IL-1β.	Sandberg, et al. Part Fibre Toxicol 2012
-THP-1 human macrophage-like cells (co-cultured + insert of EA.hy926 hybrid human endothelial cell line)	5 µg/cm^2 or 10cm^2 particles SA/cm^2<br><br>→24 h (insert introduced)	Amorphous:<br>- monodisperse	-2 nm<br>-16 nm<br>-60 nm<br>-104 nm	- Cytotoxicity (LDH release)<br>- Cytokine analysis (multiplex with TNF-α, IL-6, IL-8, MIP-1α, MIP-1β)	- No damage of cellular membrane.<br>- Significant increase of all cytokines for 2nm at both concentrations and in both compartments.<br>Significant increase of all cytokines for 60nm at 10cm^2 particle SA/cm^2.	Napierska, et al. Toxicol Lett 2012
MRC-5 human embryonic lung fibroblasts	6.25, 12.5, 25, 50 and 100 µg/mL<br>→ 24 h	SiNPs (Jinhao Co)	-20–50 nm	- Hydroxyproline content<br><br>- Western Blot (α-SMA and Collagen 1)<br>- Proteomic analyses with LC-MS (liquid chromatograph-tandem mass spectrometry)	- Significant increase of hydroxyproline content in MRC-5 cells at 100 µg/mL dose.<br>- Significant increase of α-SMA and Collagen 1, fibroblast-myofibroblast trans-differentiation markers, in MRC-5 cells<br>- SERPINB2 (plasminogen activator inhibitor 2) protein identified as a potential biomarker of inflammatory responses and fibrosis	Li, et al. Toxicol In Vitro 2021

V79 hamster Chinese lung fibroblasts cells	12.5, 25, 50, 100 μg/cm² → 24 h	Amorphous:<br>- Pyr20 (pyrogenic)<br>- Pyr25/70 (pyrogenic)<br>- Pre20 (precipitated)<br>- Col15 (colloïdal)<br>- Col40/80 (colloïdal)	- 19 nm<br>- 25 and 71 nm<br>- 19 nm<br>-15 nm<br>- 38 and 79 nm	- Cellular viability<br>- Caspase-3 assay<br>- FPG-modified comet assay<br>- ROS generation<br>- Micronucleus assay<br>- Mutation induction assay (HPRT locus)	- Significant decrease of cell viability for Pyr20, Pre20 and Col15.<br>- Significant induction of apoptosis for Pyr20, Pre20 and Col15.<br>- Significant production of DNA damages for Pyr20 and Col15.<br>- No significant differences for intracellular ROS generation, micronuclei or genomic mutation.	Guichard, et al. Toxicol Ind Health 2016
Pulmonary surfactant extract from porcine lung	0.25, 0.5,1 mg/ml	Amorphous (Macklin Biochemical Co.)	50 nm	- Compression behavior<br>- Foaming ability	- Compression isotherm (simulate the physiological conditions of alveoli during respiration) significantly altered.<br>- Foaming ability (key indicator of maintaining interface properties of pulmonary surfactant) significantly altered.	Zhao, et al. Chemosphere 2019
